# Increasing Nonsteroidal Anti-inflammatory Drugs and Reducing Opioids or Paracetamol in the Management of Acute Renal Colic: Based on Three-Stage Study Design of Network Meta-Analysis of Randomized Controlled Trials

**DOI:** 10.3389/fphar.2019.00096

**Published:** 2019-02-22

**Authors:** Hui-Yun Gu, Jie Luo, Jun-Yi Wu, Qi-Sheng Yao, Yu-Ming Niu, Chao Zhang

**Affiliations:** ^1^Center for Evidence-Based Medicine and Clinical Research, Taihe Hospital, Hubei University of Medicine, Shiyan, China; ^2^Department of Emergency, Taihe Hospital, Hubei University of Medicine, Shiyan, China; ^3^Department of Urology, Taihe Hospital, Hubei University of Medicine, Shiyan, China

**Keywords:** acute renal colic, pain management, non-steroidal anti-inflammatory drugs, opioids, paracetamol

## Abstract

**Background:** Currently, although non-steroidal anti-inflammatory drugs (NSAIDs) were recommended for acute renal colic in the 2018 European Association of Urology guidelines, there are no specific NSAIDs and no specific routes of administration in this guideline. The clinical practice of advocating intravenous opioids as the initial analgesia is still common out of the fear of adverse events from NSAIDs.

**Objectives:** To comprehensively assess the efficacy and safety of NSAIDs, opioids, paracetamol, and combination therapy for acute renal colic.

**Methods:** Ovid MEDLINE, Ovid EMbase, the Cochrane Library, Clinical Trials Registry Platform for Clinicaltrials.gov, and WHO International Clinical Trials Registry Platform were searched through February 2, 2018. Two reviewers selected all randomized controlled trails (RCTs) regarding NSAIDs, opioids, paracetamol, combination therapy, and placebo were identified for analysis. We designed a three-stage strategy based on classification and pharmacological mechanisms in the first stage, routes of administration in the second stage, and specific drug branches with different routes in the third stage using network meta-analysis. The pain variance at 30 min was seen as the primary outcome.

**Results:** 65 RCTs with 8633 participants were involved. Comparing different classification and pharmacological mechanisms, combination therapy with more adverse events was more efficient than NSAIDs for the primary outcomes. Opioids gave rise to more nonspecific adverse events and vomiting events. NSAIDs were superior to opioids, paracetamol, and combination therapy after a full consideration of all outcomes. Comparing different routes of administration, NSAIDs with IV or IM route ranked first from efficacy and safety perspective. Comparing different specific drug branches with different routes, ibuprofen via IV route, ketorolac via IV route and diclofenac via IM route were superior for the management of acute renal colic. The results from diclofenac using IM route were more than those from ibuprofen used with IV route and ketorolac with IV route.

**Conclusions:** In patients with adequate renal function, diclofenac via the IM route is recommended for patients without risks of cardiovascular events. Ibuprofen and ketorolac with IV route potentially superior to diclofenac via IM route remain to be investigated. Combination therapy is an alternative choice for uncontrolled pain after the use of NSAIDs.

## Introduction

Renal colic, most commonly caused by kidney stones, is a urological emergency with a 10–15% lifetime prevalence (Fisang et al., 2015). Kidney stones cause pain by the obstruction of urinary flow (Leong and Mackie, 2014) and increasing pressure of the urinary tract, which promotes synthesis and release of prostaglandins (Asgari et al., 2012). Pain management is urgently required for patients due to unbearable and intense pain from renal colic (Shokeir, 2002).

Current common management for renal colic is the administration of non-steroidal anti-inflammatory drugs (NSAIDs) and opioids (Zamanian et al., 2016). The NSAIDs can relieve pain mainly by inhibiting the cyclooxygenase enzyme which induces a subsequent inhibition in prostaglandin synthesis (Vane, 1971). Because of satisfactory pain relief by both drugs (Kariman et al., 2015; Shirazi et al., 2015; Faridaalaee et al., 2016), the current choice of management mainly depends on the preferences of clinicians who lack a consensus about pain management regarding efficacy, safety, and other factors. Adverse events such as renal failure and gastrointestinal bleeding were reported after the use of NSAIDs (Cordell et al., 1994). Although opioids are inexpensive and potent and have a rapid pain relief, the risks of respiratory depression and drug dependency have to be considered (Bektas et al., 2009). Previous studies (Cordell et al., 1994, 1996; Holdgate and Pollock, 2005; Bektas et al., 2009) have found that treatment with NSAIDs is superior over opioids, which is entirely different from the conclusion of some other studies (Marthak et al., 1991; Curry and Kelly, 1995; Shirazi et al., 2015) and a recent meta-analysis (Pathan et al., 2018) found that the superiority of NSAIDs over opioids for pain relief was uncertain. In addition, routes of administration for acute renal colic also deserve to be stressed. Intravenous administration of either NSAIDs or opioids is the common route for pain relief in acute renal colic (Mozafari and Masoumi, 2017). However, this route is associated with adverse events and requires more time. Currently, there are a few meta-analyses (Pathan et al., 2018) and studies comprehensively investigating the effects of routes of administration for management of acute renal colic, which could decrease the efficacy and increase adverse events from inappropriate routes. Additionally, acetaminophen, also known as paracetamol, has been reported to work by inhibiting a third isoform of cyclooxygenase (COX-3) (Chandrasekharan et al., 2002) and to have a good analgesic effect (Azizkhani et al., 2013; Masoumi et al., 2014). In this study, paracetamol was also our focus. Although combination therapy achieved rapid pain relief, there were more adverse events compared with single medication regimen (Safdar et al., 2006; Asgari et al., 2012).

Based on the above controversies and latest evidences for drug intervention for the treatment of acute renal colic, we performed a network meta-analysis to compare the efficacy and safety of route of administration from NSAIDs, opioids, paracetamol, combination therapy and placebo, in order to provide the optimal therapy and evidence for the management of acute renal colic.

## Methods

### Protocol

The protocol of network meta-analysis was developed using the Preferred Reporting Items for Systematic reviews and Meta-Analysis for Protocols (PRISMA-P) (Moher et al., 2015) and registered with PROSPERO (CRD42018087906). The methods are briefly described here.

### Literature Search

We searched Ovid MEDLINE, Ovid EMbase, the Cochrane Library, Clinical Trials Registry Platform for Clinicaltrials.gov, and WHO International Clinical Trials Registry Platform through February 2, 2018 for eligible literature focusing on the comparison of the efficacy and safety of NSAIDs, opioids, paracetamol, combination therapy and placebo in treatment for acute renal colic. In addition, we also obtained studies from the references of relevant reviews, meta-analyses, clinical guidelines and included studies. The search strategy is described in Supplement Method 1.

### Inclusion and Exclusion Criteria

Included studies met these criteria: (1) all participants were over 16 years old; (2) all participants were diagnosed with acute renal colic with a pain severity of moderate to severe and the pain was less than 12 h in duration; (3) the interventions were limited to NSAIDs, opioids, paracetamol, combination therapy and placebo; the combination therapy was defined as a combination of the pairing of NSAIDs, opioids and paracetamol, regardless of the classification and pharmacological mechanisms, routes of administration or specific drug branches; (4) all studies included at least one outcome which comprised pain variance at 30 min, failure of complete and over 50% pain relief at 30 min, the need for rescue analgesia, nonspecific acute adverse events and vomiting as an adverse event. The pain variance at 30 min was seen as the primary outcome, and others were secondary outcomes; (5) all studies without language restrictions were RCTs.

If data could not be extracted or obtained by contact with the author, the study was excluded. If the study was a duplicate, it was also excluded.

### Data Collection and Disposal

Five authors independently collected relevant information including study design, patient characteristics, interventions, comparisons, and outcomes. For any missing data, particularly study design or outcomes, we contacted the original study authors for clarification. Conflicts on the extracted data were resolved by discussion and consultation with an expert.

### Three-Stage Study Design

To make better use of the data, further investigate the efficacy, safety, routes of drug administration and to find the optimal regimen for treating acute renal colic, we carried out this network meta-analysis using three-stage study design. The first stage compared the efficacy and safety among NSAIDs, opioids, paracetamol, combination therapy, and placebo according to the classification and pharmacological mechanism of these interventions. In the second stage, we investigated the effect of routes of administration on treatment of acute renal colic and we compared intravenous (IV), intramuscular (IM), per oral (PO), per rectal (PR), subcutaneous (SC), and sublingual (SU) routes. To determine the optimal drug and corresponding route, the third stage compared these interventions based on different drug branches and routes of administration.

### Quality Assessment of Included Trials

The methodological qualities of included trials were independently assessed by two authors according to the Cochrane Collaboration's tool for assessing risk of bias (Higgins, 2011). We assessed random sequence generation, allocation concealment, blinding of participants and personnel, blinding of outcome assessors, incomplete outcome data, selective reporting and other sources of bias. Disagreement was resolved by discussion and consultation with an expert if necessary.

### Statistical Analysis

For pain scores, different measuring tools were used in the included studies, including the visual analog scale (VAS) 100 mm (100 score), 10 cm length (10 score) and numerical rating scale (NRS-11) (10 score). Patients gave a score representing their degree of pain using these scales. We converted different scores obtained from different scales to scores from a 100-score scale for data consistency and calculation. Dichotomous and continuous outcomes were expressed as odds ratio (OR) with 95% credible interval (CrIs) and mean difference (MD) with 95% CrIs (Deeks, 2002; Higgins, 2011). Heterogeneity between studies was assessed using chi-squared tests, in which the significance level was set to *P* <0.1, as well as the I^2^ statistic(Higgins, 2011). I^2^ values of ≥40% are interpreted as significant heterogeneity and we used a random-effects model to conduct the meta-analysis; for I^2^ < 40%, a fixed-effect model was used instead (Higgins, 2011). All direct treatment effects were estimated using RevMan 5.3 software.

Network meta-analyses are able to provide reliable evidence for direct and indirect multiple-intervention comparisons (Lu and Ades, 2004). For it found consistency of network meta-analysis, the bayesian hierarchical randomized consistency model (Dias et al., 2013) was employed. Otherwise, the design-by-treatment interaction model with random inconsistency effects (Jackson et al., 2014) was adopted. In the network meta-analysis, we used non-informative priors with vague normal (mean 0, variance 10,000) and uniform (0–1) prior distributions for parameters such as the means and standard deviations (Lu and Ades, 2004). Various levels of prior distribution were applied in sensitivity analyses. First, 50,000 simulations were performed, and then we generated an additional 10,000 simulations with three sets of different initial values and sheared the first 50,000 simulations as the burn-in period in our model. We used the Brooks-Gelman-Rubin statistical method for assessing model convergence. Based on 50,000 simulations with 50 thin, the point estimate adopted the median of the posterior distribution, and the corresponding 95% CrIs used the 2.5th and 97.5th percentiles of the posterior distributions, which were interpreted in a similar fashion as conventional 95% confidence intervals. To discuss sources of heterogeneity and inconsistency and their influence on the results, pain scales, including VAS 10 mm or VAS 100 mm, were employed using subgroup analysis. Additionally, excluding placebo, single-blinded and unblinded studies, and zero event study were viewed as a sensitivity analysis from the second stage in a *post hoc* comparison. The comparison-adjusted funnel plot was used for the presence of small-study effects (Chaimani and Salanti, 2012). Analyses were conducted using WinBUGS 1.4.3 and R 3.1.1 software.

Based on the underlying assumption of transitivity in the network, conflicts may exist between pairwise comparisons and the distribution of effect modifiers (Salanti, 2012). Inconsistency between direct and indirect evidence suggested that transitivity is not apparent between the results (Song et al., 2011). The “loop inconsistency” method (Song et al., 2011) is apparent when the treatment effects around a loop do not conform to the consistency equations. The standard criterion (Veroniki et al., 2013) states that when 95% CrIs including 0 are reported, insignificant disagreement exists. To summarize probabilities, we used the surface under the cumulative ranking curve (SUCRA) to provide a summary statistic for the cumulative ranking (Salanti et al., 2011). By definition, SUCRA values reflect the efficacy or safety of an intervention, and thus, the rank-heat plot with larger SUCRA scores implies more effective or safer interventions (Veroniki et al., 2016). Ethical considerations were not involved in this study. The latest Preferred Reporting Items for Systematic Reviews and Meta-Analyses (PRISMA) extension statement for the reporting of systematic reviews and network meta-analysis was used (Hutton et al., 2015).

## Results

### Characteristics of Included Trials

**Figure 1:**
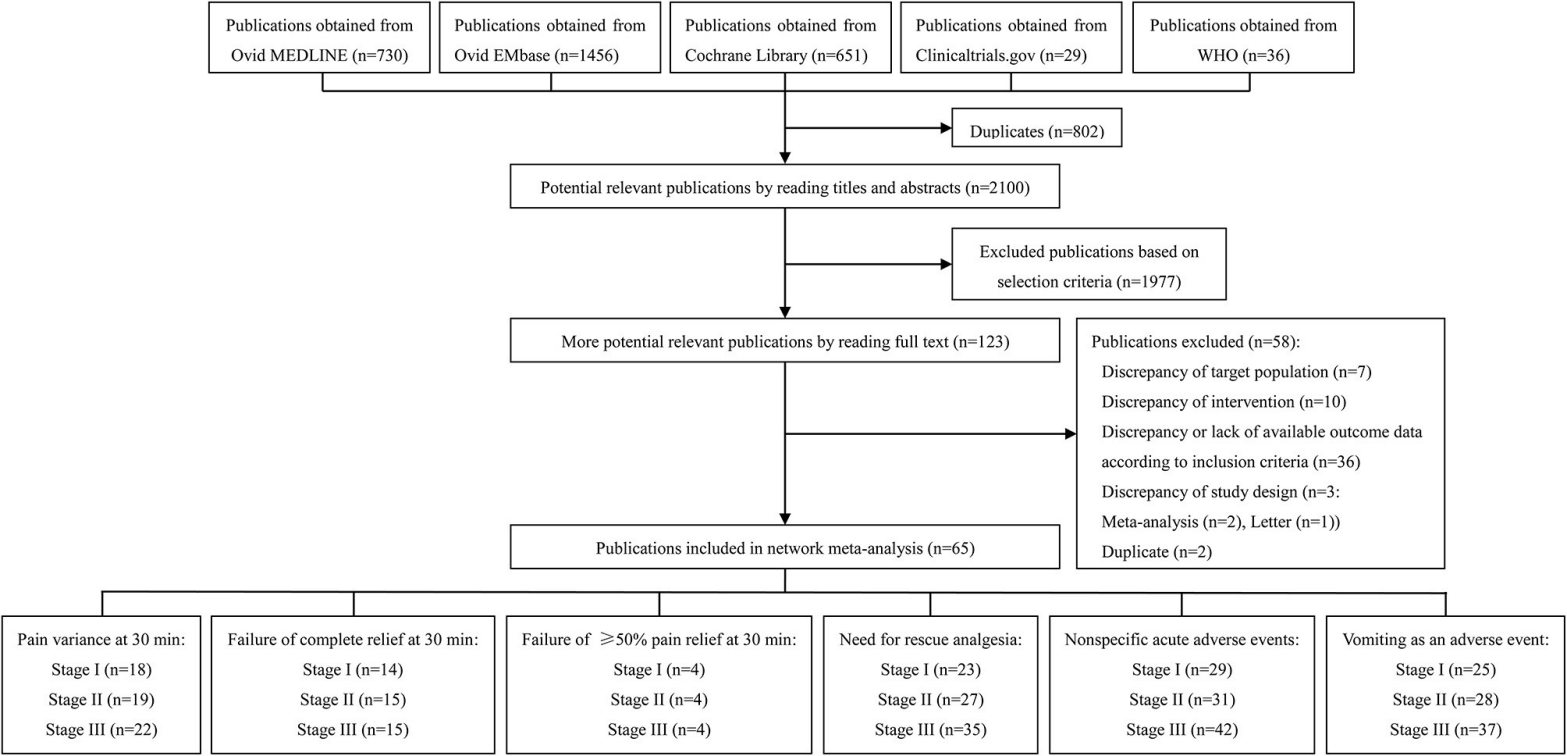
Summary of trial identification and selection.

Our systematic literature search identified 2,100 potential publications (Figure 1). Based on the inclusion and exclusion criteria, we obtained quantitative data for our network meta-analysis by reading all titles, abstracts, and full text evaluations. We ultimately included 65 RCTs with 8,633 participants (Supplement Table 1) and the reasons for exclusion of studies are described in Supplement Method 2. Figure 1 showed the number of included RCTs with different outcomes across three stages. See the details section in Figure 1.

### Quality Assessment of Included Trials

The results are shown in Supplement Figure 1. Most trials had one to six unclear risks of seven items. Twenty-seven trials had one to four high risks of seven items. All the items of 8 trials were low risk. About 50% trials had a low risk of bias for random sequence generation and 45% had a low risk of bias for allocation concealment. 68%, 38%, 80%, 55% and 47% of trials had a low risk of bias for blinding of participants and personnel, blinding of outcome assessors, incomplete outcome data, selective reporting and other sources, respectively.

### Outcomes of Network Meta-Analysis From Three Stages

#### The Result of Network Meta-Analysis From Stage I

**Figure 2:**
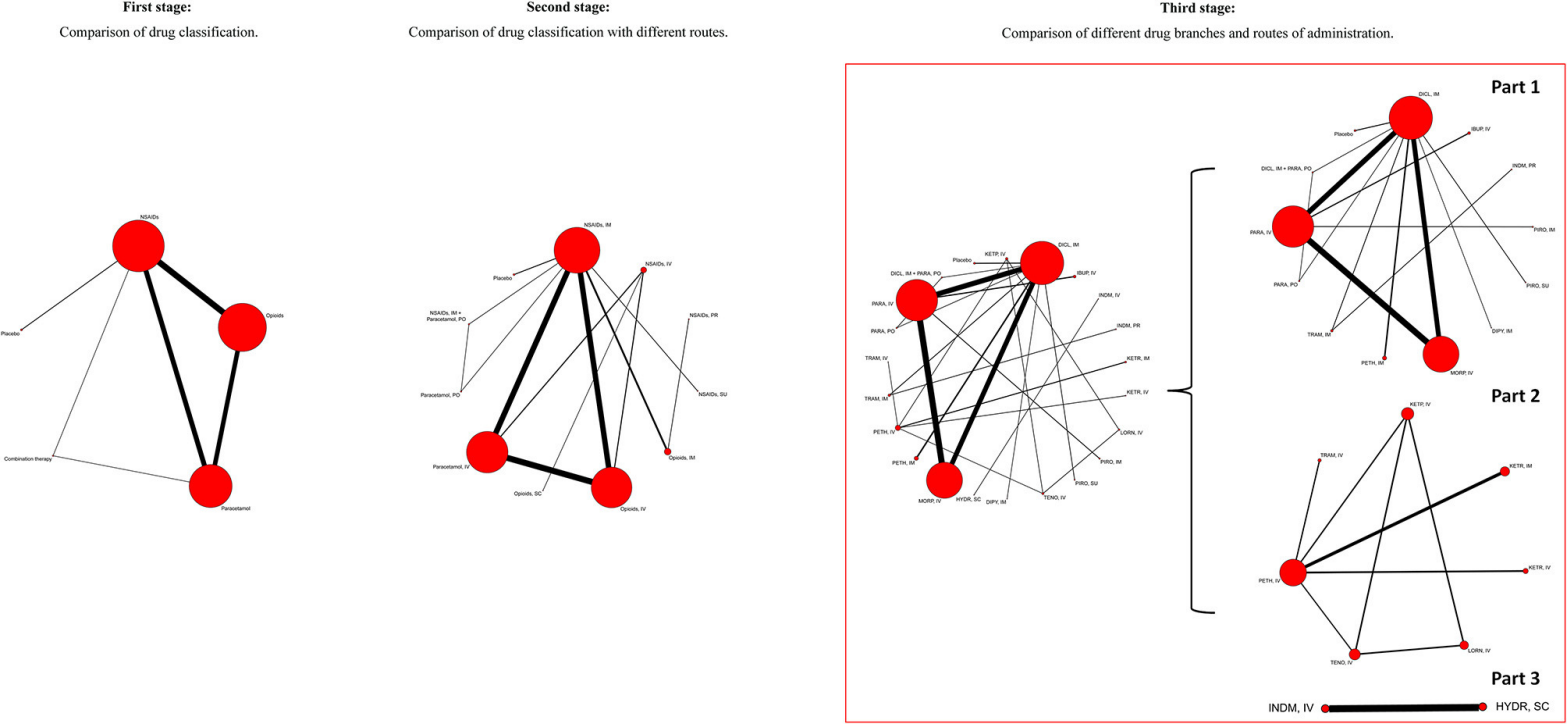
The network of eligible studies with NSAIDs, opioids, paracetamol, combination therapy, and placebo for pain variance at 30 min from first, second, and third stage. The node sizes correspond to the number of trials that investigated the treatments. Directly comparable treatments are linked with a line, and the thickness of the line corresponds to the sum of the sample size in each pairwise treatment comparison. In third stage, the rude global network plot was natural punitively divided into three unconnecting intervention structures. NSAIDs, Non-steroidal anti-inflammatory drugs; IM, Intramuscular route; IV, Intravenous route; PO, Per oral route; PR, Per rectal route; SC, Subcutaneous route; SU, Sublingual route; DICL, Diclofenac; DIPY, Dipyrone; HYDR, Hydromorphine; IBUP, Ibuprofen; INDM, Indomethacin; KETP, Ketoprofen; KETR, Ketorolac; LORN, Lornoxicam; MORP, Morphine; PARA, Paracetamol; PETH, Pethidine; PIRO, Piroxicam; TENO, Tenoxicam; TRAM, Tramadol.

**Figure 3:**
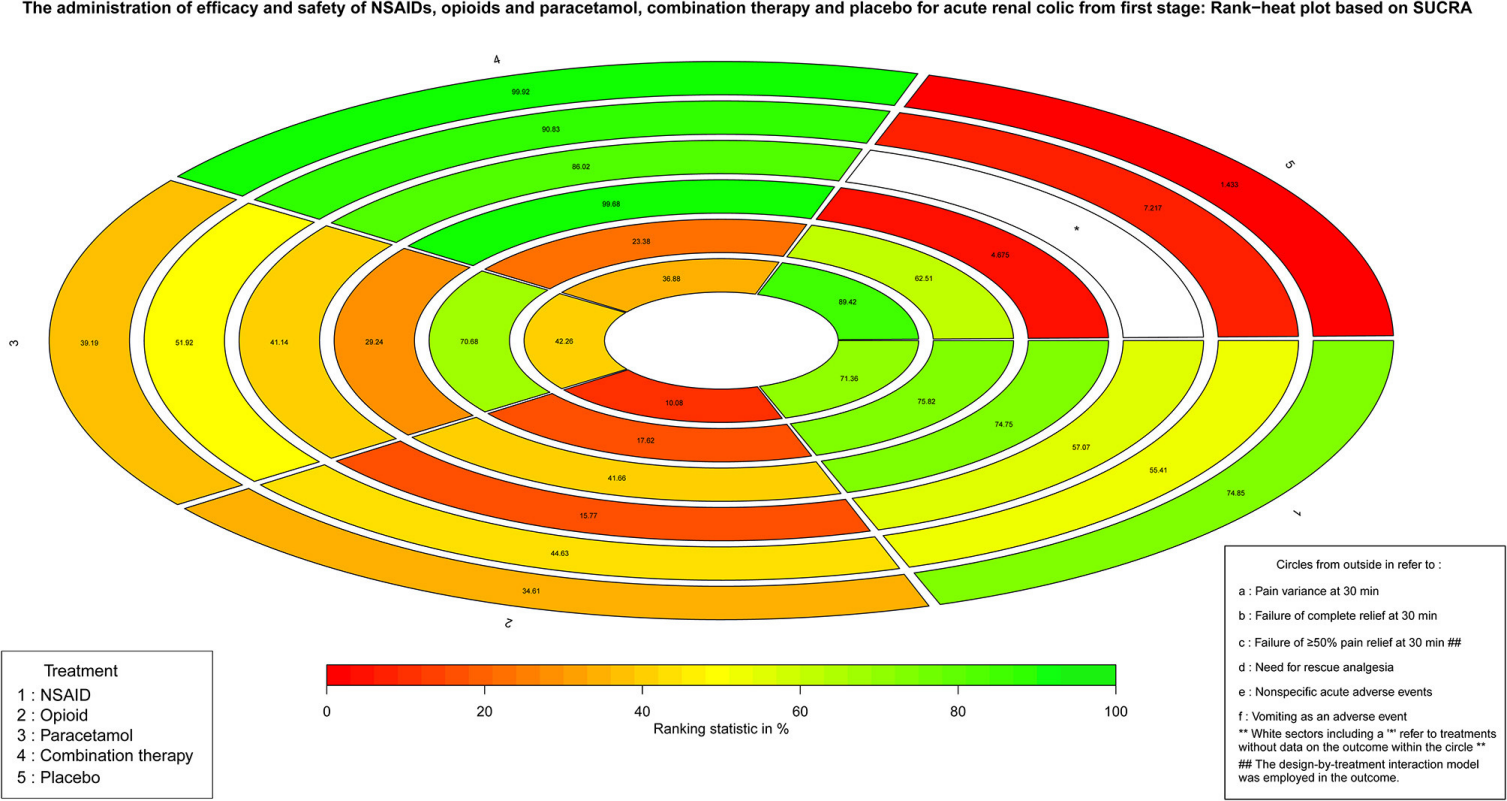
The Rank-heat plot of SUCRA for all outcomes from first stage. Each sector is colored according to the SUCRA value of the corresponding treatment and outcome. The scale consists of the transformation of three colors red (0%), yellow (50%), and green (100%), and each color is associated with a different pattern. Uncolored sectors show that the underlying treatment was not included in the network meta-analysis for the particular outcome. SUCRA: Surface under the cumulative ranking.

Based on the classification and pharmacological mechanism of five interventions, Figure 2 shows that the network of eligible studies with NSAIDs, opioids, paracetamol, combination therapy and placebo for pain variance at 30 min and other outcomes are shown in Supplement Figure 2. Supplement Figure 3 shows the results of loop consistency for all outcomes. Table 1 show that NSAIDs are superior to opioids, paracetamol and placebo in pain variance at 30 min and failure of complete relief at 30 min, and Supplement Table 2 show that NSAIDs are superior to opioids, paracetamol and placebo failure of ≥50% pain relief at 30 min and need for rescue analgesia. However, combination therapy is more effective than NSAIDs in the aforementioned four outcomes. Supplement Table 3 illustrates that NSAIDs led to fewer nonspecific acute adverse events and vomiting events than opioids, paracetamol and combination therapy. In addition, the opioids led to more nonspecific acute adverse events and vomiting events than NSAIDs, paracetamol, combination therapy and placebo. After a full consideration of all outcomes from the rank-heat plot of SUCRA shown in Figure 3, we found that NSAIDs are superior to opioids, paracetamol, combination therapy and placebo regardless of the efficacy or safety, whereas combination therapy is more effective than NSAIDs in pain relief.

**Table 1:** The results of network meta-analysis of NSAIDs, opioids, paracetamol, combination therapy and placebo for pain variance at 30 min and failure of complete relief at 30 min from first stage.

**NSAIDs**	**0.90 (0.54, 1.51)**	**0.99 (0.25, 3.49)**	**2.53 (0.53, 11.94)**	**0.29 (0.07, 1.39)**
**−6.36 (−9.99, –2.81)**	**Opioids**	1.09 (0.29, 3.84)	2.81 (0.59, 13.24)	0.32 (0.07, 1.57)
**−5.92 (−10.47, –1.18)**	0.53 (−4.41, 5.25)	**Paracetamol**	2.57 (0.37, 19.16)	0.31 (0.04, 2.33)
**20.64 (6.79, 33.25)**	**27.02 (13.19, 39.60)**	**26.64 (13.33, 38.96)**	**Combination therapy**	0.11 (0.01, 1.06)
**−20.65 (−34.39, –6.60)**	−14.5 (−28.53, 0.48)	−14.83 (−29.50, 0.13)	**−41.29 (−60.52, –21.49)**	**Placebo**

*Comparisons between treatments should be read from left to right and the estimate is in the cell in common between the upper-left-defining treatment and the lower-right-defining treatment. For pain variance at 30 min from Stage I in the lower left corner, the mean difference (MD) lower than 0 favor the upper-left-defining treatment. For failure of complete relief at 30 min from Stage I in the upper right corner, the odds ratios (ORs) lower than 1 favor the upper-left-defining treatment. To obtain MDs for comparisons in the opposite direction, negative values should be converted into positive values, and vice versa. To obtain ORs for comparisons in the opposite direction, reciprocals should be taken. Significant results are in bold and underlined. NSAIDs, Nonsteroidal anti-inflammatory drugs*.

#### The Result of Network Meta-Analysis From Stage II

Based on routes of drug administration for acute renal colic, Supplement Figure 4 shows the results of direct comparison of NSAIDs, opioids, paracetamol, combination therapy, and placebo with different routes for all outcomes.

The numbers of included studies for all outcomes is shown in Figures 1, **2** presents that the network of eligible studies with NSAIDs, opioids, paracetamol, combination therapy and placebo with different routes for pain variance at 30 min. Other outcomes are shown in Supplement Figures 5, **6** shows the results of loop consistency for all outcomes. Table 2 demonstrates the results of NSAIDs, opioids, paracetamol, combination therapy and placebo with different administration routes for pain variance at 30 min and failure of complete relief at 30 min. Supplement Tables 4, 5 indicate the results of network meta-analysis of NSAIDs, opioids, paracetamol, combination therapy and placebo with different administration routes for failure of ≥50% pain relief at 30 min, the need for rescue analgesia, nonspecific acute adverse events and vomiting as an adverse event.

**Table 2:** The results of network meta-analysis of NSAIDs, opioids, paracetamol, combination therapy and placebo with different routes for pain variance at 30 min and failure of complete relief at 30 min from second stage.

**NSAIDs, IM**	0.79 (0.10, 6.22)	0.76 (0.11, 5.79)	1.14 (0.14, 8.96)	1.25 (0.27, 5.94)	0.72 (0.12, 4.31)	0.22 (0.01, 4.60)	0.78 (0.06, 9.15)	–	2.00 (0.16, 30.59)	–	0.29 (0.06, 1.45)
6.92 (−1.62, 15.51)	**NSAIDs, IV**	0.97 (0.13, 7.71)	1.42 (0.07, 24.73)	1.57 (0.16, 15.35)	0.91 (0.33, 2.40)	0.28 (0.03, 2.60)	0.99 (0.16, 5.28)	–	2.57 (0.36, 17.38)	–	0.37 (0.04, 4.18)
−12.91 (−27.02, 1.46)	**−19.91 (−36.1, –3.21)**	**NSAIDs, PR**	1.45 (0.08, 23.55)	1.64 (0.26, 8.84)	0.94 (0.14, 5.35)	0.29 (0.01, 5.64)	1.01 (0.08, 11.81)	–	2.68 (0.19, 33.64)	–	0.38 (0.03, 3.83)
−9.55 (−26.09, 6.93)	−16.56 (−35.14, 2.73)	3.39 (−18.83, 24.54)	**NSAIDs, SU**	1.09 (0.08, 15.12)	0.63 (0.04, 10.38)	0.19 (0.01, 7.30)	0.68 (0.02, 17.95)	–	1.72 (0.07, 51.99)	–	0.26 (0.02, 2.78)
−1.98 (−8.60, 4.60)	−8.81 (−19.34, 1.87)	10.89 (−1.51, 23.89)	7.43 (−10.61, 25.31)	**Opioids, IM**	0.57 (0.08, 4.31)	0.18 (0.01, 3.74)	0.62 (0.03, 9.72)	–	1.62 (0.11, 27.03)	–	0.24 (0.03, 1.85)
**−7.27 (−13.40, –1.08)**	**−14.21 (−20.88, –7.76)**	5.66 (−9.88, 21.04)	2.42 (−15.76, 19.71)	−5.28 (−14.01, 3.64)	**Opioids, IV**	0.30 (0.03, 3.43)	1.10 (0.18, 6.01)	–	2.74 (0.44, 18.65)	–	0.41 (0.04, 3.91)
−1.50 (−19.24, 17.54)	−8.37 (−24.43, 8.51)	11.56 (−11.88, 35.20)	8.20 (−16.67, 32.81)	0.40 (−18.81, 20.20)	5.72 (−11.12, 23.55)	**Opioids, SC**	3.62 (0.20, 62.66)	–	9.61 (0.51, 167.8)	–	1.41 (0.06, 30.99)
−4.20 (−10.41, 1.73)	**−11.10 (−18.65, –4.24)**	8.76 (−6.58, 24.26)	5.28 (−12.70, 22.77)	−2.31 (−11.38, 6.63)	3.10 −2.19, 8.14	−2.58 (−21.32, 14.74)	**Paracetamol, IV**	–	2.68 (0.23, 33.14)	–	0.38 (0.03, 5.51)
−12.73 (−26.09, 0.25)	**−19.43 (−36.36, –4.13)**	0.21 (−18.60, 19.93)	−3.27 (−23.53, 18.1)	−10.55 (−25.87, 3.78)	−5.42 (−20.01, 8.92)	−11.21 (−33.76, 11.13)	−8.31 (−23.44, 5.80)	**Paracetamol, PO**	–	–	–
–	–	–	–	–	–	–	–	–	**NSAIDs, IV** **+** **Opioids, IV**	–	0.14 (0.01, 2.30)
**16.33 (1.62, 31.51)**	9.11 (−7.96, 26.96)	**29.04 (8.16, 49.93)**	**25.89 (3.57, 48.08)**	**18.23 (2.01, 34.72)**	**23.36 7.38, 39.90**	17.69 (−5.79, 42.15)	**20.55 (4.30, 36.72)**	**29.15 (14.34, 42.60)**	–	**NSAIDs, IM** **+** **Paracetamol, PO**	–
**−20.92 (−34.59, –7.24)**	**−27.78 −44.1, –10.86**	−7.93 (−28.23, 11.96)	−11.68 (−32.32, 10.5)	**−18.85 (−34.21, –3.45)**	−13.66 (−28.21, 1.89)	−19.52 (−41.87, 3.69)	**−16.56 (−31.44, –1.42)**	−8.12 (−27.31, 11.12)	–	**−37.07 (−57.05, –16.40)**	**Placebo**

*Comparisons between treatments should be read from left to right and the estimate is in the cell in common between the upper-left-defining treatment and the lower-right-defining treatment. For pain variance at 30 min from Stage II in the lower left corner, the mean difference (MD) lower than 0 favor the upper-left-defining treatment. For failure of complete relief at 30 min from Stage II in the upper right corner, the odds ratios (ORs) lower than 1 favor the upper-left-defining treatment. To obtain MDs for comparisons in the opposite direction, negative values should be converted into positive values, and vice versa. To obtain ORs for comparisons in the opposite direction, reciprocals should be taken. The consistency model with random effects was employed for pain variance at 30 min, while the design-by-treatment interaction model with random inconsistency effects was employed for failure of complete relief at 30 min (heterogeneity: 0.642, inconsistency: 0.508). Significant results are in bold and underlined, and “–” means that the results are not available. NSAIDs, Nonsteroidal anti-inflammatory drugs; IM, Intramuscular route; IV, Intravenous route; PO, Per oral route; PR, Per rectal route; SC, Subcutaneous route*.

That forest plots for effect sizes compared with NSAIDs via the IM route. NSAIDs via the IM route are superior to opioids via the IV route (MD: −7.27, 95% CrIs: −13.40 to −1.08) in pain variance at 30 min is shown in Supplement Figure 7. NSAIDs via the IM route required less rescue analgesia than opioids via the IV route (OR: 0.36, 95% CrIs: 0.15 to 0.77) and paracetamol via the IV route (OR: 0.36, 95% CrIs: 0.13 to 0.87). NSAIDs used with the IM route had fewer nonspecific adverse events than NSAIDs via the PR route (OR: 0.02, 95% CrIs: 0.00004 to 0.65), opioids via the IM route (OR: 0.22, 95% CrIs: 0.11 to 0.42), and opioids via the IV route (OR: 0.28, 95% CrIs: 0.10 to 0.74). Supplement Figure 7 also further confirms the results in the first stage, such as placebo vs. NSAIDs using the IM route for pain variance at 30 min (MD: −20.9, 95% CrIs: −34.6 to −7.24) and the need for rescue analgesia (OR: 0.15, 95% CrIs: 0.03 to 0.71). The results in Stage I for nonspecific acute adverse events are also consistent for opioids via the IM route vs. NSAIDs via the IM route and double opioids via the IM route vs. NSAIDs via the IM route.

**Figure 4:**
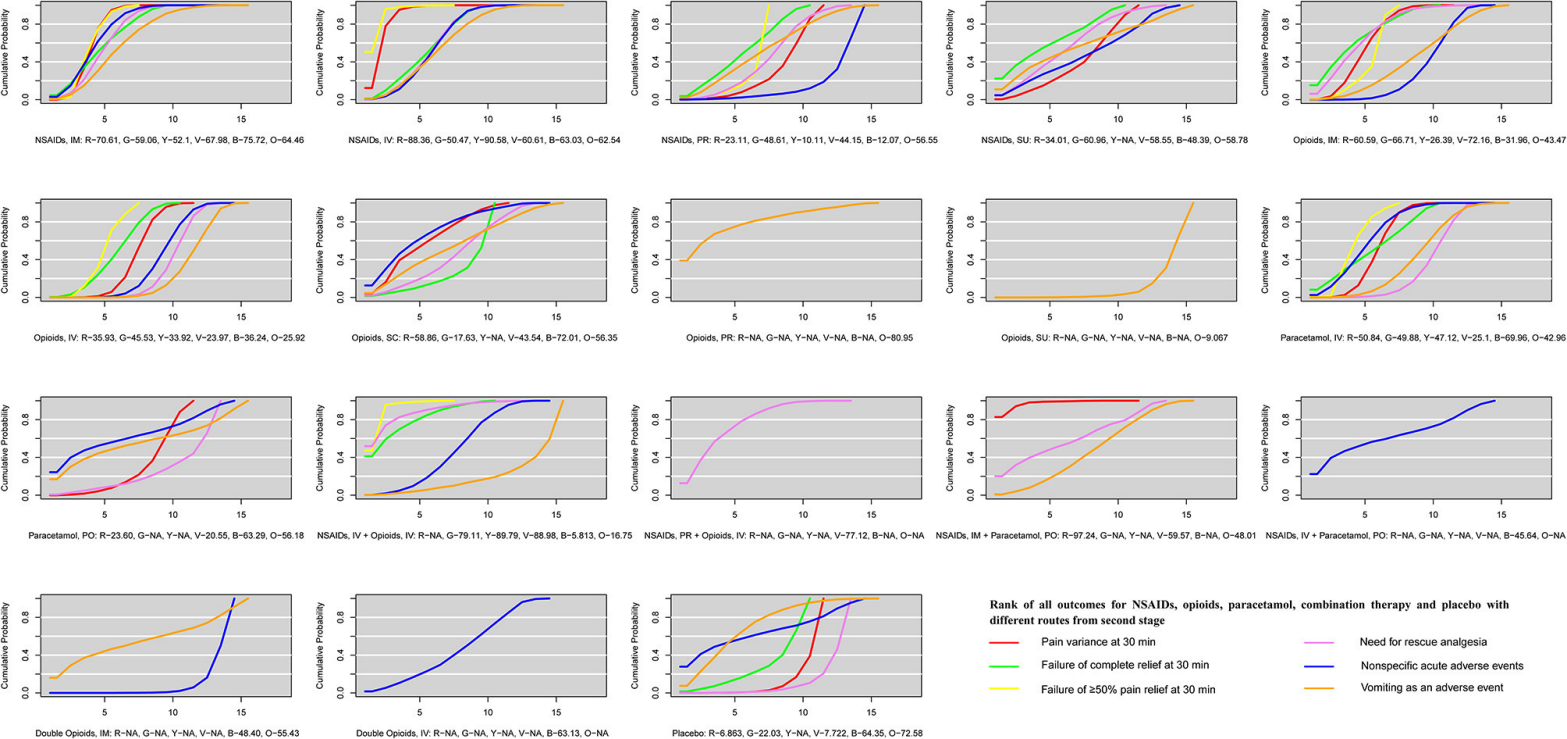
The ranking of NSAIDs, opioids, paracetamol, combination therapy and placebo with different routes for all outcomes from second stage. All co-linked active drugs and placebo for all outcomes were ranked according to their SUCRA values. In ranking order for all outcomes, from best to worst, the higher SUCRA scores demonstrate better effects or safer. R, Red, indicates pain variance at 30 min; G, Green, indicates failure of complete relief at 30 min; Y, Yellow, indicates failure of ≥50% pain relief at 30 min; V, Violet, indicates need for rescue analgesia; B, Blue, indicates nonspecific acute adverse events; O, Orange, indicates vomiting as an adverse event. SUCRA, Surface under the cumulative ranking; NSAIDs, Non-steroidal anti-inflammatory drugs; IM, Intramuscular route; IV, Intravenous route; PO, Per oral route; PR, Per rectal route; SC, Subcutaneous route; SU, Sublingual route.

The ranking of NSAIDs, opioids, paracetamol, combination therapy, and placebo with different routes is shown in Figure 4. Viewed as a whole, NSAID administration using the IV route or IM route is the optimal treatment in terms of drug classification and route of administration for all outcomes evaluating both effectiveness and safety. For pain variance at 30 min, combination therapy (NSAIDs via the IM route plus paracetamol via the PO route) ranked fist (97.24%) and NSAIDs via the IV route ranked second (88.36%). NSAIDs used with the IM route and opioids with the SC route had fewer nonspecific acute adverse events (75.72 and 72.01%, respectively). Opioids via the PR route and placebo had fewer vomiting events (80.95 and 72.58%). It is also clear that the IV route and IM routes are superior to PR and SU routes in treatment with NSAIDs for all outcomes. NSAIDs with the IV route had a rapid pain relief at 30 min. However, the IM route is superior to the IV route in adverse events. For vomiting events, opioids with the SC route or IM route were superior to the IV route. For paracetamol, the IV route is superior to the PO route.

That combination therapy (NSAIDs via the IM or IV route plus paracetamol via the PO route), NSAIDs via the IV route, and NSAIDs via the IM route are superior to other drugs with different administration routes for pain variance at 30 min and nonspecific acute adverse events and vomiting as an adverse event is shown in Supplement Figure 8. As is shown in Supplement Table 6, the results of sensitive analysis are stable after excluding studies with placebo and zero events, single-blinded and unblinded studies and dividing pain scores in two groups by VAS 100 mm and 10 cm scales. The comparison-adjusted funnel plots of NSAIDs, opioids, paracetamol, combination therapy and placebo with different routes for all outcomes are presented in Supplement Figure 9.

#### The Result of Network Meta-Analysis From Stage III

To determine the optimal drug and corresponding route, the analysis of different drug branches and routes of administration were performed. The numbers of included studies for outcomes are shown in Figure 1. For pain variance at 30 min, diclofenac used with the IM route plus paracetamol with the PO route, ibuprofen with the IV route and pethidine with the IM route ranked as the top three (Supplement Figure 10, Part 1). In addition, ketorolac via the IV route, ketorolac via the IM route and lornoxicam via the IV route ranked as the top three for pain variance at 30 min (Supplement Figure 10, Part 2). Similarly, other outcomes are shown in Supplement Figures 11–15. Supplement Figure 16 shows SUCRA of co-linked active drugs for pain variance at 30 min and nonspecific acute adverse events in network meta-analyses from the third stage. Ibuprofen used with the IV route, ketorolac with the IV route and diclofenac with the IM route ranked foremost using the SUCRA value from pain variance at 30 min and nonspecific acute adverse events.

## Discussion

Although acute renal colic is not a life-threatening disease, management of pain induced by kidney stones is worth pursuing from the point of view of humanistic care and high quality of life. Pain relief is the first therapeutic step for patients with an acute stone episode (Phillips et al., 2009). In this network meta-analysis, pain variance at 30 min was the primary outcome to assess drugs with rapid pain relief. Additionally, routes of administration overlooked or not clearly studied by clinicians or researchers were also factors influencing efficacy and safety (Pathan et al., 2018). Therefore, our network meta-analysis also discussed the routes of administration. Moreover, rankings (Jansen et al., 2014) of included interventions and specific drugs were carried out to help clinicians make the optimal decision for pain relief with fewer adverse events.

According to our three-stage study design, several findings deserve to be noted. More specifically, the efficacy and safety among NSAIDs, opioids, paracetamol, combination therapy, and placebo were compared according to the classification and pharmacological mechanism of these five interventions in the first stage. Our analysis confirmed that NSAIDs are superior to opioids and paracetamol, which is consistent with some previous studies (Cordell et al., 1996; Wood et al., 2000; Ay et al., 2014; Kaynar et al., 2015; Pathan et al., 2016). The European Association of Urology (EAU) guideline for urolithiasis in 2018 (EAU Guidelines Office) also recommended NSAIDs for very effective treatment of acute renal colic and found they were superior to opioids. The direct mechanism of NSAIDs resulting in inhibition of the cyclooxygenase enzyme could explain this superiority. In addition, although combination therapy achieved rapid pain relief, there were more adverse events compared with NSAIDs, which could be explained by the multiple mechanisms and combined adverse events of different drugs. It should be highlighted that combination therapy was also an optimal choice for patients with uncontrolled pain after receiving NASIDs. In addition, our analysis also found opioids induced more vomiting events and nonspecific adverse events, as has been reported in other studies (Holdgate and Pollock, 2005; Pathan et al., 2016). Then, our analysis compared five interventions based on routes of administration to figure out the impact of routes on treatment of acute renal colic in the second stage. Moreover, our analysis provided the ranking of five interventions with different routes for all outcomes. There are no specific routes of administration recommended for acute renal colic in the 2018 EAU guideline for urolithiasis (EAU Guidelines Office). In this network meta-analysis, NSAIDs via the IV route or IM route are the optimal choice compared with opioids, paracetamol, combination and placebo based on the scores of SUCRA. It is should be noted the scores of SUCRA might not be a clinical relevant difference between the top 1 and 2. From the perspective of efficacy, NSAIDs used with the IV route are superior to NSAIDs with the IM route. From a safety perspective, NSAIDs used with the IM route are superior to NSAIDs with the IV route. The interpretation of a recent RCT published in the Lancet by Pathan et al. (2016) that treatment with NSAIDs IM is the most effective for acute renal colic in the emergency department and incurs fewer adverse events, which seem to be inappropriate. Our findings in the second stage validate a recent study (Pathan et al., 2018) demonstrating that the benefit of NSAIDs compared with opioids was route specific to some extent and also enrich and perfect its results from network meta-analysis.

In the third stage, we compared these five interventions based on different drug branches and routes of administration through network meta-analysis. Because of the small studies assigned to interventions, global network plots of this stage were composed of natural punitive unconnecting small network plots. To determine the optimal treatment, we further explored efficacy and safety from the perspective of pain variance at 30 min and nonspecific adverse events, and found that diclofenac with the IM route was superior to morphine with the IV route. Pathan et al. (2016) reported that diclofenac with the IM route was superior to morphine with the IV route, which was consistent with the counterpart in our network meta-analysis. However, Pathan's conclusion (Pathan et al., 2016) is part of our findings in the third stage based on the ranking of five interventions with different routes. The three analgesic regimens, including ibuprofen via the IV route, ketorolac via the IV route and diclofenac via the IM route, are superior to other interventions. In our network analysis, diclofenac with the IM route is inferior to ibuprofen with the IV route, which could be explained by studies with ibuprofen IV and ketorolac IV being small and requiring more RCTs to investigate and confirm whether results of the third stage are correct based on current studies. In addition, it was reported that diclofenac and ibuprofen increased major coronary events, and diclofenac is contraindicated in patients with congestive heart failure (New York Heart Association class II-IV), ischaemic heart disease, peripheral arterial disease and cerebrovascular disease (Krum et al., 2012; Bhala et al., 2013) Additionally, the use of ketorolac is also cautioned against for patients who have risks of cardiovascular events (Physician's Desk Reference, 2017). Even so, we noted the superiority of this drug therapy in short-term management of pain relief. Moreover, the thorough treatment of refractory renal colic pain depends on the removal of stones (EAU Guidelines Office). Hence, our study recommends diclofenac used with the IM route with more reliable results for patients without risk of cardiovascular events. Otherwise, diclofenac IM could be acceptable after careful consideration or other NSAIDs could be chosen. Because there are only a few studies, the results of ibuprofen with the IV route and ketorolac with the IV route need to be verified. In fact, as more new drugs are developed and more RCTs carried out in the future, our results of network meta-analysis recommending diclofenac *via* the IM route as the optimal treatment may be challenged. However, it does show superiority currently. For patients, timely use of extracorporeal shock wave lithotripsy and the selection of reasonable lithotropic drugs are particularly important for the radical treatment of kidney stone (Yang et al., 2017).

There are several highlights in this network meta-analysis with a large sample, and multi-interventions at the highest level of evidence (Leucht et al., 2016) for treatment can be recommended for clinical guidelines by the World Health Organization (Kanters et al., 2016). Firstly, the three-step study design is the most important. To reveal the nature of the efficacy and safety of NSAIDs, opioids, paracetamol, combination therapy and placebo, we designed the three-stage strategy. From classification, pharmacological mechanism and routes of administration to specific drug branches with different routes, we gradually selected the optimal treatment at every stage. Moreover, the results from every stage confirm each other providing reliable findings. Secondly, compared with traditional meta-analysis, the network meta-analysis with multiple interventions on one clinical question could provide more informationes (Jansen et al., 2014). In addition, the rankings of different interventions for every outcome are provided to help clinicians make the best treatment choice for acute renal colic. Thirdly, no other systematic review and network meta-analysis focusing on the comparisons of efficacy and safety of specific drugs with specific routes for treatment of acute renal colic had been found. This network meta-analysis not only reinforces previous studies, but also updates, revises, and supplies exciting evidence. Although, our conclusions may be challenged due to insufficient samples in some interventions, our network meta-analysis provides the direction for subsequent studies, leading to a reduction of research costs and unnecessary waste of resources.

## Limitations

Our network meta-analysis also has several limitations. Firstly, the number of studies with the PO, SU, and SC route in the second stage is small. In the third stage, after naturally splitting interventions into specific drugs with different routes, the sample size and the number of RCTs included in each outcome became even smaller. Secondly, the number of RCTs with placebo is small, most likely due to ethical issues of using placebo to treat a patient with acute severe pain (Afshar et al., 2015) and there are also fewer RCTs with combination therapy. Thirdly, most trials in this network meta-analysis had unclear risks of bias, which could lead to errors (Buchberger et al., 2014; Faggion, 2015). Therefore, more high quality RCTs mainly investigating NSAIDs and paracetamol using different administration routes, especially, ibuprofen, ketorolac and the PR route need to be carried out to verify our findings.

## Conclusions

NSAIDs were found to be superior to opioids, paracetamol and placebo both in efficacy and safety for acute renal colic. Combination therapy reaps rapid pain relief at the cost of safety and NSAIDs with the IV route or IM route ranked first in all interventions with different routes regarding efficacy or safety, respectively. Moreover, these three analgesic regimens, including ibuprofen used with the IV route, ketorolac with the IV route and diclofenac with the IM route, were superior compared with other interventions based on the included studies in our network meta-analysis. Therefore, our study recommends NSAIDs with the IV or IM route for management of acute renal colic. Diclofenac via the IM route for more reliable results is recommended for patients without risks of cardiovascular events. Otherwise, diclofenac using the IM route could be acceptable after careful consideration or other NSAIDs could be chosen. Because of only a few studies, the results of ibuprofen with the IV route and ketorolac with the IV route need to be verified. Furthermore, Combination therapy is an alternative choice for uncontrolled pain after the use of NSAIDs.

## Author Contributions

Y-MN had full access to all data in the study and takes responsibility for the integrity of the data and the accuracy of data analysis. Y-MN, CZ Study concept and design. H-YG, JL, J-YW, and Q-SY Acquisition of data. H-YG and Y-MN Analysis and interpretation of data. H-YG and CZ Drafting of the manuscript. CZ and Y-MN Critical revision of the manuscript for important intellectual content. Y-MN, and H-YG Statistical analysis. Y-MN Administrative, technical, or material support. Y-MN and CZ Supervision.

### Conflict of Interest Statement

The authors declare that the research was conducted in the absence of any commercial or financial relationships that could be construed as a potential conflict of interest.
