# Supplementary material for: Increasing Nonsteroidal Anti-inflammatory Drugs and Reducing Opioids or Paracetamol in the Management of Acute Renal Colic: Based on Three-Stage Study Design of Network Meta-Analysis of Randomized Controlled Trials
Source: Front Pharmacol. 2019 Feb 22;10:96. doi: 10.3389/fphar.2019.00096 (PMC6395447; doi:10.3389/fphar.2019.00096)
Supplement: Supplementary file 1 [file Table_1.DOCX]

**Supplement Legends of Method, Table, and Figure**

**Supplement Method 1. Detailed search strategy.**

**Supplement Method 2.** **Reason from exclusion of literature.**

**Supplement Table 1.** **Summary of included clinical trials and patient characteristics.**

**Supplement Table 2. The results of network meta-analysis of NSAIDs, opioids, paracetamol, combination therapy and placebo for failure of ≥50% pain relief at 30 min and need for rescue analgesia from first stage.**

**Supplement Table 3.** **The results of network meta-analysis of NSAIDs, opioids, paracetamol, combination therapy and placebo for nonspecific acute adverse events and vomiting as an adverse event from first stage.**

**Supplement Table 4.** **The results of network meta-analysis of NSAIDs, opioids, paracetamol, combination therapy and placebo with different routes for failure of ≥50% pain relief at 30 min and need for rescue analgesia from second stage.**

**Supplement Table 5.** **The results of network meta-analysis of NSAIDs, opioids, paracetamol, combination therapy and placebo with different routes for nonspecific acute adverse events and vomiting as an adverse event from second stage.**

**Supplement Table 6. The sensitivity analyses results of network analysis from second stage.**

**Supplement Figure 1.** **The summary plot for risk of bias.**

**Supplement Figure 2.** **The network of eligible studies with different interventions for failure of complete relief at 30 min (A), failure of ≥50% pain relief at 30 min (B), need for rescue analgesia (C), nonspecific acute adverse events (D), and vomiting as an adverse event (E) from first stage.**

**Note:** The node sizes correspond to the number of accumulated sample size that investigated the treatments. Directly comparable treatments are linked with a line, and the thickness of the line corresponds to the sum of the sample size in each pairwise treatment comparison. NSAIDs: Nonsteroidal anti-inflammatory drugs.

**Supplement Figure 3. The results of loop consistency for pain variance at 30 min (A), failure of complete relief at 30 min (B), failure of ≥50% pain relief at 30 min (C), need for rescue analgesia (D), nonspecific acute adverse events (E), and vomiting as an adverse event (F) from first stage.**

**Supplement Figure 4. The results of direct comparison with NSAIDs, opioids, paracetamol, combination therapy and placebo with different routes for pain variance at 30 min (A), failure of complete relief at 30 min (B), failure of ≥50% pain relief at 30 min (C), need for rescue analgesia (D), nonspecific acute adverse events (E), and vomiting as an adverse event (F) from second stage.**

**Note:** NSAIDs: Nonsteroidal anti-inflammatory drugs, IM: Intramuscular route, IV: Intravenous route, PO: Per oral route, PR: Per rectal route, SC: Subcutaneous route, SU: Sublingual route.

**Supplement Figure 5.** **The network of eligible studies with NSAIDs, opioids, paracetamol, combination therapy and placebo with different routes for failure of complete relief at 30 min (A), failure of ≥50% pain relief at 30 min (B), need for rescue analgesia (C), nonspecific acute adverse events (D), and vomiting as an adverse event (E) from second stage.**

**Note:** The node sizes correspond to the number of trials that investigated the treatments. Directly comparable treatments are linked with a line, and the thickness of the line corresponds to the sum of the sample size in each pairwise treatment comparison. NSAIDs: Nonsteroidal anti-inflammatory drugs, IM: Intramuscular route, IV: Intravenous route, PO: Per oral route, PR: Per rectal route, SC: Subcutaneous route, SU: Sublingual route.

**Supplement Figure 6.** **The results of loop consistency for pain variance at 30 min (A), failure of complete relief at 30 min (B), failure of ≥50% pain relief at 30 min (C), need for rescue analgesia (D), nonspecific acute adverse events (E), and vomiting as an adverse event (F) from second stage.**

**Supplement Figure 7. Forest plots for effect sizes compared with NSAIDs with intramuscular route from second stage.**

**Note:** The result of “a” indicates the comparison between nonsteroidal anti-inflammatory drugs with intramuscular route (NSAIDs, IM) and nonsteroidal anti-inflammatory drugs with per rectal route plus opioids with intravenous route (NSAIDs, PR + Opioids, IV), and “b” indicates the comparison between nonsteroidal anti-inflammatory drugs with intramuscular route (NSAIDs, IM) and nonsteroidal anti-inflammatory drugs with intravenous rout plus paracetamol with per oral route (NSAIDs, IV + Paracetamol, PO). NSAIDs: Nonsteroidal anti-inflammatory drugs, IM: Intramuscular route, IV: Intravenous route, PO: Per oral route, PR: Per rectal route, SC: Subcutaneous route, SU: Sublingual route.

**Supplement Figure 8. Ranking for NSAIDs, opioids, paracetamol, combination therapy and placebo with different routes for pain variance at 30 min and nonspecific acute adverse events (A)/vomiting as an adverse event (B) in network meta-analyses from second stage.**

**Note:** All active drugs and placebo for all outcomes were ranked according to their probability of optimal efficacy or safety. In ranking order in the scatter plot, from best to worst, the higher order demonstrate better effects or safer. The best intervention is in the lower left corner, while the worst is in the upper right corner. Due to a lack of “NSAIDs, IM + Paracetamol, PO” in nonspecific acute adverse events, the ranking result of nonspecific acute adverse events in the part A, it were replaced by “NSAIDs, IV + Paracetamol, PO”. NSAIDs: Nonsteroidal anti-inflammatory drugs, IM: Intramuscular route, IV: Intravenous route, PO: Per oral route, PR: Per rectal route, SC: Subcutaneous route, SU: Sublingual route.

**Supplement Figure 9.** **The comparison-adjusted funnel plots with NSAIDs, opioids, paracetamol, combination therapy and placebo with different routes for pain variance at 30 min (A), failure of complete relief at 30 min (B), failure of ≥50% pain relief at 30 min (C), need for rescue analgesia (D), nonspecific acute adverse events (E), and vomiting as an adverse event (F) from second stage.**

**Note:** In the comparison-adjusted funnel plot the horizontal axis shows the difference of each i-study's estimate y_iXY from the summary effect for the respective cpomparison (y_iXY-mu_XY), while the vertical axis presents a measure of dispersion of y_iXY. In the absence of small-study effects all studies are expected to lie symmetrically around the zero line of the comparison-adjusted funnel plot. NSAIDs: Nonsteroidal anti-inflammatory drugs, IM: Intramuscular route, IV: Intravenous route, PO: Per oral route, PR: Per rectal route, SC: Subcutaneous route, SU: Sublingual route.

**Supplement Figure 10.** **Forest plots for effect sizes with different drug branches and routes for pain variance at 30 min from third stage.**

**Note:** IM: Intramuscular route, IV: Intravenous route, PO: Per oral route, PR: Per rectal route, SC: Subcutaneous route, SU: Sublingual route. DICL: Diclofenac, DIPY: Dipyrone, HYDR: Hydromorphine chloride-atropine, IBUP: Ibuprofen, INDM: Indomethacin, KETP: Ketoprofen, KETR: Ketorolac, LORN: Lornoxicam, MORP: Morphine, PARA: Paracetamol, PETH: Pethidine, PIRO: Piroxicam, TENO: Tenoxicam, TRAM: Tramadol.

**Supplement Figure 11.** **Integrated information plots with different drug branches and routes for failure of complete relief at 30 min from third stage.**

**Note:** The integrated information plot, including different drug branches and different routes, is composed of rude global network plot, natural punitive connecting network plots, forest plots, ranking, and their SUCRA values from network meta-analysis for failure of complete relief at 30 min in third stage. In rude global network plot, natural punitive connecting network plots, the node sizes correspond to the number of accumulated sample size that investigated the treatments. Directly comparable treatments are linked with a line, and the thickness of the line corresponds to the sum of the sample size in each pairwise treatment comparison. SUCRA: Surface under the cumulative ranking, IM: Intramuscular route, IV: Intravenous route, PR: Per rectal route, SC: Subcutaneous route, SU: Sublingual route. DICL: Diclofenac, FENT: Fentany, HYDR: Hydromorphine, INDM: Indomethacin, KETB: Ketogan, KETP: Ketoprofen, PARA: Paracetamol, PETH: Pethidine, PENT: pentazoxine, PIRO: Piroxicam.

**Supplement Figure 12.** **Integrated information plots with different drug branches and routes for failure of ≥50% pain relief at 30 min from third stage.**

**Note:** The integrated information plot, including different drug branches and different routes, is composed of rude global network plot, natural punitive connecting network plots, forest plots, ranking, and their SUCRA values from network meta-analysis for failure of ≥50% pain relief at 30 min in third stage. In rude global network plot, natural punitive connecting network plots, the node sizes correspond to the number of accumulated sample size that investigated the treatments. Directly comparable treatments are linked with a line, and the thickness of the line corresponds to the sum of the sample size in each pairwise treatment comparison. SUCRA: Surface under the cumulative ranking, IM: Intramuscular route, IV: Intravenous route, PR: Per rectal route. DICL: Diclofenac, KETR: Ketorolac, MORP: Morphine, PARA: Paracetamol, PETH: Pethidine, TRAM: Tramadol.

**Supplement Figure 13.** **Integrated information plots with different drug branches and routes for need for rescue analgesia from third stage.**

**Note:** The integrated information plot, including different drug branches and different routes, is composed of global network plot, forest plots, ranking, and their SUCRA values from network meta-analysis for need for rescue analgesia in third stage. In global network plot, the node sizes correspond to the number of accumulated sample size that investigated the treatments. Directly comparable treatments are linked with a line, and the thickness of the line corresponds to the sum of the sample size in each pairwise treatment comparison. SUCRA: Surface under the cumulative ranking, IM: Intramuscular route, IV: Intravenous route, PO: Per oral route, PR: Per rectal route, SC: Subcutaneous route, SU: Sublingual route. BUTO: Butorphanol, DICL: Diclofenac, DIPY: Dipyrone, HYDR: Hydromorphine, IBUP: Ibuprofen, INDM: Indomethacin, KETP: Ketoprofen, KETR: Ketorolac, LORN: Lornoxicam, MORP: Morphine, PAPA: Papaverine, PARA: Paracetamol, PARE: Parecoxib, PETH: Pethidine, PENT: Pentazoxine, PIRO: Piroxicam, TENO: Tenoxicam, TRAM: Tramadol.

**Supplement Figure 14.** **Integrated information plots with different drug branches and routes for nonspecific acute adverse events from third stage.**

**Note:** The integrated information plot, including different drug branches and different routes, is composed of rude global network plot, natural punitive connecting network plots, forest plots, ranking, and their SUCRA values from network meta-analysis for nonspecific acute adverse events in third stage. In rude global network plot, natural punitive connecting network plots, the node sizes correspond to the number of accumulated sample size that investigated the treatments. Directly comparable treatments are linked with a line, and the thickness of the line corresponds to the sum of the sample size in each pairwise treatment comparison. SUCRA: Surface under the cumulative ranking, IM: Intramuscular route, IV: Intravenous route, PO: Per oral route, PR: Per rectal route, SC: Subcutaneous route, SU: Sublingual route. BUPR: Buprenorphine, BUTO: Butorphanol, DEZO: Dezocine, DICL: Diclofenac, DIPY: Dipyrone, HYDR: Hydromorphine IBUP: Ibuprofen, INDM: Indomethacin, INDP: Indoprofen, KETB: ketobemidone, KETP: Ketoprofen, KETR: Ketorolac, LORN: Lornoxicam, MORP: Morphine, OXIC: Oxicone, OXYC: Oxyconchloride, PAPA: Papaverine, PARA: Paracetamol, PARE: Parecoxib, PETH: Pethidine, PIRO: Piroxicam, SPAS: Spasmofen, TEMG: Temgesic, TENO: Tenoxicam, TRAM: Tramadol.

**Supplement Figure 15. Integrated information plots with different drug branches and routes for vomiting as an adverse event from third stage.**

**Note:** The integrated information plot, including different drug branches and different routes, is composed of rude global network plot, natural punitive connecting network plots, forest plots, ranking, and their SUCRA values from network meta-analysis for vomiting as an adverse event in third stage. In rude global network plot, natural punitive connecting network plots, the node sizes correspond to the number of accumulated sample size that investigated the treatments. Directly comparable treatments are linked with a line, and the thickness of the line corresponds to the sum of the sample size in each pairwise treatment comparison. SUCRA: Surface under the cumulative ranking, IM: Intramuscular route, IV: Intravenous route, PO: Per oral route, PR: Per rectal route, SC: Subcutaneous route, SU: Sublingual route. BUPR: Buprenorphine, BUTO: Butorphanol, DEZO: Dezocine, DICL: Diclofenac, DIPY: Dipyrone, HYDO: Hydromorphone HYDR: Hydromorphine, IBUP: Ibuprofen, INDM: Indomethacin, INDP: Indoprofen, KETB: ketobemidone, KETP: Ketoprofen, KETR: Ketorolac, MORP: Morphine, OXIC: Oxicone, PAPA: Papaverine, PARA: Paracetamol, PARE: Parecoxib, PETH: Pethidine, PIRO: Piroxicam, SPAS: Spasmofen, TRAM: Tramadol.

**Supplement Figure 16. The SUCRA of co-linked active drugs for pain variance at 30 min and nonspecific acute adverse events in network meta-analyses from third stage.**

Note: All co-linked active drugs and placebo for all outcomes were ranked according to their SUCRA values. In the scatter plot, from best to worst, the higher SUCRA values demonstrate better effects or safer. The best intervention is in the upper right corner, while the worst is in the lower left corner. Red dot indicates the SUCRA value of Y-axis from pain variance at 30 min for part 1 of third stage, blue dot indicates the SUCRA value of Y-axis from pain variance at 30 min for part 2 of third stage. SUCRA: Surface under the cumulative ranking, IM: Intramuscular route, IV: Intravenous route, PO: Per oral route, PR: Per rectal route, SU: Sublingual route. DICL: Diclofenac, DIPY: Dipyrone, IBUP: Ibuprofen, KETP: Ketoprofen, KETR: Ketorolac, LORN: Lornoxicam, MORP: Morphine, PAPA: Papaverine, PARA: Paracetamol, PETH: Pethidine, PIRO: Piroxicam, TENO: Tenoxicam, TRAM: Tramadol.

**Supplement Method 1.** Detailed search strategy.

**Ovid MEDLINE(R) Epub Ahead of Print, In-Process & Other Non-Indexed Citations, Ovid MEDLINE(R) Daily, Ovid MEDLINE and Versions(R) <1946 to February 2, 2018>**

#1 exp Urinary Bladder Calculi/ or exp Urinary Calculi/ or exp Kidney Calculi/ or exp Ureteral Calculi/ or exp Urolithiasis/ or exp Nephrolithiasis/ or exp Renal Colic/ or exp Ureteral Diseases/ or exp Ureteral Obstruction/ or exp Kidney Diseases/ (496442)

#2 ((Urin* or renal or kidney or ureter* or bladder) adj3 (stone* or calcul* or colic* or lith* or obstruct* or occlusi*)).mp. / (80620)

#3 ((Kidney or ureter*) adj2 diseas*).mp. / (145253)

#4 (Urolith* or nephrolith*).mp. / (16184)

#5 or/1-4/ (537819)

#6 exp Anti-Inflammatory Agents, Non-Steroidal/ or exp Cyclooxygenase Inhibitors/ or exp Cyclooxygenase 2 Inhibitors/ (182543)

#7 ((Nonsteroidal adj2 antiinflammatory) or (Non-steroidal adj2 antiinflammatory) or (Nonsteroidal adj2 antiinflammatory) or (Non-steroidal adj2 antiinflammatory)).mp. / (5120)

#8 exp diclofenac/ or exp ketorolac/ or exp apazone/ or exp aspirin/ or exp ibuprofen/ or exp ketoprofen/ or exp Salicylates/ or exp etodolac/ or exp naproxen/ or exp indomethacin/ or exp piroxicam/ or exp celecoxib/ or exp fenoprofen/ or exp sulindac/ or exp tolmetin/ or exp mesalazine/ or exp aminosalicylic acid/ (119130)

#9 NSAID*.mp. / (22489)

#10 (Diclofenac or adiflam or agile or diclonac or dicol or diclonat* or feloran or voltarol or Voltaren or Cataflam or Voltaren-XR or Zipsor).mp. / (12408)

#11 (Aceclofenac or Hifenac or Cincofen or Nacsiv or Acenac).mp. / (379)

#12 (Ketorolac or toradol or torolac or kealc or kenalfin or ketlac).mp. / (2784)

#13 (Apazon* or Azapropazon* or cinnopropazon*).mp. / (260)

#14 (Aspirin* or acetylsal* or dispril or easprin* or salicylic*).mp. / (76477)

#15 (Ibuprofen or brufen or nuprin or rufen or salprofen or dolgit or salprofen or advil* or motrin or nurofen or actiprofen or addaprin or aktren or anadin or bugesic or ibuprox).mp. / (13144)

#16 (ketoprofen or orudis or oruvail or ketoflam or oruvail or fastum or ketonal or ketodol or knavon or actron or ketoprofeno).mp. / (3914)

#17 (Dexketoprofen or keral or enantyum or ketesgel or dolmen).mp. / (271)

#18 (Naproxen or naprosyn or naprosin or proxen or synflex or Aleve or Anaprox or Apronax or Naprelan).mp. / (6251)

#19 (etodolac or ramodar or ultradol or etova or dualgan or etodin or etopan or flancox or proxym or etodine or dolarit).mp. / (645)

#20 (Indomethacin* or indocid or indocin or indomet or indometacin or metindol or osmosin).mp. / (41784)

#21 (Piroxicam or feldene or dolocare or dolonex or ketolin).mp. / (3740)

#22 (Meloxicam or mobic or vivlodex).mp. / (1864)

#23 (Tenoxicam or mobiflex).mp. / (591)

#24 (celecoxib or celebrex or celebra).mp. / (6097)

#25 (rofecoxib or vioxx or ceoxx or ceeoxx).mp. / (2658)

#26 (valdecoxib or bextra).mp. / (524)

#27 (Nimesulid* or aulin or mesulid or nimalox or sulid* or sintalgin or nimsid* or nise or nimulid).mp. / (1718)

#28 (Meclofenamic or meclofenamat* or meclomen).mp. / (1480)

#29 (fenoprofen or fenopron).mp. / (477)

#30 (oxaprozin or oxaprozinum or daypro or dayrun or duraprox).mp. / (154)

#31 (sulindac or cinoril or imbaral).mp. / (1984)

#32 (tolmetin or tolectin).mp. / (1441)

#33 (flurbiprofen* or sulindac* or mesalazin* or sulfasalazin* or flufenamic* or tolmetin* or fenoprofen* or diflunisal* or niflumic* or ketorolac or trometamol* or parecoxib* or teriflunomid* or benoxaprofen* or suprofen* or fenbufen* or mebron* or mepirizole* or mepyrizole* or methopyrimazole* or Epirizolum* or Polihexanid* or Dalex* or Miton* or epirizol* or clonixin* or tolemetin* or nabumeton*).mp. / (21566)

#34 or/6-33/ (250573)

#35 exp Analgesics, opioid/ or exp alkaloids, opiate/ or exp narcotics/ (133496)

#36 opioid*.mp. / (100655)

#37 exp hydrocodone/ or exp dextropropoxyphene/ or exp fentanyl/ or exp meperidine/ or exp methadone/ or exp Morphine/ or exp Morphine Derivatives/ or exp oxymorphone/ or exp pentazocine/ or exp tramadol/ (32892)

#38 exp alfentanil/ or alfentanil*.mp. / (2346)

#39 exp codeine/ or codein*.mp. / (8503)

#40 (hydrocodon* or vicodin* or norco or lortab or zohydro).mp. / (1055)

#41 exp oxycodone/ or oxycodon*.mp. / (3146)

#42 (dextropropoxyphen* or darvon or darvocet or digesic or capadex).mp. / (1671)

#43 exp dihydromorphine/ or dihydromorphin*.mp. / (527)

#44 (fentanyl or actiq or duragesic or fentora or sublimaz* or fenta).mp. / (20494)

#45 (meperidin* or demerol or pethidin*).mp. / (7541)

#46 (methadon* or dolophin* or methadose or amidon* or symoron or physephton* or heptadon).mp. / (15400)

#47 (morphin* or oramorph or morphia or duramorph or contin or mscontin or sevredol or zomorphzomo).mp. / (58063)

#48 (oxymorphon* or numorphan or opana or morphon).mp. / (570)

#49 (pentazocin* or fortal or sosegon or talwin or fortwin or talacen).mp. / (3003)

#50 (tramadol or ultram).mp. / (4508)

#51 or/35-50/ (190669)

#52 exp Acetaminophen/ (16327)

#53 (abenol* or acamol* or acenol* or acephen* or acet suppositories* or acetalgin* or acetamino phenol* or acetaminophen* or acetaminophene* or acetaminophenol* or acetamol* or acetomenophen* or acetylaminophenol* or adorem* or afebrin* or algiafin* or algotropyl* or alphagesic* or alvedon* or amadil* or anacin 3* or anaflon* or analgiser* or apamide* or apirex* or arthralgen* or benuron* or biogesic* or calapol* or calodol* or dafalgan* or depyretin* or dirox* or dolex* or dolofen* or dolomol* or calpol* or eneril* or meforagesic* or dolorol* or metagesic* or napamol* or naprex* or pacemol* or pacimol* or duorol* or pamol* or panadol* or panamax* or panodil* or paracet* or paracetamole* or parageniol* or paragin* or paralen* or paralief* or paramax* or paramidol* or paximol* or paratabs* or perfalgan* or pyrigesic* or setamol* or tylenol* or tylex* or valadol* or winadol* or zydinol).mp. / (25254)

#54 or/52-53/ (25254)

#55 34 or 51 or 54/ (446874)

#56 randomized controlled trial.pt. / (452594)

#57 controlled clinical trial.pt. / (92123)

#58 (random$ or placebo$ or single blind$ or double blind$ or triple blind$).ti,ab. / (1043823)

#59 (retraction of publication or retracted publication).pt. / (11493)

#60 or/56-59/ (1211204)

#61 (animals not humans).sh. / (4387962)

#62 ((comment or editorial or meta-analysis or practiceguideline or review or letter or journal correspondence) not "randomized controlled trial").pt. / (3940409)

#63 (random sampl$ or random digit$ or random effect$ or random survey or random regression).ti,ab. not "randomized controlled trial".pt. / (70354)

#64 or/61-63/ (8175069)

#65 60 not 64/ (906925)

#66 5 and 55 and 65/ (730)

**Ovid EMbase <1947 to February 2, 2018>**

#1 exp kidney pain/ or exp kidney colic/ or exp bladder stone/ or exp ureter stone/ or exp urolithiasis/ or exp nephrolithiasis/ or exp ureter obstruction/ (70310)

#2 ((Urin* or renal or kidney or ureter* or bladder) adj3 (stone* or calcul* or colic* or lith* or obstruct* or occlusi*)).mp. / (81962)

#3 ((Kidney or ureter*) adj2 diseas*).mp. (202238)

#4 (Urolith* or nephrolith*).mp. / (52981)

#5 or/1-4/ (300947)

#6 exp nonsteroid antiinflammatory agent/ or exp cyclooxygenase 2 inhibitor/ or exp indometacin/ or exp piroxicam/ or exp acetylsalicylic acid/ or exp celecoxib/ or exp diclofenac/ or exp ibuprofen/ or exp naproxen/ or exp azapropazone/ or exp acetylsalicylic acid/ or exp ketoprofen/ or exp salicylic acid/ or exp ketorolac/ or exp ketoprofen/ or exp salicylic acid derivative/ or exp etodolac/ or exp fenoprofen/ or exp sulindac/ or exp tolmetin/ or exp mesalazine/ or exp aminosalicylic acid/ (699701)

#7 ((Nonsteroidal adj2 antiinflammatory) or (Non-steroidal adj2 antiinflammatory) or (Nonsteroidal adj2 anti-inflammatory)or (Non-steroidal adj2 anti-inflammatory)).mp. / (44143)

#8 NSAID*.mp. / (38916)

#9 (Diclofenac or adiflam or agile or diclonac or dicol or diclonat* or feloran or voltarol or Voltaren or Cataflam or Voltaren-XR or Zipsor).mp. / (38316)

#10 (Aceclofenac or Hifenac or Cincofen or Nacsiv or Acenac).mp. / (1557)

#11 (Ketorolac or toradol or torolac or kealc or kenalfin or ketlac).mp. / (10189)

#12 (Apazon* or Azapropazon* or cinnopropazon*).mp. / (1216)

#13 (Aspirin* or acetylsal or dispril or easprin* or salicylic*).mp. / (143163)

#14 (Ibuprofen or brufen or nuprin or rufen or salprofen or dolgit or salprofen or advil* or motrin or nurofen or actiprofen or addaprin or aktren or anadin or bugesic or ibuprox).mp. / (46239)

#15 (ketoprofen or orudis or oruvail or ketoflam or oruvail or fastum or ketonal or ketodol or knavon or actron or ketoprofeno).mp. / (12677)

#16 (Dexketoprofen or keral or enantyum or ketesgel or dolmen).mp. / (664)

#17 (Naproxen or naprosyn or naprosin or proxen or synflex or Aleve or Anaprox or Apronax or Naprelan).mp. / (25178)

#18 (etodolac or ramodar or ultradol or etova or dualgan or etodin or etopan or flancox or proxym or etodine or dolarit).mp. / (2614)

#19 (Indomethacin* or indocid or indocin or indomet or indometacin or metindol or osmosin).mp./(81516)

#20 (Piroxicam or feldene or dolocare or dolonex or ketolin).mp. / (11176)

#21 (Meloxicam or mobic or vivlodex).mp. / (5831)

#22 (Tenoxicam or mobiflex).mp. / (2003)

#23 (celecoxib or celebrex or celebra).mp. / (20130)

#24 (rofecoxib or vioxx or ceoxx or ceeoxx).mp. / (10315)

#25 (valdecoxib or bextra).mp. / (2604)

#26 (Nimesulid* or aulin or mesulid or nimalox or sulid* or sintalgin or nimsid* or nise or nimulid).mp. / (4609)

#27 (Meclofenamic or meclofenamat* or meclomen).mp. / (3271)

#28 (fenoprofen or fenopron).mp. / (2854)

#29 (oxaprozin or oxaprozinum or daypro or dayrun or duraprox).mp. / (715)

#30 (sulindac or cinoril or imbaral).mp. / (7362)

#31 (tolmetin or tolectin).mp. / (2671)

#32 (flurbiprofen* or sulindac* or mesalazin* or sulfasalazin* or flufenamic* or tolmetin* or fenoprofen* or diflunisal* or niflumic* or ketorolac or trometamol* or parecoxib* or teriflunomid* or benoxaprofen* or suprofen* or fenbufen* or mebron* or mepirizole* or mepyrizole* or methopyrimazole* or Epirizolum* or Polihexanid* or Dalex* or Miton* or epirizol* or clonixin* or tolemetin* or nabumeton*).mp. / (65396)

#33 or/6-32/ (735882)

#34 exp opiate/ or exp narcotic analgesic agent/ or exp opiate agonist/ (390803)

#35 exp hydrocodone/ or exp dextropropoxyphene/ or exp fentanyl/ or exp pethidine/ or exp methadone/ or exp oxymorphone/ or exp pentazocine/ or exp tramadol/ (121216)

#36 opioid*.mp. / (100255)

#37 exp alfentanil/ or alfentanil*.mp. / (6641)

#38 exp codeine/ or codein*.mp. / (21713)

#39 (hydrocodon* or vicodin* or norco or lortab or zohydro).mp. / (6136)

#40 exp oxycodone/ or oxycodon*.mp. / (15316)

#41 (dextropropoxyphen* or darvon or darvocet or digesic or capadex).mp. / (8062)

#42 exp dihydromorphine/ or dihydromorphin*.mp. / (995)

#43 (fentanyl or actiq or duragesic or fentora or sublimaz* or fenta).mp. / (60490)

#44 (meperidin* or demerol or pethidin*).mp. / (23249)

#45 (methadon* or dolophin* or methadose or amidon* or symoron or physephton* or heptadon).mp. / (31820)

#46 exp morphine/ or exp morphine derivative/ (184336)

#47 (morphin* or oramorph or morphia or duramorph or contin or mscontin or sevredol or zomorphzomo).mp. / (113563)

#48 (oxymorphon* or numorphan or opana or morphon).mp. / (2161)

#49 (pentazocin* or fortal or sosegon or talwin or fortwin or talacen).mp. / (9231)

#50 (tramadol or ultram).mp. / (18130)

#51 or/34-50/ (438005)

#52 exp paracetamol/ (79428)

#53 (abenol* or acamol* or acenol* or acephen* or acet suppositories* or acetalgin* or acetamino phenol* or acetaminophen* or acetaminophene* or acetaminophenol* or acetamol* or acetomenophen* or acetylaminophenol* or adorem* or afebrin* or algiafin* or algotropyl* or alphagesic* or alvedon* or amadil* or anacin 3* or anaflon* or analgiser* or apamide* or apirex* or arthralgen* or benuron* or biogesic* or calapol* or calodol* or dafalgan* or depyretin* or dirox* or dolex* or dolofen* or dolomol* or calpol* or eneril* or meforagesic* or dolorol* or metagesic* or napamol* or naprex* or pacemol* or pacimol* or duorol* or pamol* or panadol* or panamax* or panodil* or paracet* or paracetamole* or parageniol* or paragin* or paralen* or paralief* or paramax* or paramidol* or paximol* or paratabs* or perfalgan* or pyrigesic* or setamol* or tylenol* or tylex* or valadol* or winadol* or zydinol).mp. / (84847)

#54 or/52-53/ (84851)

#55 33 or 51 or 54/ (1151393)

#56 (random$ or placebo$ or single blind$ or double blind$ or triple blind$).ti,ab. / (1382820)

#57 RETRACTED ARTICLE/ (8744)

#58 or/56-57/ (1391291)

#59 (animal$ not human$).sh,hw. / (4335940)

#60 (book or conference paper or editorial or letter or review).pt. not exp randomized controlled trial/ (4587859)

#61 (random sampl$ or random digit$ or random effect$ or random survey or random regression).ti,ab. not exp randomized controlled trial/ (86887)

#62 or/59-61/ (8786761)

#63 58 not 62/ (1072570)

#64 5 and 55 and 63/ (1456)

**Cochrane Central Register of Controlled Trials** **in Cochrane Reviews <February 2, 2018>**

#1 ((kidney pain) or (kidney colic) or (bladder stone) or (ureter stone) or (urolithiasis) or (nephrolithiasis) or (ureter obstruction)):ti,ab,kw / (3778)

#2 ((NSAID) or (nonsteroid antiinflammatory agent) or (cyclooxygenase 2 inhibitor) or (indometacin) or (piroxicam) or (acetylsalicylic acid) or (celecoxib) or (diclofenac) or (ibuprofen) or (naproxen) or (azapropazone) or (acetylsalicylic acid) or (ketoprofen) or (salicylic acid) or (ketorolac) or (ketoprofen) or (salicylic acid derivative) or (etodolac) or (fenoprofen) or (sulindac) or (tolmetin) or (mesalazine) or (aminosalicylic acid)):ti,ab,kw / (24965)

#3 ((Opioids) or (opiate) or (narcotic analgesic agent) or (opiate agonist) or (hydrocodone) or (dextropropoxyphene) or (fentanyl) or (pethidine) or (methadone) or (oxymorphone) or (pentazocine) or (tramadol)):ti,ab,kw / (24104)

#4 (paracetamol or abenol* or acamol* or acenol* or acephen* or acet suppositories* or acetalgin* or acetamino phenol* or acetaminophen* or acetaminophene* or acetaminophenol* or acetamol* or acetomenophen* or acetylaminophenol* or adorem* or afebrin* or algiafin* or algotropyl* or alphagesic* or alvedon* or amadil* or anacin 3* or anaflon* or analgiser* or apamide* or apirex* or arthralgen* or benuron* or biogesic* or calapol* or calodol* or dafalgan* or depyretin* or dirox* or dolex* or dolofen* or dolomol* or calpol* or eneril* or meforagesic* or dolorol* or metagesic* or napamol* or naprex* or pacemol* or pacimol* or duorol* or pamol* or panadol* or panamax* or panodil* or paracet* or paracetamole* or parageniol* or paragin* or paralen* or paralief* or paramax* or paramidol* or paximol* or paratabs* or perfalgan* or pyrigesic* or setamol* or tylenol* or tylex* or valadol* or winadol* or zydinol):ti,ab,kw / (7179)

#5 #2 or #3 or #4 / (49139)

#6 #1 and #5 / (651)

**Clinical Trials Registry Platform for Clinicaltrials.gov, Interventional studies with results, <February 2, 2018>**

#1 renal colic / (4)

#2 kidney pain / (10)

#3 bladder stone / (1)

#4 ureter stone / (7)

#5 urolithiasis / (21)

#6 nephrolithiasis / (18)

#7 ureter obstruction / (1)

#8 or/1-7 /(29)

**WHO International Clinical Trials Registry Platform <February 2, 2018>**

((kidney pain) or (kidney colic) or (bladder stone) or (ureter stone) or (urolithiasis) or (nephrolithiasis) or (ureter obstruction)) / (36)

**Supplement Method 2.** **Reason from exclusion of literature.**

| **Study** | **Year** | **Title** | **Reasons** |
| --- | --- | --- | --- |
| Slade | 1967 | Clincal blind trial of three drugs in the control of renal colic. | The outcomes didn't meet the inclusion criteria. |
| Thybo | 1971 | Oxyphenbutazone therapy in ureteric calculus. | The outcomes didn't meet the inclusion criteria. |
| Wandschneider | 1973 | Oxyphenbutazone and its effect in urologic procedures. | The population didn't meet the inclusion criteria. |
| Nygrd | 1975 | Oxyphenbutazone in the treatment of acute ureteral stone disease. | The outcomes didn't meet the inclusion criteria. |
| Oosterlinck | 1976 | A double blind comparison between meptazinol (Wy 22811) and 'Buscopan' Compositum in renal colic. | The inventions didn't meet the meet the inclusion criteria. |
| Yuge | 1977 | The efficacy of oral pentazocine for colicky pain in patients with urinary stone: A double blind trial. | The inventions didn't meet the meet the inclusion criteria. |
| Holmlund | 1978 | Treatment of ureteral colic with intravenous indomethacin. | The outcomes didn't meet the inclusion criteria. |
| Finlay | 1981 | A prospective double blind comparison of buprenorphine and pethidine in the management of ureteric colic. | The outcomes didn't meet the inclusion criteria. |
| Oosterlinck | 1982 | An open comparative trial of three doses of ciramadol used intravenously in renal colic. | The inventions didn't meet the meet the inclusion criteria. |
| Torchi | 1983 | Intravenous indoprofen in the management of renal colic. | The outcomes didn't meet the inclusion criteria. |
| Pourrat | 1984 | Treatment of renal colic with ketoprofen. | The inventions didn't meet the meet the inclusion criteria. |
| Grenabo | 1984 | Indomethacin as Prophylaxis Against Recurrent Ureteral Colic. | The outcomes didn't meet the inclusion criteria. |
| Magrini | 1984 | Intravenous ketoprofen in renal colic: a placebo-controlled pilot study. | The outcomes didn't meet the inclusion criteria. |
| Pourrat | 1984 | Treatment of renal colic with ketoprofen. | This is a duplicate. |
| Broggini | 1985 | Diclofenac sodico nella colica ureterale. Studio di confronto in doppia cecita con placebo. | The outcomes didn't meet the inclusion criteria. |
| Broggini | 1985 | Sodium diclofenac in ureteral colic. Comparative double-blind study with placebo. | The outcomes didn't meet the inclusion criteria. |
| Lupi | 1986 | The treatment of ureteral colic with intramuscular injection of pirprofen: A double-blind comparison trial with indomethacin. | The outcomes didn't meet the inclusion criteria. |
| Henry | 1987 | Comparison of butorphanol tartrate and meperidine in moderate to severe renal colic. | The outcomes didn't meet the inclusion criteria. |
| Kromann-Andersen | 1987 | Acute pain due to kidney/ureter stones treated with intramuscular Voltaren or Ketogan. | The population didn't meet the inclusion criteria. |
| Nelson | 1988 | Rectal v. intravenous administration of indomethacin in the treatment of renal colic. | The outcomes didn't meet the inclusion criteria. |
| Henry | 1988 | Urological applications of butorphanol tartrate: postoperative pain and renal colic. | The population didn't meet the inclusion criteria. |
| Primus | 1989 | Tramadol versus metimazole in alleviating pain in ureteral colic. | The inventions didn't meet the meet the inclusion criteria. |
| Sala-Mateus | 1989 | Intravenous diclofenac sodium in the treatment of nephritic colic. | The inventions didn't meet the meet the inclusion criteria. |
| Sanahuja | 1990 | Intramuscular diclofenac sodium versus intravenous Baralgin in the treatment of renal colic. | The inventions didn't meet the meet the inclusion criteria. |
| No author | 1991 | Comparative study of the efficacy of dipyrone, diclofenac sodium and pethidine in acute renal colic. Collaborative Group of the Spanish Society of Clinical Pharmacology. | The outcomes didn't meet the inclusion criteria. |
| Mora | 1992 | Flurbiprofen: therapeutic alternative in nephritic colic. | The outcomes didn't meet the inclusion criteria. |
| Muriel | 1993 | Efficacy of two different intramuscular doses of dipyrone in acute renal colic. Cooperative Study Group. | The outcomes didn't meet the inclusion criteria. |
| Muriel | 1993 | Efficacy of two different intramuscular doses of dipyrone in acute renal colic. | This is a duplicate. |
| Villoria | 1995 | Comparison of the onset and duration of the analgesic effect of dipyrone, I or 2 g, by the intramuscular or intravenous route,in acute renal colic. | The outcomes didn't meet the inclusion criteria. |
| Laerum | 1995 | Oral diclofenac in the prophylactic treatment of recurrent renal colic. A double-blind comparison with placebo. | The outcomes didn't meet the inclusion criteria. |
| Laerum | 1995 | Diclofenac in the short-term prevention of recurrent colic from ureteral calculi. A placebo controlled double-blind study. | The outcomes didn't meet the inclusion criteria. |
| Ginifer | 1996 | Administration of rectal indomethacin does not reduce the requirement for intravenous narcotic analgesia in acute renal colic. | The outcomes didn't meet the inclusion criteria. |
| Laerum | 1996 | Intramuscular diclofenac versus intravenous indomethacin in the treatment of acute renal colic. | The outcomes didn't meet the inclusion criteria. |
| Bergus | 1996 | Pain relief for renal colic. | The population didn't meet the inclusion criteria. |
| Al-Waili | 1999 | Intramuscular piroxicam versus intramuscular diclofenac sodium in the treatment of acute renal colic: double-blind study. | The outcomes didn't meet the inclusion criteria. |
| Pellegrino | 1999 | Comparison of lysine clonixinate 200 mg versus diclofenac 75 mg in the treatment of renal colic pain. Prospective double-blind clinical trial in parallel groups. | The outcomes didn't meet the inclusion criteria. |
| Chaudhary | 1999 | Double blind, randomised, parallel, prospective, comparative, clinical evaluation of a combination of antispasmodic analgesic Diclofenac + Pitofenone + Fenpiverinium (Manyana(TM)) vs Analgin + Pitofenone + Fenpiverinium (Baralgan) in biliary, ureteric and intestinal colic. | The population didn't meet the inclusion criteria. |
| Bilora | 2000 | Use of piroxicam sublingually in acute ureteral colic. Comparison with diclofenac. | The outcomes didn't meet the inclusion criteria. |
| Chang | 2002 | Effectiveness of sublingual buprenorphine and intramuscular pethidine in acute renal colic. | The outcomes didn't meet the inclusion criteria. |
| Pavlik | 2004 | Comparison of Cizolirtine Citrate and Metamizol Sodium in the Treatment of Adult Acute Renal Colic: A Randomized, Double-Blind, Clinical Pilot Study. | The inventions didn't meet the meet the inclusion criteria. |
| Holdgate | 2004 | Nonsteroidal anti-inflammatory drugs (NSAIDS) versus opioids for acute renal colic. | The study design didn't meet the inclusion criteria. |
| Engeler | 2005 | A double-blind, placebo controlled comparison of the morphine sparing effect of oral rofecoxib and dicolofenac for acute renal colic. | The outcomes didn't meet the inclusion criteria. |
| Mortelmans | 2006 | Use of Tramadol Drip in Controlling Renal Colic Pain. | The inventions didn't meet the meet the inclusion criteria. |
| Altay | 2007 | Double-Blind, Placebo-Controlled, Randomized Clinical Trial of Sublingual or Intramuscular Piroxicam in the Treatment of Renal Colic A Comparative Study. | The outcomes didn't meet the inclusion criteria. |
| Pfizer | 2007 | Efficacy And Safety Of Parecoxib 40mg vs. Ketoprofen 100mg In The Management Of Acute Renal Colic. | The outcomes didn't meet the inclusion criteria. |
| Phillips | 2009 | Celecoxib in the Management of Acute Renal Colic: A Randomized Controlled Clinical Trial. | The outcomes didn't meet the inclusion criteria. |
| Sallami | 2009 | Treatment of acute renal colics by isoptine versus ketoprofen in monotherapy and in association: Double blind, randomized study on 100 patients. | The population didn't meet the inclusion criteria. |
| Hazhir | 2010 | Comparison of Intranasal Desmopressin and Intramuscular Tramadol Versus Pethidine in Patients With Renal Colic. | The inventions didn't meet the meet the inclusion criteria. |
| Tokgoz | 2010 | Comparison of the Analgesic Effects of Dexketoprofen and Diclofenac During Shockwave Lithotripsy. | The population didn't meet the inclusion criteria. |
| Asgari | 2011 | Treatment of renal colic with papaverine hydrochloride: a prospective double blind randomized study. | The outcomes didn't meet the inclusion criteria. |
| Aganovic | 2012 | Clinical Decision Making in Renal Pain Management. | The outcomes didn't meet the inclusion criteria. |
| Borys | 2014 | Comparison of the analgesic effects of dexketoprofen and diclofenac for the treatment of acute renal colic. | The outcomes didn't meet the inclusion criteria. |
| Sharma | 2014 | Re: Intracutaneous sterile water injection versus oral paracetamol for renal colic during pregnancy: a randomized controlled trial.. | The study design didn't meet the inclusion criteria. |
| Afshar | 2015 | Nonsteroidal anti-inflammatory drugs (NSAIDs) and nonopioids for acute renal colic. | The study design didn't meet the inclusion criteria. |
| Sanahuja | 2016 | Intramuscular dicwfenac sodium versus intravenous baralgin in the treatment of renal colic. | The outcomes didn't meet the inclusion criteria. |
| Hosseininejad | 2016 | Comparing the effects of sublingual buprenorphine and intravenous morphine on acute renal colic pain. | The outcomes didn't meet the inclusion criteria. |
| Pathan | 2016 | A double blind multi-arm randomized trial for efficacy of intramuscular diclofenac versus intravenous morphine versus intravenous paracetamol, in renal colic emergency department pain management. | The outcomes didn't meet the inclusion criteria. |
| Ziapor | 2017 | Comparison of Effect of Morphine-Chlorpheniramine Combined Versus Morphine Alone in Alleviating Acute Renal Colic Pain: A Randomized Clinical Trail. | The outcomes didn't meet the inclusion criteria. |

**Supplement Table 1.** **Summary of included clinical trials and patient characteristics.**

| **Study** | **Year** | **Country** | **Sample** | **Gender**  **(M:F)** | **Mean Age** | **Groups** | **Outcomes** | **Comparisons** |
| --- | --- | --- | --- | --- | --- | --- | --- | --- |
| Al[^1^](#_ENREF_1) | 2017 | Turkey | 300 | 78:22 71:29 67:33 | NA | Group 1: Ketoprofen 50mg IV Group 2: Fentanyl 2μg/kg IV Group 3: Paracetamol 10mg IV | Complete pain relief at 30 min, Need for rescue analgesia | NSAIDs, Opioids with Paracetamol |
| Hosseininejad[^2^](#_ENREF_2) | 2017 | Iran | 300 | 69:31 72:28 67:33 | 29.66 28.81 30.28 | Group 1: Ketorolac 30mg IV Group 2: Morphine 0.1mg/kg IV Group 3: Ketorolac 30mg IV+0.1mg/kg morphine IV | Adverse events | NSAIDs, Opioids with NSAIDS + Opioids |
| Mozafari[^3^](#_ENREF_3) | 2017 | Iran | 63 | 27:4 25:7 | 35.58 39.18 | Group1: Ketorolac tromethamine 30mg IV Group 2: Buprenorphine 2mg SU | Adverse events | NSAIDs with Opioids |
| Cenker[^4^](#_ENREF_4) | 2017 | Turkey | 200 | 129:71 | 36 | Group 1: Ibuprofen 800mg IV Group 2: Paracetamol 1g IV | Pain score (VAS 100mm) at 30 min,Need for rescue analgesia,Adverse events | NSAIDs with Paracetamol |
| Pathan[^5^](#_ENREF_5) | 2016 | Qatar | 1096 | 460:87 456:93 446:102 | 35.1 34.7 34.4 | Group 1: Diclofenac 75mg IM Group 2: Morphine 0.1mg/kg IV (based on measured weight) Group 3: Acetaminophen 1g IV | Pain score (NRS-11) at 30 min, 50% reduction in initial pain at 30 min, Need for rescue analgesia, Adverse events | NSAIDs,Opioids, with Paracetamol |
| Zamanian[^6^](#_ENREF_6) | 2016 | Iran | 158 | 52:27 50:29 | 37.3 37.2 | Group 1: Indomethacin 100mg PR Group 2: Morphine 10mg PR | Adverse events | NSAIDs with Opioids |
| Kaynar[^7^](#_ENREF_7) | 2015 | Turkey | 80 | 26:14 22:18 | 37.98 46.3 | Group 1: Diclofenac 75mg IM Group 2: Acetaminophen 1g IV | Pain score (VAS 10 cm) at 30 min, Adverse events | NSAIDs with Paracetamol |
| Shirazi[^8^](#_ENREF_8) | 2015 | Iran | 80 | 22:18 23:17 | 36.7 39.1 | Group 1: Indomethacin 100mg PR Group 2: Tramadol 50mg IM | Pain score (VAS 10 cm) at 30 min, Complete pain relief at 30 min, Need for rescue analgesia | NSAIDs with Opioids |
| Hosseini[^9^](#_ENREF_9) | 2015 | Iran | 541 | 351:190 | NA | Group 1: Diclofenac 100mg PR Group 2: Pethidine 50mg IM | 50% reduction in initial pain at 30 min | NSAIDs with Opioids |
| KandaSwamy[^10^](#_ENREF_10) | 2015 | UK | 100 | 39:11 42:8 | 33.86 37.02 | Group 1: Diclofenac 75 mg IM Group 2: Piroxicam 40 mg SU | Complete pain relief at 30min, Need for rescue analgesia | NSAIDs with NSAIDs |
| Ay[^11^](#_ENREF_11) | 2014 | Turkey | 52 | NA | NA | Group 1: Ketoprofen 50mg IV Group 2: Meperidine HCl 100mg IV | Pain score (NRS-11) at 30 min, Need for rescue analgesia, Adverse events | NSAIDs with Opioids |
| Masoumi[^12^](#_ENREF_12) | 2014 | Iran | 108 | 39:15 43:11 | 34.96 36.07 | Group 1: Morphine 0.1mg/kg IV Group 2: Acetaminophen 1g IV | Pain score (VAS 10cm) at 30 min, Adverse events | Opioids with Paracetamol |
| Payandemehr[^13^](#_ENREF_13) | 2014 | Iran | 69 | 33:4 29:3 | 35 31 | Group 1: Buprenorphine 2mg SU Group 2: Morphine 0.1mg/kg IV | Adverse events | Opioids with Opioids |
| Azizkhani[^14^](#_ENREF_14) | 2013 | Iran | 124 | 42:62 42:62 | 39.73 38.40 | Group 1: Morphine 0.1mg/kg IV Group 2: Acetaminophen 15mg/kg IV | Pain score (VAS 10cm) at 30 min, Adverse events | Opioids with Paracetamol |
| Narci[^15^](#_ENREF_15) | 2012 | Turkey | 50 | 13:12 14:11 15:10 | 39.6 35.8 34 | Group 1: Diclofenac 75mg IM Group 2: Acetaminophen 1g PO Group 3: Diclofenac 75mg IM+Acetaminophen 1g PO | Pain score (VAS 100 mm) at 30 min, Need for rescue analgesia, Adverse events | NSAIDs, Paracetamol with NSAIDs + Paracetamol |
| Asgari[^16^](#_ENREF_16) | 2012 | Iran | 550 | 172:103 163:112 | 36.52 35.94 | Group 1: Diclofenac 100mg PR +Placebo Group 2: Diclofenac 100mg PR+Papaverine hydrochloride IV | Adverse events | NSAIDs with NSAIDs + Opioids |
| Cevik[^17^](#_ENREF_17) | 2012 | Turkey | 123 | 21:20 24:17 30:9 | 38 38 34 | Group 1: Tenoxicam 20mg IV Group 2: Lornoxicam 8mg IV Group 3: Ketoprofen 50mg IV | Pain score (VAS 100mm) at 30 min, Need for rescue analgesia, Adverse events | NSAIDs with NSAIDs |
| Serinken[^18^](#_ENREF_18) | 2012 | Turkey | 73 | 23:12 28:10 | 31.3 29.1 | Group 1: Morphine 0.1mg/kg IV Group 2: Paracetamol 1g IV | Pain score (VAS 10cm) at 30 min, Need for rescue analgesia, Adverse events | Opioids with Paracetamol |
| Salameh[^19^](#_ENREF_19) | 2011 | Israel | 97 | 38:10 35:14 | 37 37 | Group 1: Diclofenac 75mg IM Group 2: Tramadol 100mg IM | Pain score (VAS 10 cm) at 30 min, Need for rescue analgesia | NSAIDs with Opioids |
| Grissa[^20^](#_ENREF_20) | 2011 | Tunisia | 100 | 21:29 20:30 | 40 39 | Group 1: Piroxicam 20mg IM Group 2: Paracetamol 1g IV | Pain score (VAS 100 mm) at 30 min, Adverse events | NSAIDs with Paracetamol |
| Glina[^21^](#_ENREF_21) | 2011 | Brazil | 338 | 110:64 103:61 | 64 61 | Group 1: Parecoxib 40 mg+Placebo IV Group 2: Ketoprofen 100 mg+Placebo IV | Pain score (VAS 100mm) at 30 min, Need for rescue analgesia, Adverse events | NSAIDs with NSAIDs |
| Bektas[^22^](#_ENREF_22) | 2009 | Turkey | 146 | 27:22 31:15 32:19 | 39 35 36 | Group 1: Morphine 0.1mg/kg IV (based on measured weight) Group 2: Paracetamol 1g IV Group 3: Placebo | Need for rescue analgesia, Adverse events | Opioids, Paracetamol with placebo |
| Snir[^23^](#_ENREF_23) | 2008 | Israel | 59 | 26:4 22:7 | 44.1 46.2 | Group 1: Diclofenac 75mg IM Group 2: Papaverine 120mg IV | Need for rescue analgesia, Adverse events | NSAIDs with Opioids |
| Yencilek[^24^](#_ENREF_24) | 2008 | Turkey | 73 | 28:9 27:9 | 39.9 40.6 | Group 1: Papaverine HCl 60 mg IV Group 2: Pethidine 50mg IV | Adverse events | Opioids with Opioids |
| Carpena[^25^](#_ENREF_25) | 2007 | Spain | 308 | 61:40 68:36 65:38 | 37.6 39.9 39.1 | Group 1: Ketoprofen 25mg IV Group 2: Ketoprofen 50mg IV Group 3: Dipyrone 2g IV | Need for rescue analgesia, Adverse events | NSAIDs with NSAIDs |
| Safdar[^26^](#_ENREF_26) | 2006 | USA | 86 | 57:29 | 38 | Group 1: Ketorolac 15mg IV Group 2: Morphine 5mg IV Group 3: Ketorolac 15mg IV+Morphine 5mg IV | Complete pain relief at 30min, Need for rescue analgesia, Adverse events | NSAIDs, Opioids with NSAIDs + Opioids |
| Altay[^27^](#_ENREF_27) | 2006 | Turkey | 80 | 24:15 25:16 | 39.2:42.2 42.6:38.3 | Group 1: Piroxicam 20mg SU Group 2: Piroxicam 40mg IM | Need for rescue analgesia, Adverse events | NSAIDs with NSAIDs |
| Carpena[^28^](#_ENREF_28) | 2003 | Spain | 333 | 70:42 70:43 68:40 | 42.1 41.7 39.7 | Group 1: Ketoprofen 25mg IM Group 2: Ketoprofen 50mg IM Group 3: Dipyrone 2g IM | Adverse events | NSAIDs with NSAIDs |
| Eray[^29^](#_ENREF_29) | 2002 | Turkey | 47 | 16:7 18:6 | 39 41 | Group 1: Tramadol 50 mg IV Group 2: Meperidine 50mg IV | Pain score (VAS 100mm) at 30 min, Need for rescue analgesia, Adverse events | Opioids with Opioids |
| Wood[^30^](#_ENREF_30) | 2000 | Canada | 142 | 57:8 68:9 | 42 40 | Group 1: Ketorolac 30mg IM Group 2: Meperidine 50mg IV + Dimenhydrinate 50mg | Pain score (VAS 100 mm) at 30 min | NSAIDs with Opioids |
| Larkin[^31^](#_ENREF_31) | 1999 | USA | 70 | 26:7 27:10 | 45.5 40.7 | Group 1: Ketorolac 60mg IM Group 2: Pethidine 100-150mg | Need for rescue analgesia, Adverse events | NSAIDs with Opioids |
| Torralba[^32^](#_ENREF_32) | 1999 | Spain | 48 | NA | NA | Group 1: Ketorolac 30mg IM Group 2: Tramadol 1mg/kg SC | Need for rescue analgesia, Adverse events | NSAIDs with Opioids |
| Cohen[^33^](#_ENREF_33) | 1998 | Israel | 57 | 24:3 23:7 | 44 42.4 | Group 1: Ketorolac 30mg IM Group 2: Diclofenac 75mg IM | Need for rescue analgesia | NSAIDs with NSAIDs |
| Supervia[^34^](#_ENREF_34) | 1998 | Spain | 80 | 23:27 31:9 | 36.5 41.5 | Group 1: Piroxicam 20mg SU Group 2: Diclofenac sodium 75mg IM | pain score (VAS 10cm) at 30 min, Need for rescue analgesia, Adverse events | NSAIDs with NSAIDs |
| Cordell[^35^](#_ENREF_35) | 1996 | USA | 106 | 30:6 28:7 25:10 | 38.8 42 36.1 | Group 1: Ketorolac 60mg IV Group 2: Meperidine 50mg IV Group 3: Ketorolac 60mg IV+Meperidine 50mg IV | Pain score (VAS 100 mm) at 30 min, 50% reduction in initial pain at 30 min, Need for rescue analgesia, Adverse events | NSAIDs, Opioids with NSAIDs + Opioids |
| Al-Sahlawi[^36^](#_ENREF_36) | 1996 | Kuwait | 100 | 34:16 37:13 30:19 | NA | Group 1: Indomethacin 100mg IV Group 2: Pethidine 100mg IV | Complete pain relief at 30min, Need for rescue analgesia, Adverse events | NSAIDs with Opioids |
| Stein[^37^](#_ENREF_37) | 1996 | Israel | 57 | 24:3 21:9 | 39.1 41.4 | Group 1: Ketorolac 60mg IM Group 2: Diclofenac 75mg IM | Need for rescue analgesia,Adverse events | NSAIDs with NSAIDs |
| Curry[^38^](#_ENREF_38) | 1995 | New Zealand | 41 | NA | NA | Group 1: Tenoxicam 40mg IV Group 2: Pethidine 75mg IV | Pain score (VAS 100 mm) at 30 min, Need for rescue analgesia, Adverse events | NSAIDs with Opioids |
| Cordell[^39^](#_ENREF_39) | 1994 | USA | 51 | 18:13 18:2 | 38.9 39.2 | Group 1: Indomethacin 100mg PR Group 2: Morphine 5-10mg IV | Adverse events | NSAIDs with Opioids |
| Sandhu[^40^](#_ENREF_40) | 1994 | UK | 154 | 59:17 58:20 | 45.2 42.1 | Group 1: Ketorolac 30mg IM Group 2: Pethidine 100mg IM | Adverse events | NSAIDs with Opioids |
| Jasani[^41^](#_ENREF_41) | 1994 | USA | 73 | 27:9 29:8 | 35.7 37.5 | Group 1: Hydromorphone 1mg IV Group 2: Meperidine 50mg IV | Adverse events | Opioids with Opioids |
| Walden[^42^](#_ENREF_42) | 1993 | Sweden | 86 | 60:26 | NA | Group 1: Ketoprofen 100mg IM Group 2: Diclofenac 50mg IM | Need for rescue analgesia, Adverse events | NSAIDs with NSAIDs |
| Arnau[^43^](#_ENREF_43) | 1991 | Spain | 234 | 63:53 61:57 67:49 57:44 | 40.7 41.4 41.2 42.9 | Group 1: Diclofenac 75mg IM Group 2: Pethidine 100mg IM Group 3: Dipyrone 1g IM Group 4: Dipyrone 2g IM | Pain score (VAS 10 cm) at 30 min, Need for rescue analgesia, Adverse events | NSAIDs with Opioids |
| Marthak[^44^](#_ENREF_44) | 1991 | India | 50 | 8:17 5:20 | 36.4 34 | Group 1: Diclofenac 75mg IM Group 2: Pethidine 75mg IM | Pain score (VAS 100 mm) at 30 min, Complete pain relief at 30 min, Adverse events | NSAIDs with Opioids |
| Oosterlinck[^45^](#_ENREF_45) | 1990 | UK | 125 | 32:13 29:8 28:10 | 40 41 39 | Group 1: Ketorolac 10mg IM Group 2: Ketorolac 90mg IM Group 3: Pethidine 100mg IM | Pain score (VAS 100 mm) at 30 min, Complete pain relief at 30 min, Adverse events | NSAIDs with Opioids |
| Gonzalez[^46^](#_ENREF_46) | 1990 | Spain | 40 | NA | NA | Group 1: Diclofenac 75mg IM Group 2: Buprenorphine 0.3mg IM | Adverse events | NSAIDs with Opioids |
| Indudhara[^47^](#_ENREF_47) | 1990 | Iran | 33 | NA | NA | Group 1: Diclofenac 150mg PO Group 2: Pethidine 50mg IM | Adverse events | NSAIDs with Opioids |
| Nissen[^48^](#_ENREF_48) | 1990 | Denmark | 116 | 35:18 40:23 | 45 47 | Group 1: Indomethacin 50mg IV Group 2: Indomethacin 100mg PR | Need for rescue analgesia | NSAIDs with NSAIDs |
| Sommer[^49^](#_ENREF_49) | 1989 | Denmark | 56 | 22:7 17:10 | 57 54 | Group 1: Diclofenac 75mg IM Group 2: Ketogan (containing 7.5mg ketobemidone HCl) 3ml IM | Complete pain relief at 30 min, Adverse events | NSAIDs with Opioids |
| Thompson[^50^](#_ENREF_50) | 1989 | UK | 58 | NA | NA | Group 1: Diclofenac 100mg PR Group 2: Pethidine 100mg + Prochlorperazine 12.5mg injections IV | Complete pain relief at 30 min, Need for rescue analgesia, Adverse events | NSAIDs with Opioids |
| Jonsson[^51^](#_ENREF_51) | 1987 | Sweden | 47 | 30:2 20:5 | 44.5 44 | Group 1: Indomethacin 50mg IV Group 2: Oxyconchloride 5mg + Papaverine 50mg IV | Adverse events | NSAIDs with Opioids |
| Miralles[^52^](#_ENREF_52) | 1987 | Spain | 50 | NA | NA | Group 1: Diclofenac 75mg IM Group 2: Dipyrone 2g | Pain score (VAS 10cm) at 30 min | NSAIDs with NSAIDs |
| Hetherington[^53^](#_ENREF_53) | 1986 | UK | 58 | NA | NA | Group 1: Diclofenac 75mg IM Group 2: Pentazoxine 100mg IM | Need for rescue analgesia, Adverse events | NSAIDs with Opioids |
| Khalifa[^54^](#_ENREF_54) | 1986 | Kuwait | 91 | 46:4 36:5 | 33 32 | Group 1: Diclofenac 50mg IM Group 2: Pethidine 50-100mg + Hyoscine butyl bromide 20mg IV | Complete pain relief at 30 min, Adverse events | NSAIDs with Opioids |
| Lund[^55^](#_ENREF_55) | 1986 | Denmark | 32 | NA | NA | Group 1: Indomethacin 50mg IV Group 2: Pethidine 75mg IV | Complete pain relief at 30 min, Adverse events | NSAIDs with Opioids |
| Persson[^56^](#_ENREF_56) | 1985 | Sweden | 94 | NA | NA | Group 1: Indoprofen 400mg IV Group 2: Oxicone 10mg + Papaverine 20mg IM | Adverse events | NSAIDs with Opioids |
| Warren[^57^](#_ENREF_57) | 1985 | USA | 88/61 | 64:24 49:12 | NA | Group 1: Dezocine10mg IM Group 2: Morphine10mg IM | Adverse events | Opioids with Opioids |
|  | 1985 | USA | 88/61 | 64:24 49:12 | NA | Group 1: Dezocine15mg IM Group 2: Morphine10mg IM | Adverse events | Opioids with Opioids |
| Quilez[^58^](#_ENREF_58) | 1984 | Spain | 38 | NA | NA | Group 1: Diclofenac 75mg IM Group 2: Pentazoxine 30mg IM | Complete pain relief at 30 min | NSAIDs with Opioids |
| Uden[^59^](#_ENREF_59) | 1983 | Sweden | 50 | NA | NA | Group 1: Indomethacin 50mg IV Group 2: Hydromorphine chloride-atropine 1ml SC + Prochlorperazine 25mg PR | Pain score (VAS 100 mm) at 30 min, Complete pain relief at 30 min, Need for rescue analgesia, Adverse events | NSAIDs with Opioids |
| Daljord[^60^](#_ENREF_60) | 1983 | Norway | 67 | NA | NA | Group 1: Indomethacin 50mg IV Group 2: Pethidine 75mg IV | Complete pain relief at 30 min, Need for rescue analgesia, Adverse events | NSAIDs with Opioids |
| Lehtonen[^61^](#_ENREF_61) | 1983 | Finland | 124 | NA | NA | Group 1: Indomethacin 50mg IV Group 2: Pethidine 75mg IV | Complete pain relief at 30 min, Need for rescue analgesia, Adverse events | NSAIDs with Opioids |
| Vignoni[^62^](#_ENREF_62) | 1983 | Italy | 131 | 49:14 53:15 | 39.2 37.6 | Group 1: Diclofenac 75mg IM Group 2: Placebo | Pain score (VAS 10cm) at 30 min, Complete pain relief at 30min,Adverse events | NSAIDs with placebo |
| Lundstam[^63^](#_ENREF_63) | 1982 | Sweden | 66 | NA | NA | Group 1: Diclofenac 50mg IM Group 2: Spasmofen 1ml IM | Adverse events | NSAIDs with Opioids |
| Finlay[^64^](#_ENREF_64) | 1982 | UK | 26 | 12:1 10:3 | 40.5 42.6 | Group 1: Buprenorphine 0.3mg IM Group 2: Pethidine 100mg IM | Adverse events | Opioids with Opioids |
| Elliott[^65^](#_ENREF_65) | 1979 | USA | 99 | NA | NA | Group 1: Butorphanol 2mg IM Group 2: Butorphanol 4mg IM Group 3: Meperidine 80mg IM | Need for rescue analgesia, Adverse events | Opioids with Opioids |

**Note:** NSAIDs: Nonsteroidal anti-inflammatory drugs, IM: Intramuscular route, IV: Intravenous route, PO: Per oral route, PR: Per rectal route, SC: Subcutaneous route, SU: Sublingual route, F: Female, M: Male, NA: Not available.

**References**

1. Al B, Sunar MM, Zengin S, et al. Comparison of IV dexketoprofen trometamol, fentanyl, and paracetamol in the treatment of renal colic in the ED: A randomized controlled trial. *Am J Emerg Med*. 2018;36:571-576.

2. Hosseininejad SM, Amini Ahidashti H, Bozorgi F, et al. Efficacy and Safety of Combination Therapy with Ketorolac and Morphine in Patient with Acute Renal Colic; A Triple-Blind Randomized Controlled Clinical Trial. *Bulletin of emergency and trauma*. 2017;5:165-170.

3. Mozafari J, Masoumi K, Forouzan A, et al. Sublingual Buprenorphine Efficacy in Renal Colic Pain Relief: a Randomized Placebo-Controlled Clinical Trial. *Pain and therapy*. 2017;6:227-234.

4. Cenker E, Serinken M, Uyanik E. Intravenous paracetamol vs ibuprofen in renal colic: a randomised, double-blind, controlled clinical trial. *Urolithiasis*. 2017:1-5.

5. Pathan SA, Mitra B, Straney LD, et al. Delivering safe and effective analgesia for management of renal colic in the emergency department: a double-blind, multigroup, randomised controlled trial. *Lancet (london, england)*. 2016;387:1999-2007.

6. Zamanian F, Jalili M, Moradi-Lakeh M, et al. Morphine Suppository versus Indomethacin Suppository in the Management of Renal Colic: Randomized Clinical Trial. *Pain research and treatment*. 2016;2016:4981585.

7. Kaynar M, Koyuncu F, Buldu, et al. Comparison of the efficacy of diclofenac, acupuncture, and acetaminophen in the treatment of renal colic. *American journal of emergency medicine*. 2015;33:749-753.

8. Shirazi M, Salehipour M, Afrasiabi MA, et al. Analgesic effects and safety of desmopressin, tramadol and indomethacin in patients with acute renal colic; A randomized clinical trial. *Bulletin of emergency and trauma*. 2015;3:41-45.

9. Hosseini MM YA, Ghahramani L, Rastegari M, Ebrahimi AR. Comparison of the therapeutic effects of rectal diclofenac sodium and intramuscular pethidine injection in the treatment of acute renal colic: a randomized clinical trial. *J Clin Trials*. 2015;5:1000225.

10. KandaSwamy GV, Dhanasekaran AK, Elangovan A, et al. Randomized double blinded placebo controlled trial comparing diclofenac and piroxicam in management of acute renal colic and its clinical implications. *Urology journal*. 2015;12:2069-2073.

11. Ay MO, Sebe A, Kozaci N, et al. Comparison of the analgesic efficacy of dexketoprofen trometamol and meperidine HCl in the relief of renal colic. *American journal of therapeutics*. 2014;21:296-303.

12. Masoumi K, Forouzan A, Darian AA, et al. Comparison of clinical efficacy of intravenous acetaminophen with intravenous morphine in acute renal colic: a randomized, double-blind, controlled trial. *Emergency medicine international*. 2014;2014.

13. Payandemehr P, Jalili M, Mostafazadeh Davani B, et al. Sublingual buprenorphine for acute renal colic pain management: a double-blind, randomized controlled trial. *International journal of emergency medicine*. 2014;7:1.

14. Azizkhani R, Pourafzali SM, Baloochestani E, et al. Comparing the analgesic effect of intravenous acetaminophen and morphine on patients with renal colic pain referring to the emergency department: a randomized controlled trial. *Journal of research in medical sciences*. 2013;18:772-776.

15. Narci HU, M. Uzun, H. Yandi, M. Combining 1000 mg oral acetaminophen with 75 mg intramuscular diclofenac of analgesic efficacy for acute renal colic treatment. *Sci Res Essays*. 2012;7:2017-2021.

16. Asgari SA, Asli MM, Madani AH, et al. Treatment of loin pain suspected to be renal colic with papaverine hydrochloride: a prospective double-blind randomised study. *BJU Int*. 2012;110:449-452.

17. Cevik E, Cinar O, Salman N, et al. Comparing the efficacy of intravenous tenoxicam, lornoxicam, and dexketoprofen trometamol for the treatment of renal colic. *The American journal of emergency medicine*. 2012;30:1486-1490.

18. Serinken M, Eken C, Turkcuer I, et al. Intravenous paracetamol versus morphine for renal colic in the emergency department: a randomised double-blind controlled trial. *Emergency medicine journal : EMJ*. 2012;29:902-905.

19. Salameh S HN, Antopolsky M, Ghanem F, Abramovitz Y, Stalnikowics R. Diclofenac versus Tramadol in the Treatment of Renal Colic: A Prospective, Randomized Trial. *The Open Emergency Medicine Journal*. 2011;4:9-13.

20. Grissa MH, Claessens Y-E, Bouida W, et al. Paracetamol vs piroxicam to relieve pain in renal colic. Results of a randomized controlled trial. *The American journal of emergency medicine*. 2011;29:203-206.

21. Glina S, Damiao R, Afif-Abdo J, et al. Efficacy and safety of parecoxib in the treatment of acute renal colic: a randomized clinical trial. *International braz j urol : official journal of the Brazilian Society of Urology*. 2011;37:697-705.

22. Bektas F, Eken C, Karadeniz O, et al. Intravenous paracetamol or morphine for the treatment of renal colic: a randomized, placebo-controlled trial. *Annals of emergency medicine*. 2009;54:568-574.

23. Snir N, Moskovitz B, Nativ O, et al. Papaverine Hydrochloride for the Treatment of Renal Colic: An Old Drug Revisited. A Prospective, Randomized Study. *The Journal of Urology*. 2008;179:1411-1414.

24. Yencilek F, Aktas C, Goktas C, et al. Role of papaverine hydrochloride administration in patients with intractable renal colic: randomized prospective trial. *Urology*. 2008;72:987-990.

25. Sánchez-Carpena J, Domínguez-Hervella F, García I, et al. Comparison of intravenous dexketoprofen and dipyrone in acute renal colic. *European journal of clinical pharmacology*. 2007;63:751-760.

26. Safdar B, Degutis LC, Landry K, et al. Intravenous morphine plus ketorolac is superior to either drug alone for treatment of acute renal colic. *Annals of emergency medicine*. 2006;48:173-181, 181.e171.

27. Altay B, Horasanli K, Sarica K, et al. Double-blind, placebo-controlled, randomized clinical trial of sublingual or intramuscular piroxicam in the treatment of renal colic. A comparative study. *Urologia internationalis*. 2007;79:73-75.

28. Sanchez-Carpena J, Sesma-Sanchez J, Sanchez-Juan C, et al. Comparison of dexketoprofen trometamol and dipyrone in the treatment of renal colic. *Clinical drug investigation*. 2003;23:139-152.

29. Eray O, Cete Y, Oktay C, et al. Intravenous single-dose tramadol versus meperidine for pain relief in renal colic. *European journal of anaesthesiology*. 2002;19:368-370.

30. Wood VM, Christenson JM, Innes GD, et al. The NARC (nonsteroidal anti-inflammatory in renal colic) trial. Single-dose intravenous ketorolac versus titrated intravenous meperidine in acute renal colic: a randomized clinical trial. *CJEM*. 2000;2:83-89.

31. Larkin GL, Peacock WFt, Pearl SM, et al. Efficacy of ketorolac tromethamine versus meperidine in the ED treatment of acute renal colic. *The American journal of emergency medicine*. 1999;17:6-10.

32. Torralba JAN, Montiel, M.R., Pérez, V.B., Nadal, P.V.V., Albacete, M.P. Ketorolaco intramuscular frente a Tramadol subcutáneo en el tratamiento inicial de urgencia del cólico renal. *Archivos Espanoles de Urologia*. 1999;52:435-437.

33. Cohen E, Hafner R, Rotenberg Z, et al. Comparison of ketorolac and diclofenac in the treatment of renal colic. *European journal of clinical pharmacology*. 1998;54:455-458.

34. Supervía A, Pedro-Botet J, Nogués X, et al. Piroxicam fast-dissolving dosage form vs diclofenac sodium in the treatment of acute renal colic: a double-blind controlled trial. *British journal of urology*. 1998;81:27-30.

35. Cordell WH, Wright SW, Wolfson AB, et al. Comparison of intravenous ketorolac, meperidine, and both (balanced analgesia) for renal colic. *Annals of emergency medicine*. 1996;28:151-158.

36. al-Sahlawi KS, Tawfik OM. Comparative study of the efficacy of lysine acetylsalicylate, indomethacin and pethidine in acute renal colic. *European journal of emergency medicine*. 1996;3:183-186.

37. Stein A, Ben Dov D, Finkel B, et al. Single-dose intramuscular ketorolac versus diclofenac for pain management in renal colic. *American journal of emergency medicine*. 1996;14:385-387.

38. Curry C, Kelly AM. Intravenous tenoxicam for the treatment of renal colic. *New Zealand medical journal*. 1995;108:229-230.

39. Cordell WH, Larson TA, Lingeman JE, et al. Indomethacin suppositories versus intravenously titrated morphine for the treatment of ureteral colic. *Annals of emergency medicine*. 1994;23:262-269.

40. Sandhu DP, Iacovou JW, Fletcher MS, et al. A comparison of intramuscular ketorolac and pethidine in the alleviation of renal colic. *British journal of urology*. 1994;74:690-693.

41. Jasani NB, O'Conner RE, Bouzoukis JK. Comparison of hydromorphone and meperidine for ureteral colic. *Academic emergency medicine : official journal of the Society for Academic Emergency Medicine*. 1994;1:539-543.

42. Walden M, Lahtinen J, Elvander E. Analgesic effect and tolerance of ketoprofen and diclofenac in acute ureteral colic. *Scandinavian journal of urology and nephrology*. 1993;27:323-325.

43. Arnau JM, Cami J, Garcia-Alonso F, et al. Comparative study of the efficacy of dipyrone, diclofenac sodium and pethidine in acute renal colic. *European Journal of Clinical Pharmacology*. 1991;40:543-546.

44. Marthak KV, Gokarn AM, Rao AV, et al. A multi-centre comparative study of diclofenac sodium and a dipyrone/spasmolytic combination, and a single-centre comparative study of diclofenac sodium and pethidine in renal colic patients in India. *Current medical research and opinion*. 1991;12:366-373.

45. Oosterlinck W, Philp NH, Charig C, et al. A double-blind single dose comparison of intramuscular ketorolac tromethamine and pethidine in the treatment of renal colic. *Journal of clinical pharmacology*. 1990;30:336-341.

46. González Ramallo VJ, Muiño Míguez A, Rodríguez de Castro E, et al. Intramuscular buprenorphine in the symptomatic treatment of renal colic. *Revista clinica espanola*. 1990;186:414.

47. Indudhara R VS, Sankaranarayanan A. Oral diclofenac sodium in the treatment of acute renal colic. A prospective randomized study. *Clinical Trials Journal*. 1990;27:295-300.

48. Nissen I, Birke H, Olsen JB, et al. Treatment of ureteric colic. Intravenous versus rectal administration of indomethacin. *British journal of urology*. 1990;65:576-579.

49. Sommer P, Kromann-Andersen B, Lendorf A, et al. Analgesic effect and tolerance of Voltaren and Ketogan in acute renal or ureteric colic. *British journal of urology*. 1989;63:4-6.

50. Thompson JF, Pike JM, Chumas PD, et al. Rectal diclofenac compared with pethidine injection in acute renal colic. *BMJ (clinical research ed)*. 1989;299:1140-1141.

51. Jonsson PE, Olsson AM, Petersson BA, et al. Intravenous indomethacin and oxycone-papaverine in the treatment of acute renal colic. A double-blind study. *British journal of urology*. 1987;59:396-400.

52. Miralles R, Camí J, Gutiérrez J, et al. Diclofenac versus dipyrone in acute renal colic: a double-blind controlled trial. *European journal of clinical pharmacology*. 1987;33:527-528.

53. Hetherington JW, Philp NH. Diclofenac sodium versus pethidine in acute renal colic. *British medical journal (clinical research ed)*. 1986;292:237-238.

54. Khalifa MS, Sharkawi MA. Treatment of Pain Owing to Acute Ureteral Obstruction with Prostaglandin-Synthetase Inhibitor: A Prospective Randomized Study. *The Journal of Urology*. 1986;136:393-395.

55. Lund PG, Jensen SK, Therkildsen MH, et al. Treatment of acute pain due to ureteral calculi with intravenous indomethacin or pethidine. *Behandling af akutte ureterstensmerter med indometacin eller petidin intravenost*. 1986;148:1601-1604.

56. Persson NH, Bergqvist D, Melander A, et al. Comparison of a narcotic (oxicone) and a non-narcotic anti-inflammatory analgesic (indoprofen) in the treatment of renal colic. *Acta chirurgica Scandinavica*. 1985;151:105-108.

57. Warren MM, Boyce WH, Evans JW, et al. A double-blind comparison of dezocine and morphine in patients with acute renal and ureteral colic. *The Journal of urology*. 1985;134:457-459.

58. Quílez C, Pérez-Mateo M, Hernández P, et al. Usefulness of a non-steroid anti-inflamatory, sodium diclofenac, in the treatment of renal colic. Comparative study with a spasmolytic and an opiate analgesic. *Medicina clínica*. 1984;82:754-755.

59. Uden P, Rentzhog L, Berger T. A comparative study on the analgesic effects of indomethacin and hydromorphinechloride-atropine in acute, ureteral-stone pain. *Acta chirurgica Scandinavica*. 1983;149:497-499.

60. Daljord OA, Barstad S, Norenberg P. Ambulatory treatment of an acute attack in urinary calculi. A randomized study of the effects of Petidin, Fortralin, Temgesic and Confortid. *Poliklinisk behandling av akutte nyrestenanfall Randomisert studie over effekten av Petidin, Fortralin, Temgesic og Confortid*. 1983;103:1006-1008.

61. Lehtonen T, Kellokumpu I, Permi J, et al. Intravenous indomethacin in the treatment of ureteric colic. A clinical multicentre study with pethidine and metamizol as the control preparations. *Annals of clinical research*. 1983;15:197-199.

62. Vignoni A, Fierro A, Moreschini G, et al. Diclofenac sodium in ureteral colic: a double-blind comparison trial with placebo. *The Journal of international medical research*. 1983;11:303-307.

63. Lundstam SO, Leissner KH, Wåhlander LA, et al. Prostaglandin-synthetase inhibition with diclofenac sodium in treatment of renal colic: comparison with use of a narcotic analgesic. *Lancet (london, england)*. 1982;1:1096-1097.

64. Finlay IG, Scott R, McArdle CS. Prospective double-blind comparison of buprenorphine and pethidine in ureteric colic. *British medical journal (Clinical research ed)*. 1982;284:1830-1831.

65. Elliott JP, Evans JW, Gordon JO, et al. Butorphanol and meperidine compared in patients with acute ureteral colic. *The Journal of urology*. 1979;122:455-457.

**Supplement Table 2. The results of network meta-analysis of NSAIDs, opioids, paracetamol, combination therapy and placebo for failure of ≥50% pain relief at 30 min and need for rescue analgesia from first stage.**

| NSAIDs | **0.57 (0.39, 0.83)** | **0.48 (0.26, 0.84)** | **2.35 (1.13, 4.92)** | **0.21 (0.06, 0.80)** |
| --- | --- | --- | --- | --- |
| 0.51 (0.16, 1.48) | Opioids | 0.85 (0.46, 1.48) | **4.11 (1.94, 9.05)** | 0.37 (0.10, 1.40) |
| 0.76 (0.13, 4.26) | 1.48 (0.25, 8.27) | Paracetamol | **4.85 (2.03, 12.16)** | 0.43 (0.12, 1.69) |
| 2.27 (0.34, 16.64) | 4.45 (0.68, 31.48) | 3.10 (0.24, 42.07) | Combination therapy | **0.09 (0.02, 0.42)** |
| -- | -- | -- | -- | Placebo |

**Note:** Comparisons between treatments should be read from left to right and the estimate is in the cell in common between the upper-left-defining treatment and the lower-right-defining treatment. For failure of ≥50% pain relief at 30 min in the lower left corner and need for rescue analgesia in the upper right corner from Stage I, the odds ratios (ORs) lower than 1 favour the upper-left-defining treatment. To obtain ORs for comparisons in the opposite direction, reciprocals should be taken. Significant results are in bold and underlined, and -- means that the results are not available. NSAIDs: Nonsteroidal anti-inflammatory drugs.

**Supplement Table 3.** **The results of network meta-analysis of NSAIDs, opioids, paracetamol, combination therapy and placebo for nonspecific acute adverse events and vomiting as an adverse event from first stage.**

| NSAIDs | **0.36 (0.21, 0.60)** | 0.63 (0.25, 1.52) | 0.55 (0.13, 2.32) | 2.40 (0.33, 19.29) |
| --- | --- | --- | --- | --- |
| **0.31 (0.20, 0.50)** | Opioids | 1.75 (0.72, 4.20) | 1.53 (0.38, 6.57) | 6.52 (0.97, 54.56) |
| 0.94 (0.35, 2.49) | **2.96 (1.04, 8.33)** | Paracetamol | 0.88 (0.18, 4.52) | 3.72 (0.56, 31.71) |
| **0.34 (0.14, 0.86)** | 1.10 (0.44, 2.97) | 0.38 (0.10, 1.39) | Combination therapy | 4.27 (0.44, 52.29) |
| 1.03 (0.02, 65.72) | 3.25 (0.06, 207.0) | 1.16 (0.02, 72.58) | 3.05 (0.05, 213.9) | Placebo |

**Note:** Comparisons between treatments should be read from left to right and the estimate is in the cell in common between the upper-left-defining treatment and the lower-right-defining treatment. For nonspecific acute adverse events in the lower left corner and vomiting as an adverse event in the upper right corner from Stage I, the odds ratios (ORs) lower than 1 favour the upper-left-defining treatment. To obtain ORs for comparisons in the opposite direction, reciprocals should be taken. Significant results are in bold and underlined. NSAIDs: Nonsteroidal anti-inflammatory drugs.

**Supplement Table 4.** **The results of network meta-analysis of NSAIDs, opioids, paracetamol, combination therapy and placebo with different routes for failure of ≥50% pain relief at 30 min and need for rescue analgesia from second stage.**

| NSAIDs, IM | 0.85  (0.34, 2.14) | 0.57  (0.18, 2.17) | 0.83  (0.23, 3.11) | 1.11  (0.43, 3.88) | **0.36**  **(0.15, 0.77)** | 0.56  (0.13, 2.57) | **0.36**  **(0.13, 0.87)** | 0.23  (0.02, 1.98) | 2.44  (0.37, 15.41) | 1.34  (0.37, 4.88) | 0.91  (0.06, 14.53) | **0.15**  **(0.03, 0.71)** |
| --- | --- | --- | --- | --- | --- | --- | --- | --- | --- | --- | --- | --- |
| **8.25**  **(1.01, 64.86)** | NSAIDs, IV | 0.69  (0.23, 2.15) | 0.99  (0.19, 4.72) | 1.33  (0.43, 5.23) | **0.42**  **(0.22, 0.76)** | 0.66  (0.17, 2.72) | **0.42**  **(0.18, 0.89)** | 0.27  (0.01, 2.77) | 2.87  (0.5, 16.28) | 1.58  (0.54, 4.33) | 1.04  (0.06, 18.88) | **0.18**  **(0.04, 0.74)** |
| 0.30  (0.04, 2.30) | **0.04**  **(0.002, 0.74)** | NSAIDs, PR | 1.44  (0.22, 8.12) | 1.99  (0.59, 6.86) | 0.61  (0.18, 1.86) | 0.95  (0.18, 5.63) | 0.61  (0.16, 1.99) | 0.39  (0.02, 4.43) | **4.17**  **(1.01, 17.16)** | 2.29  (0.51, 9.88) | 1.51  (0.08, 27.61) | 0.26  (0.04, 1.33) |
| -- | -- | -- | NSAIDs, SU | 1.36  (0.29, 8.51) | 0.43  (0.09, 1.91) | 0.68  (0.10, 4.38) | 0.44  (0.09, 2.04) | 0.27  (0.01, 3.50) | 2.93  (0.28, 31.07) | 1.60  (0.25, 9.94) | 1.12  (0.06, 22.08) | 0.18  (0.02, 1.44) |
| 0.53  (0.12, 2.27) | **0.06**  **(0.01, 0.83)** | 1.75  (0.44, 6.74) | -- | Opioids, IM | **0.32**  **(0.08, 0.93)** | 0.49  (0.08, 2.69) | 0.32  (0.07, 1.02) | 0.20  (0.01, 1.95) | 2.12  (0.31, 13.28) | 1.18  (0.22, 5.01) | 0.77  (0.04, 14.09) | **0.13**  **(0.02, 0.68)** |
| 0.74  (0.20, 2.71) | **0.09**  **(0.02, 0.46)** | 2.46  (0.22, 24.54) | -- | 1.41  (0.20, 9.17) | Opioids, IV | 1.54  (0.38, 7.09) | 1.01  (0.50, 1.95) | 0.66  (0.04, 6.70) | **6.85**  **(1.14, 43.24)** | **3.75**  **(1.36, 11.02)** | 2.57  (0.15, 44.62) | 0.42  (0.10, 1.68) |
| -- | -- | -- | -- | -- | -- | Opioids, SC | 0.64  (0.13, 2.82) | 0.38  (0.02, 5.13) | 4.41  (0.48, 37.02) | 2.39  (0.43, 12.91) | 1.61  (0.07, 32.94) | 0.27  (0.03, 1.94) |
| 0.94  (0.27, 3.07) | **0.11**  **(0.01, 0.85)** | 3.11  (0.26, 31.74) | -- | 1.77  (0.25, 11.69) | 1.27  (0.35, 4.50) | -- | Paracetamol, IV | 0.65  (0.04, 6.67) | **6.77**  **(1.09, 48.51)** | **3.72**  **(1.10, 12.65)** | 2.53  (0.14, 48.97) | 0.42  (0.11, 1.64) |
| -- | -- | -- | -- | -- | -- | -- | -- | Paracetamol, PO | 11.3  (0.59, 341.3) | 5.79  (0.48, 111.5) | 4.14  (0.53, 46.42) | 0.68  (0.04, 14.43) |
| -- | -- | -- | -- | -- | -- | -- | -- | -- | NSAIDs, PR + Opioids, IV | 0.55  (0.07, 3.90) | 0.38  (0.01, 8.78) | **0.06**  **(0.01, 0.54)** |
| 7.88  (0.89, 59.14) | 0.96  (0.17, 4.98) | **25.76**  **(1.48, 464.2)** | -- | **14.88**  **(1.18, 176.2)** | **10.48**  **(2.03, 54.13)** | -- | **8.45**  **(1.07, 63.78)** | -- | -- | NSAIDs, IV + Opioids, IV | 0.67  (0.03, 13.34) | **0.11**  **(0.02, 0.59)** |
| -- | -- | -- | -- | -- | -- | -- | -- | -- | -- | -- | NSAIDs, IM + Paracetamol, PO | 0.16  (0.01, 4.08) |
| -- | -- | -- | -- | -- | -- | -- | -- | -- | -- | -- | -- | Placebo |

**Note:** Comparisons between treatments should be read from left to right and the estimate is in the cell in common between the upper-left-defining treatment and the lower-right-defining treatment. For failure of ≥50% pain relief at 30 min in the lower left corner and need for rescue analgesia in the upper right corner from Stage II, the odds ratios (ORs) lower than 1 favour the upper-left-defining treatment. To obtain ORs for comparisons in the opposite direction, reciprocals should be taken. The consistency model with random effects was employed for failure of ≥50% pain relief at 30 minutes, while the design-by-treatment interaction model with random inconsistency effects was employed for need for rescue analgesia (heterogeneity: 0.410, inconsistency: 0.361). Significant results are in bold and underlined, and “--“ means that the results are not available. NSAIDs: Nonsteroidal anti-inflammatory drugs, IM: Intramuscular route, IV: Intravenous route, PO: Per oral route, PR: Per rectal route, SC: Subcutaneous route, SU: Sublingual route.

**Supplement Table 5.** **The results of network meta-analysis of NSAIDs, opioids, paracetamol, combination therapy and placebo with different routes for nonspecific acute adverse events and vomiting as an adverse event from second stage.**

| NSAIDs, IM | 0.88  (0.10, 7.84) | 0.79  (0.04, 13.09) | 1.00  (0.02, 57.16) | 0.45  (0.13, 1.69) | 0.23  (0.04, 1.38) | 0.77  (0.03, 23.51) | 5.35  (0.05, 4.13E+03) | **0.05**  **(0.003, 0.67)** | 0.44  (0.07, 2.44) | 0.97  (0.001, 1.04E+03) | 0.05  (7.19E-05, 3.19) | -- | 0.51  (0.04, 6.48) | 0.92  (9.41E-04, 1.02E+03) | -- | 1.57  (0.14, 20.24) |
| --- | --- | --- | --- | --- | --- | --- | --- | --- | --- | --- | --- | --- | --- | --- | --- | --- |
| 0.64  (0.19, 2.01) | NSAIDs, IV | 0.83  (0.07, 11.22) | 1.09  (0.01, 1.27E+02) | 0.50  (0.04, 6.21) | **0.26**  **(0.07, 0.86)** | 0.85  (0.04, 20.11) | 5.78  (0.11, 4.73E+03) | **0.06**  **(0.008, 0.36)** | 0.48  (0.11, 1.97) | 1.06  (0.001, 1.59E+03) | 0.06  (1.04E-04, 1.94) | -- | 0.57  (0.08, 4.12) | 0.96  (8.99E-04, 1.62E+03) | -- | 1.77  (0.16, 21.33) |
| **0.02**  **(4.17E-05, 0.65)** | 0.04  (7.30E-05, 1.34) | NSAIDs, PR | 1.21  (0.008, 2.46E+02) | 0.58  (0.02, 14.05) | 0.31  (0.03, 2.38) | 1.01  (0.16, 6.45) | 6.97  (0.06, 7.51E+03) | **0.07**  **(0.003, 1.21)** | 0.57  (0.05, 6.07) | 1.26  (8.58E-04, 2.52E+03) | 0.07  (8.33E-05, 5.72) | -- | 0.67  (0.04, 11.51) | 1.23  (9.06E-04, 2.41E+03) | -- | 2.03  (0.1, 45.33) |
| 0.39  (0.010, 6.22) | 0.59  (0.01, 12.21) | 16.53  (0.11, 1.57E+04) | NSAIDs, SU | 0.47  (0.007, 29.39) | 0.23  (0.003, 17.63) | 0.84  (0.003, 1.45E+02) | 5.80  (0.009, 1.07E+04) | 0.05  (3.96E-04, 5.65) | 0.46  (0.005, 37.01) | 0.98  (4.72E-04, 1.87E+03) | 0.05  (3.02E-05, 21.69) | -- | 0.51  (0.004, 51.06) | 0.84  (4.38E-04, 2.93E+03) | -- | 1.65  (0.01, 1.96E+02) |
| **0.22**  **(0.11, 0.42)** | 0.35  (0.09, 1.31) | 8.88  (0.33, 4.66E+03) | 0.58  (0.03, 25.96) | Opioids, IM | 0.51  (0.05, 4.59) | 1.70  (0.04, 63.53) | 12.08  (0.09, 1.07E+04) | 0.11  (0.006, 1.99) | 0.97  (0.11, 8.54) | 2.16  (0.003, 2.28E+03) | 0.10  (1.45E-04, 9.39) | -- | 1.14  (0.07, 19.12) | 2.02  (0.002, 2.99E+03) | -- | 3.58  (0.23, 61.93) |
| **0.28**  **(0.10, 0.74)** | **0.43**  **(0.21, 0.91)** | 11.48  (0.32, 7.05E+03) | 0.73  (0.04, 33.37) | 1.27  (0.37, 4.20) | Opioids, IV | 3.38  (0.21, 59.77) | 23.84  (0.36, 1.74E+04) | 0.22  (0.03, 1.34) | 1.92  (0.65, 5.56) | 4.32  (0.005, 5.06E+03) | 0.23  (4.29E-04, 10.23) | -- | 2.19  (0.38, 16.60) | 3.87  (0.004, 5.33E+03) | -- | **6.81**  **(0.95, 64.31)** |
| -- | -- | -- | -- | -- | -- | Opioids, PR | 7.31  (0.04, 8.16E+03) | 0.07  (0.002, 2.20) | 0.55  (0.03, 11.46) | 1.23  (6.26E-04, 3.06E+03) | 0.07  (6.67E-05, 7.36) | -- | 0.65  (0.02, 18.69) | 1.19  (7.22E-04, 2.72E+03) | -- | 2.07  (0.07, 73.8) |
| 1.05  (0.10, 10.57) | 1.73  (0.17, 15.11) | 47.10  (0.80, 2.80E+04) | 2.90  (0.09, 2.43E+02) | 5.01  (0.43, 55.06) | 4.05  (0.38, 36.66) | -- | Opioids, SC | **0.01**  **(9.80E-06, 0.85)** | 0.08  (1.15E-04, 5.36) | 0.15  (1.64E-05, 5.79E+02) | 0.01  (1.56E-06, 2.04) | -- | 0.09  (1.19E-04, 9.16) | 0.14  (1.74E-05, 5.48E+02) | -- | 0.29  (3.90E-04, 29.02) |
| -- | -- | -- | -- | -- | -- | -- | -- | Opioids, SU | **8.57**  **(1.12, 72.22)** | 19.44  (0.02, 2.97E+04) | 1.02  (0.002, 66.35) | -- | **9.97**  **(0.83, 1.57E+02)** | 16.48  (0.01, 3.86E+04) | -- | **32.31**  **(1.86, 6.36E+02)** |
| 0.81  (0.26, 2.55) | 1.28  (0.42, 4.55) | 35.01  (1.04, 1.90E+04) | 2.18  (0.11, 1.03E+02) | **3.75**  **(1.05, 13.79)** | **3.00**  **(1.08, 9.09)** | -- | 0.76  (0.07, 9.38) | -- | Paracetamol, IV | 2.23  (0.002, 2.70E+03) | 0.13  (1.60E-04, 5.61) | -- | 1.15  (0.16, 10.92) | 2.00  (0.002, 2.83E+03) | -- | 3.64  (0.47, 34.64) |
| 1.01  (0.002, 1.04E+03) | 1.59  (0.003, 1.81E+03) | 55.32  (0.04, 3.11E+05) | 2.84  (0.003, 7.46E+03) | 4.66  (0.009, 4.89E+03) | 3.73  (0.007, 3.97E+03) | -- | 1.03  (0.001, 1.47E+03) | -- | 1.20  (0.002, 1.36E+03) | Paracetamol, PO | 0.04  (6.19E-06, 1.34E+02) | -- | 0.52  (3.39E-04, 6.82E+02) | 0.93  (0.001, 9.47E+02) | -- | 1.77  (0.001, 2.08E+03) |
| **0.01**  **(3.21E-04, 0.19)** | **0.02**  **(5.74E-04, 0.20)** | 0.48  (0.004, 3.47E+02) | 0.03  (3.29E-04, 2.64) | **0.05**  **(0.001, 0.94)** | **0.04**  **(0.001, 0.58)** | -- | 0.01  (2.18E-04, 0.29) | -- | **0.01**  **(3.76E-04, 0.22)** | 0.01  (4.62E-06, 9.32) | Double Opioids, IM | -- | 9.45  (0.17, 8.29E+03) | 20.67  (0.01, 2.15E+05) | -- | 32.57  (0.47, 2.63E+04) |
| 0.34  (0.03, 3.28) | 0.53  (0.08, 3.30) | 15.01  (0.23, 8.57E+03) | 0.96  (0.02, 56.90) | 1.55  (0.14, 15.29) | 1.24  (0.16, 8.93) | -- | 0.31  (0.02, 6.12) | -- | 0.42  (0.04, 3.84) | 0.32  (2.91E-04, 2.21E+02) | **29.57**  **(1.43, 1.42E+03)** | Double Opioids, IV | -- | -- | -- | -- |
| 0.41  (0.08, 1.73) | 0.63  (0.20, 2.03) | 17.38  (0.43, 1.09E+04) | 1.08  (0.05, 56.47) | 1.85  (0.34, 9.65) | 1.47  (0.45, 4.54) | -- | 0.37  (0.03, 4.48) | -- | 0.49  (0.10, 2.13) | 0.38  (3.41E-04, 2.52E+02) | **34.42**  **(2.24, 1.20E+03)** | 1.17  (0.14, 10.23) | NSAIDs, IV + Opioids, IV | 1.70  (0.002, 2.79E+03) | -- | 3.13  (0.20, 52.0) |
| -- | -- | -- | -- | -- | -- | -- | -- | -- | -- | -- | -- | -- | -- | NSAIDs, IM + Paracetamol, PO | -- | 1.80  (0.001, 2.14E+03) |
| 1.01  (0.003, 7.81E+02) | 1.59  (0.004, 1.62E+03) | 51.66  (0.04, 3.20E+05) | 2.96  (0.003, 4.94E+03) | 4.68  (0.01, 3.78E+03) | 3.68  (0.008, 3.61E+03) | -- | 0.96  (0.001, 1.27E+03) | -- | 1.26  (0.003, 1.16E+03) | 0.95  (0.001, 7.92E+02) | 98.16  (0.12, 1.61E+05) | 2.92  (0.005, 4.15E+03) | 2.53  (0.006, 2.60E+03) | -- | NSAIDs, IV + Paracetamol, PO | -- |
| 1.14  (0.002, 9.80E+02) | 1.81  (0.003, 1.35E+03) | 55.75  (0.04, 4.51E+05) | 3.61  (0.003, 5.17E+03) | 5.21  (0.009, 4.39E+03) | 4.21  (0.007, 3.23E+03) | -- | 1.05  (0.001, 1.38E+03) | -- | 1.37  (0.002, 1.11E+03) | 1.03  (1.17E-04, 1.20E+04) | 111.2  (0.11, 2.00E+05) | 3.41  (0.004, 4.06E+03) | 2.86  (0.005, 2.35E+03) | -- | 1.10  (1.30E-04, 1.41E+04) | Placebo |

**Note:** Comparisons between treatments should be read from left to right and the estimate is in the cell in common between the upper-left-defining treatment and the lower-right-defining treatment. For nonspecific acute adverse events in the lower left corner and vomiting as an adverse event in the upper right corner from Stage II, the odds ratios (ORs) lower than 1 favour the upper-left-defining treatment. To obtain ORs for comparisons in the opposite direction, reciprocals should be taken. The consistency model with random effects was employed for nonspecific acute adverse events, while the design-by-treatment interaction model with random inconsistency effects was employed for vomiting as an adverse event (heterogeneity: 0.668, inconsistency: 0.427). Significant results are in bold and underlined, and “--“ means that the results are not available. NSAIDs: Nonsteroidal anti-inflammatory drugs, IM: Intramuscular route, IV: Intravenous route, PO: Per oral route, PR: Per rectal route, SC: Subcutaneous route, SU: Sublingual route.

**Supplement Table 6. The sensitivity analyses results of network analysis from second stage.**

| **Pain variance at 30 min** | | | | | | | | | | | | | | | |
| --- | --- | --- | --- | --- | --- | --- | --- | --- | --- | --- | --- | --- | --- | --- | --- |
| **Intervention of different routes** | **Main results** | | |  | **Excluded placebo** | | |  | **Excluded single blinded and unblinded study** | | |  | **Pain scale: VAS 10mm; VAS 100mm** | | |
|  | **MD,**  **95% CrIs** | **SUCRA (%)** | **Ranking, 95% CrIs** |  | **MD,**  **95% CrIs** | **SUCRA (%)** | **Ranking, 95% CrIs** |  | **MD,**  **95% CrIs** | **SUCRA (%)** | **Ranking, 95% CrIs** |  | **MD, 95% CrIs** | **SUCRA (%)** | **Ranking, 95% CrIs** |
| **NSAIDs, IM** | Reference | 70.61 | 3.9  (2, 6) |  | Reference | 67.43 | 3.93  (2, 6) |  | Reference | 69.55 | 3.4  (3, 5) |  | Reference | 86.28;  45.03 | 1.82 (1, 3);  5.4 (4, 7) |
| **NSAIDs, IV** | 6.92  (-1.62, 15.51) | 88.36 | 2.2  (1, 4) |  | 6.84  (-1.73, 15.4) | 86.91 | 2.18  (1, 4) |  | 10.77  (1.53, 20.33) | 90.28 | 1.8  (1, 3) |  | 1.18 (-13.01, 15.12);  13.47 (2.70, 24.88) | 83.61;  88.4 | 1.98 (1, 6);  1.93 (1, 4) |
| **NSAIDs, PR** | -12.91  (-27.02, 1.46) | 23.11 | 8.7  (4, 11) |  | -12.97  (-27.45, 1.99) | 17.03 | 8.47  (4, 10) |  | NA | NA | NA |  | -17.5 (-32.38, -2.27);  NA | 9.728;  NA | 6.42 (3, 7);  NA |
| **NSAIDs, SU** | -9.55  (-26.09, 6.93) | 34.01 | 7.6  (2, 11) |  | -9.09  (-25.52, 6.67) | 29.37 | 7.36  (2, 10) |  | -9.51  (-25.68, 6.48) | 32.95 | 6.4  (3, 9) |  | -9.28 (-26.06, 6.44);  NA | 38.49;  NA | 4.69 (1, 7);  NA |
| **Opioids, IM** | -1.98  (-8.60, 4.60) | 60.59 | 4.9  (2, 8) |  | -2.03  (-8.35, 4.70) | 56.06 | 4.95  (2, 8) |  | -6.78  (-14.79, 1.61) | 39.68 | 5.8  (3, 8) |  | -6.67 (-15.23, 1.54);  5.88 (-3.70, 15.45) | 48.44;  66.03 | 4.09 (1, 6);  3.72 (1, 7) |
| **Opioids, IV** | -7.27  (-13.4, -1.08) | 35.93 | 7.4  (5, 10) |  | -7.52  (-13.75, -1.21) | 28.39 | 7.45  (5, 10) |  | -7.03  (-13.87, 0.13) | 36.15 | 6.1  (4, 8) |  | -8.09 (-16.07, -0.30);  -6.54 (-15.7, 3.02) | 38.09;  24.4 | 4.72 (3, 7);  7.05 (5, 8) |
| **Opioids, SC** | -1.50  (-19.24, 17.54) | 58.86 | 5.1  (1, 10) |  | -1.66  (-19.87, 16.09) | 54.27 | 5.12  (1, 10) |  | NA | NA | NA |  | NA;  5.23 (-12.41, 23.49) | NA;  62.26 | NA;  4.02 (1, 8) |
| **Paracetamol, IV** | -4.20  (-10.41, 1.73) | 50.84 | 5.9  (4, 8) |  | -4.33  (-10.66, 1.71) | 45.54 | 5.9  (3, 8) |  | -2.82  (-10.1, 4.4) | 57.42 | 4.4  (3, 7) |  | -7.07 (-14.87, 0.52);  2.72 (-7.19, 12.94) | 45.36;  55.2 | 4.28 (2, 7);  4.58 (3, 7) |
| **Paracetamol, PO** | -12.73  (-26.09, 0.25) | 23.60 | 8.6  (4, 11) |  | -12.25  (-25.63, 0.96) | 17.56 | 8.42  (4, 10) |  | -12.81  (-25.5, 0.3) | 21.67 | 7.3  (4, 9) |  | NA;  -12.38 (-24.43, -0.36) | NA;  14.47 | NA;  7.84 (6, 9) |
| **NSAIDs, IM + Paracetamol, PO** | 16.33  (1.62, 31.51) | 97.24 | 1.3  (1, 3) |  | 16.58  (2.07, 32.0) | 97.43 | 1.23  (1, 3) |  | 16.44  (1.81, 31.1) | 96.29 | 1.3  (1, 3) |  | NA;  16.55 (3.09, 30.45) | NA;  91.12 | NA;  1.71 (1, 5) |
| **Placebo** | -20.92  (-34.59, -7.24) | 6.863 | 10.3  (7, 11) |  | NA | NA | NA |  | -20.6  (-35.04, -5.96) | 6.008 | 8.5  (6, 9) |  | NA;  -20.53 (-33.52, -7.82) | NA;  3.083 | NA;  8.75 (7, 9) |
| **Failure of complete relief at 30 min** | | | | | | | | | | | | | | | |
| **Intervention of different routes** | **Main results** | | |  | **Excluded placebo** | | |  | **Excluded single blinded and unblinded study** | | |  | **Excluded "0" study** | | |
|  | **OR,**  **95% CI** | **SUCRA (%)** | **Ranking, 95% CI** |  | **OR,**  **95% CI** | **SUCRA (%)** | **Ranking, 95% CI** |  | **OR,**  **95% CI** | **SUCRA (%)** | **Ranking, 95% CI** |  | **OR, 95% CI** | **SUCRA (%)** | **Ranking, 95% CI** |
| **NSAIDs, IM** | Reference | 59.06 | 4.7  (1, 9) |  | Reference | 55.35 | 4.6  (1, 9) |  | Reference^a^ | 72.54^a^ | 1.8  (1, 3) ^a^ |  | Reference | 58.74 | 4.7  (1, 9) |
| **NSAIDs, IV** | 0.79  (0.10, 6.22) | 50.47 | 5.5  (2, 9) |  | 0.78  (0.10, 5.15) | 46.94 | 5.2  (2, 8) |  | Reference^b^ | 37.32^b^ | 2.9  (1, 4) ^b^ |  | 0.83  (0.11, 5.81) | 51.46 | 5.4  (2, 9) |
| **NSAIDs, PR** | 0.76  (0.11, 5.79) | 48.61 | 5.6  (1, 10) |  | 0.78  (0.11, 5.60) | 45.97 | 5.3  (1, 9) |  | NA | NA | NA |  | 0.78  (0.11, 5.39) | 48.24 | 5.7  (1, 10) |
| **NSAIDs, SU** | 1.14  (0.14, 8.96) | 60.96 | 4.5  (1, 10) |  | 1.17  (0.15, 9.08) | 59.14 | 4.3  (1, 9) |  | 1.20^a^  (0.16, 8.57) | 74.47^a^ | 1.8  (1, 4) ^a^ |  | 1.15  (0.16, 9.09) | 61.26 | 4.5  (1, 10) |
| **Opioids, IM** | 1.25  (0.27, 5.94) | 66.71 | 4.0  (1, 9) |  | 1.26  (0.28, 5.69) | 64.65 | 3.8  (1, 8) |  | 0.42  (0.06, 2.94)^a^ | 32.41^a^ | 3.0  (1, 4) ^a^ |  | 1.24  (0.28, 6.09) | 66.19 | 4.0  (1, 9) |
| **Opioids, IV** | 0.72  (0.12, 4.31) | 45.53 | 5.9  (2, 9) |  | 0.69  (0.13, 3.83) | 40.79 | 5.7  (2, 9) |  | 0.99  (0.37, 2.77) ^b^ | 36.37^b^ | 2.9  (1, 4) ^b^ |  | 0.74  (0.13, 4.16) | 45.27 | 5.9  (2, 9) |
| **Opioids, SC** | 0.22  (0.01, 4.60) | 17.63 | 8.4  (2, 10) |  | 0.21  (0.01, 3.63) | 13.94 | 7.9  (2, 9) |  | NA | NA | NA |  | 0.21  (0.01, 3.96) | 16.69 | 8.5  (2, 10) |
| **Paracetamol, IV** | 0.78  (0.06, 9.15) | 49.88 | 5.5  (1, 10) |  | 0.74  (0.07, 9.17) | 45.49 | 5.4  (1, 9) |  | 1.03  (0.19, 5.18) ^b^ | 41.01^b^ | 2.8  (1, 4) ^b^ |  | 0.77  (0.07, 9.00) | 50.39 | 5.5  (1, 10) |
| **NSAIDs, IV + Opioids, IV** | 2.00  (0.16, 30.6) | 79.11 | 2.9  (1, 9) |  | 1.99  (0.16, 24.2) | 77.73 | 2.8  (1, 8) |  | 2.62  (0.42, 14.6) ^b^ | 85.3^b^ | 1.4  (1, 4) ^b^ |  | 2.14  (0.16, 24.7) | 79.98 | 2.8  (1, 9) |
| **Placebo** | 0.29  (0.04, 1.93) | 22.03 | 8.0  (2, 10) |  | NA | NA | NA |  | 0.29  (0.05, 1.76) ^a^ | 20.58 ^a^ | 3.4  (1, 4) ^a^ |  | 0.29  (0.04, 2.17) | 21.78 | 8.0  (2, 10) |
| **Need for rescue analgesia** | | | | | | | | | | | | | | | |
| **Intervention of different routes** | **Main results** | | |  | **Excluded placebo** | | |  | **Excluded single blinded and unblinded study** | | |  | **Excluded "0" study** | | |
|  | **OR,**  **95% CI** | **SUCRA (%)** | **Ranking, 95% CI** |  | **OR,**  **95% CI** | **SUCRA (%)** | **Ranking, 95% CI** |  | **OR,**  **95% CI** | **SUCRA (%)** | **Ranking, 95% CI** |  | **OR, 95% CI** | **SUCRA (%)** | **Ranking, 95% CI** |
| **NSAIDs, IM** | Reference | 67.98 | 4.8  (2, 8) |  | Reference | 64.52 | 4.9  (2, 8) |  | Reference | 70.39 | 4.3  (2, 7) |  | Reference | 67.19 | 4.9  (2, 8) |
| **NSAIDs, IV** | 0.85  (0.34, 2.14) | 60.61 | 5.7  (2, 9) |  | 0.86  (0.32, 2.46) | 56.51 | 5.8  (2, 9) |  | 1.02  (0.45, 2.51) | 70.09 | 4.3  (2, 7) |  | 0.90  (0.36, 2.23) | 62.08 | 5.6  (2, 9) |
| **NSAIDs, PR** | 0.57  (0.18, 2.17) | 44.15 | 7.7  (3, 12) |  | 0.59  (0.17, 2.22) | 39.26 | 7.7  (3, 12) |  | 0.26  (0.05, 1.38) | 18.28 | 10  (4, 12) |  | 0.58  (0.19, 1.97) | 43.29 | 7.8  (3, 12) |
| **NSAIDs, SU** | 0.83  (0.23, 3.11) | 58.55 | 6.0  (1, 12) |  | 0.84  (0.20, 3.42) | 54.49 | 6.0  (1, 12) |  | 0.84  (0.30, 2.13) | 59.5 | 5.5  (1, 10) |  | 0.81  (0.23, 2.78) | 56.86 | 6.2  (1, 12) |
| **Opioids, IM** | 1.11  (0.43, 3.88) | 72.16 | 4.3  (1, 9) |  | 1.12  (0.38, 4.12) | 68.54 | 4.5  (1, 10) |  | 0.86  (0.37, 2.28) | 60.97 | 5.3  (1, 10) |  | 1.12  (0.41, 3.11) | 71.23 | 4.5  (1, 9) |
| **Opioids, IV** | 0.36  (0.15, 0.77) | 23.97 | 10.1  (7, 12) |  | 0.36  (0.14, 0.82) | 18.18 | 10  (7, 12) |  | 0.51  (0.26, 1.06) | 36.88 | 7.9  (5, 10) |  | 0.36  (0.15, 0.78) | 23.16 | 10.2  (7, 12) |
| **Opioids, SC** | 0.56  (0.13, 2.57) | 43.54 | 7.8  (2, 13) |  | 0.57  (0.12, 2.55) | 39.39 | 7.7  (2, 12) |  | NA | NA | NA |  | 0.58  (0.13, 2.62) | 43.71 | 7.8  (2, 13) |
| **Paracetamol, IV** | 0.36  (0.13, 0.87) | 25.10 | 10.0  (6, 12) |  | 0.35  (0.12, 0.93) | 18.48 | 10  (6, 12) |  | 0.48  (0.23, 0.98) | 33.28 | 8.3  (5, 11) |  | 0.37  (0.13, 0.98) | 24.94 | 10  (7, 12) |
| **Paracetamol, PO** | 0.23  (0.02, 1.98) | 20.55 | 10.5  (2, 13) |  | 0.25  (0.02, 2.09) | 19.23 | 9.9  (2, 12) |  | 0.24  (0.02, 1.56) | 19.79 | 9.8  (3, 12) |  | 0.25  (0.02, 2.16) | 21.2 | 10.5  (3, 13) |
| **NSAIDs, PR + Opioids, IV** | 2.44  (0.37, 15.4) | 88.98 | 2.3  (1, 9) |  | 2.38  (0.35, 17.8) | 86.41 | 2.5  (1, 9) |  | 1.06  (0.17, 8.3) | 67.62 | 4.6  (1, 11) |  | 2.37  (0.41, 16.3) | 88.91 | 2.3  (1, 8) |
| **NSAIDs, IV + Opioids, IV** | 1.34  (0.37, 4.88) | 77.12 | 3.7  (1, 9) |  | 1.36  (0.34, 5.65) | 74.75 | 3.8  (1, 9) |  | 1.74  (0.60, 5.89) | 89.06 | 2.2  (1, 6) |  | 1.36  (0.37, 5.12) | 77.79 | 3.7  (1, 9) |
| **NSAIDs, IM + Paracetamol, PO** | 0.91  (0.06, 14.5) | 59.57 | 5.9  (1, 13) |  | 1.06  (0.07, 14.8) | 60.23 | 5.4  (1, 12) |  | 0.98  (0.09, 13.1) | 63.31 | 5.0  (1, 11) |  | 1.08  (0.07, 14.3) | 62.96 | 5.4  (1, 12) |
| **Placebo** | 0.15  (0.03, 0.71) | 7.722 | 12.1  (8, 13) |  | NA | NA | NA |  | 0.20  (0.06, 0.72) | 10.83 | 10.8  (7, 12) |  | 0.15  (0.03, 0.66) | 6.678 | 12.2  (9, 13) |
| **Nonspecific acute adverse events** | | | | | | | | | | | | | | | |
| **Intervention of different routes** | **Main results** | | |  | **Excluded placebo** | | |  | **Excluded single blinded and unblinded study** | | |  | **Excluded "0" study** | | |
|  | **OR,**  **95% CI** | **SUCRA (%)** | **Ranking, 95% CI** |  | **OR,**  **95% CI** | **SUCRA (%)** | **Ranking, 95% CI** |  | **OR,**  **95% CI** | **SUCRA (%)** | **Ranking, 95% CI** |  | **OR, 95% CI** | **SUCRA (%)** | **Ranking, 95% CI** |
| **NSAIDs, IM** | Reference | 75.72 | 4.2  (1, 8) |  | Reference | 77.92 | 3.6  (1, 7) |  | Reference | 69.58 | 4.3  (1, 8) |  | Reference | 70.73 | 3.3  (1, 6) |
| **NSAIDs, IV** | 0.64  (0.19, 2.01) | 63.03 | 5.8  (2, 9) |  | 0.63  (0.19, 1.99) | 64.33 | 5.3  (2, 9) |  | 1.06  (0.24, 4.23) | 72.69 | 4.0  (1, 8) |  | 0.97  (0.26, 3.59) | 71.08 | 3.3  (1, 6) |
| **NSAIDs, PR** | 0.02  (4.17E-05, 0.65) | 12.07 | 12.4  (6, 14) |  | 0.02  (4.31E-05, 0.80) | 12.8 | 11.5  (4, 13) |  | NA | NA | NA |  | NA | NA | NA |
| **NSAIDs, SU** | 0.39  (0.01, 6.22) | 48.39 | 7.7  (1, 14) |  | 0.40  (0.01, 6.26) | 51.21 | 6.9  (1, 12) |  | 0.40  (0.01, 7.31) | 43.17 | 7.3  (1, 12) |  | NA | NA | NA |
| **Opioids, IM** | 0.22  (0.11, 0.42) | 31.96 | 9.8  (6, 13) |  | 0.22  (0.11, 0.41) | 32.87 | 9.1  (5, 12) |  | 0.31  (0.17, 0.53) | 30.77 | 8.6  (5, 11) |  | 0.22  (0.11, 0.41) | 19.64 | 7.4  (5, 9) |
| **Opioids, IV** | 0.28  (0.10, 0.74) | 36.24 | 9.3  (6, 12) |  | 0.27  (0.10, 0.71) | 36.84 | 8.6  (6, 11) |  | 0.38  (0.10, 1.50) | 34.97 | 8.2  (5, 11) |  | 0.37  (0.12, 1.19) | 31.75 | 6.5  (4, 8) |
| **Opioids, SC** | 1.05  (0.10, 10.57) | 72.01 | 4.6  (1, 12) |  | 1.06  (0.10, 10.85) | 73.69 | 4.2  (1, 11) |  | NA | NA | NA |  | 4.19  (0.20, 2.35E+02) | 89.39 | 1.8  (1, 7) |
| **Paracetamol, IV** | 0.81  (0.26, 2.55) | 69.96 | 4.9  (1, 9) |  | 0.81  (0.26, 2.42) | 71.4 | 4.4  (1, 8) |  | 0.94  (0.24, 3.30) | 66.67 | 4.7  (1, 9) |  | 0.98  (0.29, 3.41) | 69.19 | 3.5  (1, 7) |
| **Paracetamol, PO** | 1.01  (1.99E-03, 1.04E+03) | 63.29 | 5.8  (1, 14) |  | 1.01  (1.23E-03, 6.73E+02) | 63.78 | 5.3  (1, 13) |  | 0.88  (8.66E-04, 8.79E+02) | 57.13 | 5.7  (1, 12) |  | NA | NA | NA |
| **Double Opioids, IM** | 0.01  (3.21E-04, 0.19) | 5.813 | 13.2  (11, 14) |  | 0.01  (2.60E-04, 0.18 ) | 5.831 | 12.3  (10, 13) |  | 0.02  (4.46E-04, 0.28) | 4.167 | 11.5  (9, 12) |  | 0.02  (4.16E-04, 0.26) | 0.846 | 8.9  (8, 9) |
| **Double Opioids, IV** | 0.34  (0.03, 3.28) | 45.64 | 8.1  (2, 13) |  | 0.33  (0.04, 2.82) | 45.26 | 7.6  (2, 12) |  | 0.55  (0.06, 4.31) | 49.67 | 6.5  (2, 11) |  | 0.51  (0.06, 5.51) | 47.27 | 5.2  (1, 8) |
| **NSAIDs, IV + Opioids, IV** | 0.41  (0.08, 1.73) | 48.4 | 7.7  (3, 12) |  | 0.40  (0.09, 1.74) | 49.75 | 7.0  (2, 11) |  | 0.63  (0.12, 2.97) | 53.2 | 6.1  (2, 10) |  | 0.59  (0.12, 2.93) | 50.11 | 5.0  (2, 8) |
| **NSAIDs, IV + Paracetamol, PO** | 1.01  (2.51E-03, 7.81E+02) | 63.13 | 5.8  (1, 14) |  | 1.11  (1.43E-03, 1.02E+03) | 64.32 | 5.3  (1, 13) |  | 0.89  (6.98E-04, 9.69E+02) | 57.42 | 5.7  (1, 12) |  | NA | NA | NA |
| **Placebo** | 1.14  (1.85E-03, 9.80E+02) | 64.35 | 5.6  (1, 14) |  | NA | NA | NA |  | 1.12  (1.91E-03, 1.38E+03) | 60.57 | 5.3  (1, 12) |  | NA | NA | NA |
| **Vomiting as an adverse event** | | | | | | | | | | | | | | | |
| **Intervention of different routes** | **Main results** | | |  | **Excluded placebo** | | |  | **Excluded single blinded and unblinded study** | | |  | **Excluded "0" study** | | |
|  | **OR,**  **95% CI** | **SUCRA (%)** | **Ranking, 95% CI** |  | **OR,**  **95% CI** | **SUCRA (%)** | **Ranking, 95% CI** |  | **OR,**  **95% CI** | **SUCRA (%)** | **Ranking, 95% CI** |  | **OR, 95% CI** | **SUCRA (%)** | **Ranking, 95% CI** |
| **NSAIDs, IM** | Reference | 64.46 | 6  (2, 11) |  | Reference | 66.59 | 5.3  (2, 10) |  | Reference | 66.51 | 5.4  (2, 10) |  | Reference | 66.07 | 4.1  (1, 9) |
| **NSAIDs, IV** | 0.88  (0.10, 7.84) | 62.54 | 6.2  (2, 11) |  | 0.87  (0.10, 8.87) | 64.25 | 5.6  (2, 10) |  | 1.26  (0.12, 12.89) | 71.85 | 4.7  (1, 9) |  | 0.77  (0.08, 6.55) | 57.75 | 4.8  (1, 9) |
| **NSAIDs, PR** | 0.79  (0.04, 13.09) | 56.55 | 7.1  (2, 13) |  | 0.78  (0.03, 14.96) | 58.34 | 6.4  (2, 12) |  | 0.42  (0.01, 13.98) | 46.02 | 8.0  (2, 14) |  | 0.61  (0.02, 17.49) | 48.29 | 5.7  (1, 10) |
| **NSAIDs, SU** | 1.00  (0.02, 57.16) | 58.78 | 6.8  (1, 15) |  | 0.92  (0.02, 50.14) | 59.52 | 6.3  (1, 14) |  | 1.02  (0.02, 50.77) | 60.89 | 6.1  (1, 14) |  | NA | NA | NA |
| **Opioids, IM** | 0.45  (0.13, 1.69) | 43.47 | 8.9  (3, 14) |  | 0.43  (0.11, 1.72) | 44.6 | 8.2  (3, 13) |  | 0.52  (0.13, 2.07) | 48.5 | 7.7  (2, 13) |  | 0.59  (0.16, 2.04) | 46.04 | 5.9  (1, 10) |
| **Opioids, IV** | 0.23  (0.04, 1.38) | 25.92 | 11.4  (8, 14) |  | 0.22  (0.03, 1.46) | 27.52 | 10.4  (6, 13) |  | 0.26  (0.03, 1.76) | 31.49 | 9.9  (6, 13) |  | 0.35  (0.05, 2.26) | 27.69 | 7.5  (4, 10) |
| **Opioids, PR** | 0.77  (0.03, 23.51) | 56.35 | 7.1  (1, 14) |  | 0.86  (0.02, 30.50) | 58.87 | 6.3  (1, 13) |  | 0.43  (0.01, 22.41) | 47.22 | 7.9  (1, 14) |  | 0.67  (0.01, 27.42) | 51.16 | 5.4  (1, 10) |
| **Opioids, SC** | 5.35  (0.05, 4.13E+03) | 80.95 | 3.7  (1, 13) |  | 5.74  (0.05, 3.42E+03) | 81.46 | 3.4  (1, 12) |  | NA | NA | NA |  | NA | NA | NA |
| **Opioids, SU** | 0.05  (3.46E-03, 0.67) | 9.067 | 13.7  (10, 15) |  | 0.05  (2.80E-03, 0.62) | 9.579 | 12.8  (9, 14) |  | 0.06  (3.51E-03, 0.94) | 12.1 | 12.4  (8, 14) |  | 0.29  (0.01, 5.26) | 29.08 | 7.4  (2, 10) |
| **Paracetamol, IV** | 0.44  (0.07, 2.44) | 42.96 | 9  (4, 13) |  | 0.48  (0.07, 3.30) | 48.42 | 7.7  (3, 12) |  | 0.52  (0.06, 3.93) | 50.34 | 7.5  (3, 12) |  | 0.42  (0.05, 2.79) | 34.86 | 6.9  (3, 10) |
| **Paracetamol, PO** | 0.97  (1.38E-03, 1.04E+03) | 56.18 | 7.1  (1, 15) |  | 0.80  (1.29E-03, 1.05E+03) | 56.03 | 6.7  (1, 14) |  | 1.03  (1.32E-03, 9.25E+02) | 58.69 | 6.4  (1, 14) |  | NA | NA | NA |
| **Double Opioids, IM** | 0.05  (7.19E-05, 3.19 ) | 16.75 | 12.7  (4, 15) |  | 0.05  (7.15E-05, 3.85) | 18.33 | 11.6  (3, 14) |  | 0.07  (1.56E-04, 4.41) | 21.58 | 11.2  (3, 14) |  | NA | NA | NA |
| **NSAIDs, IV + Opioids, IV** | 0.51  (0.04, 6.48) | 48.01 | 8.3  (2, 14) |  | 0.50  (0.03, 7.96) | 49.44 | 7.6  (2, 13) |  | 0.48  (0.02, 17.02) | 49.58 | 7.6  (1, 14) |  | 0.79  (0.04, 16.58) | 57.22 | 4.9  (1, 10) |
| **NSAIDs, IM + Paracetamol, PO** | 0.92  (9.41E-04, 1.02E+03) | 55.43 | 7.2  (1, 15) |  | 0.94  (1.80E-03, 1.39E+03) | 57.04 | 6.6  (1, 14) |  | 0.99  (1.11E-03, 8.82E+02) | 58.52 | 6.4  (1, 14) |  | NA | NA | NA |
| **Placebo** | 1.57  (0.14, 20.24) | 72.58 | 4.8  (1, 11) |  | NA | NA | NA |  | 1.85  (0.14, 27.35) | 76.71 | 4.0  (1, 10) |  | 2.19  (0.14, 41.21) | 81.84 | 2.6  (1, 8) |

**Note:** For the outcome of failure of complete relief at 30 min, the sensitivity analyses based on the excluded single blinded and unblinded study, the overall network was divided into two small networks, one of which was a, indicates the independent cracked small network (NSAIDs, IM vs. NSAIDs, SU vs. Opioids, IM vs. Placebo), and the other one was b, indicates the independent cracked small network (NSAIDs, IV vs. NSAIDs, PR vs. Opioids, IV vs. Opioids, SC vs. Paracetamol, IV vs. NSAIDs, IV + Opioids, IV). Due to few study were included in failure of ≥50% pain relief at 30 min, it did not include sensitive indicators, such as placebo, single blinded and unblinded study, or "0" study, the sensitivity analyses were disregarded. NSAIDs: Nonsteroidal anti-inflammatory drugs, IM: Intramuscular route, IV: Intravenous route, PO: Per oral route, PR: Per rectal route, SC: Subcutaneous route, SU: Sublingual route, MD: Mean difference, OR: odds ratios, CrIs: Credible intervals, SUCRA: Surface under the cumulative ranking, NA: Not available.

**Supplement Figure 1.** **The summary plot for risk of bias.**

**
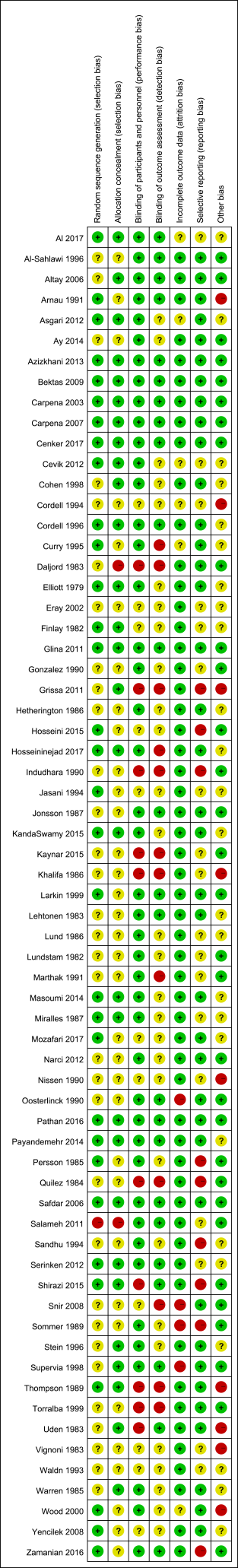
**

**Supplement Figure 2.** **The network of eligible studies with different interventions for failure of complete relief at 30 min (A), failure of ≥50% pain relief at 30 min (B), need for rescue analgesia (C), nonspecific acute adverse events (D), and vomiting as an adverse event (E) from first stage.**

**
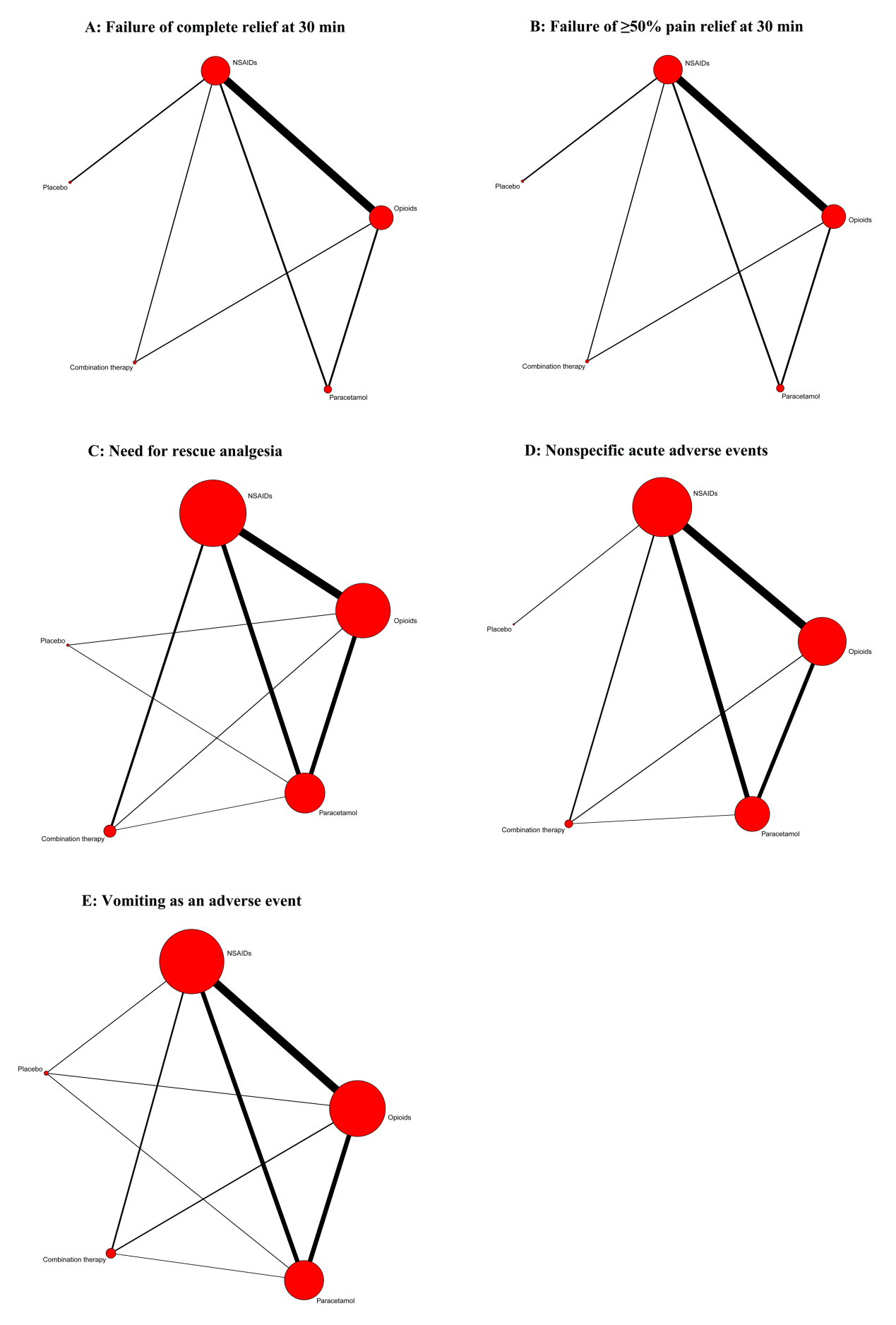
**

**Note:** The node sizes correspond to the number of accumulated sample size that investigated the treatments. Directly comparable treatments are linked with a line, and the thickness of the line corresponds to the sum of the sample size in each pairwise treatment comparison. NSAIDs: Nonsteroidal anti-inflammatory drugs.

**Supplement Figure 3. The results of loop consistency for pain variance at 30 min (A), failure of complete relief at 30 min (B), failure of ≥50% pain relief at 30 min (C), need for rescue analgesia (D), nonspecific acute adverse events (E), and vomiting as an adverse event (F) from first stage.**

**
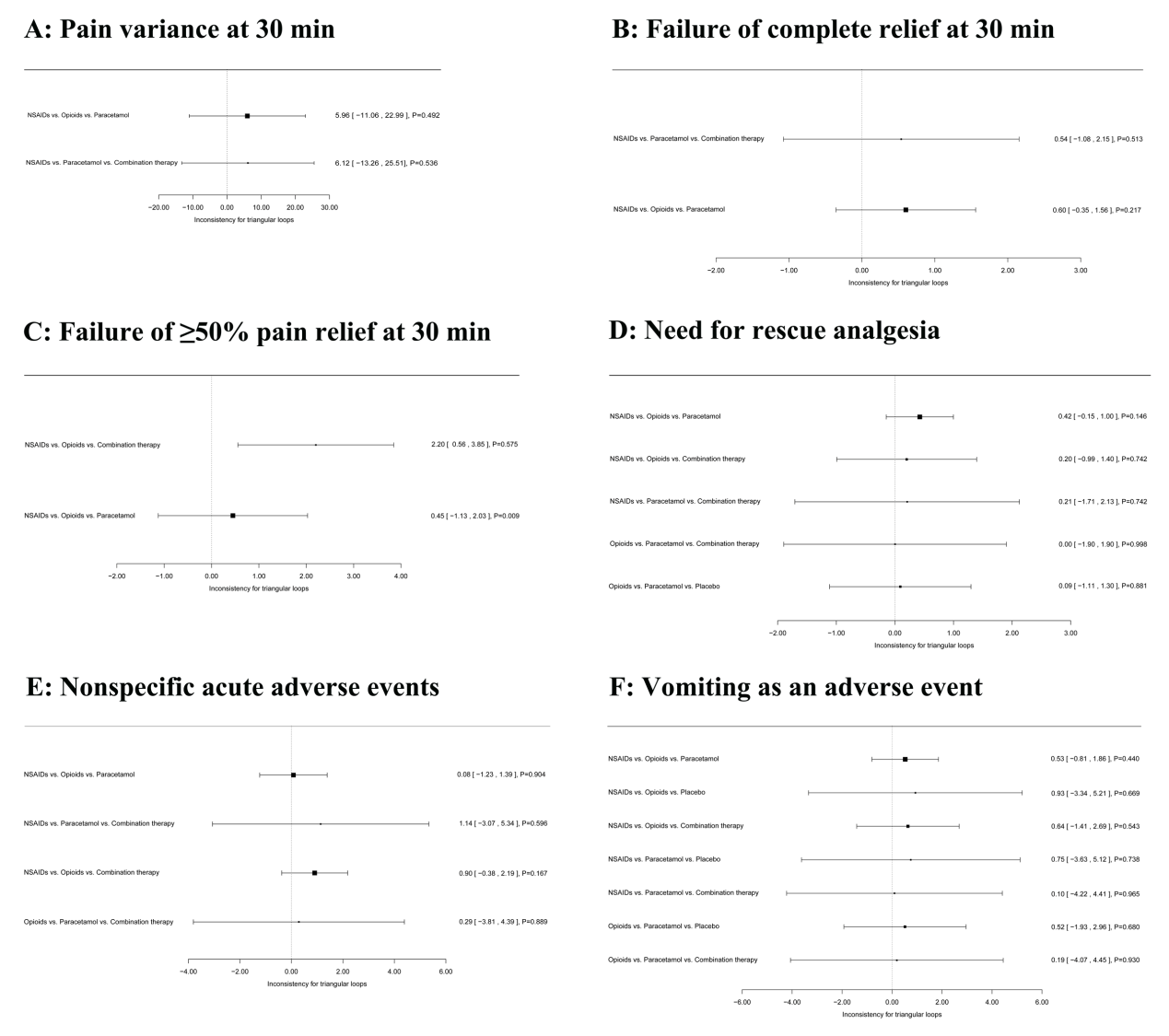
**

**Supplement Figure 4A. The results of direct comparison with NSAIDs, opioids, paracetamol, combination therapy and placebo with different routes for pain variance at 30 min from second stage.**

**
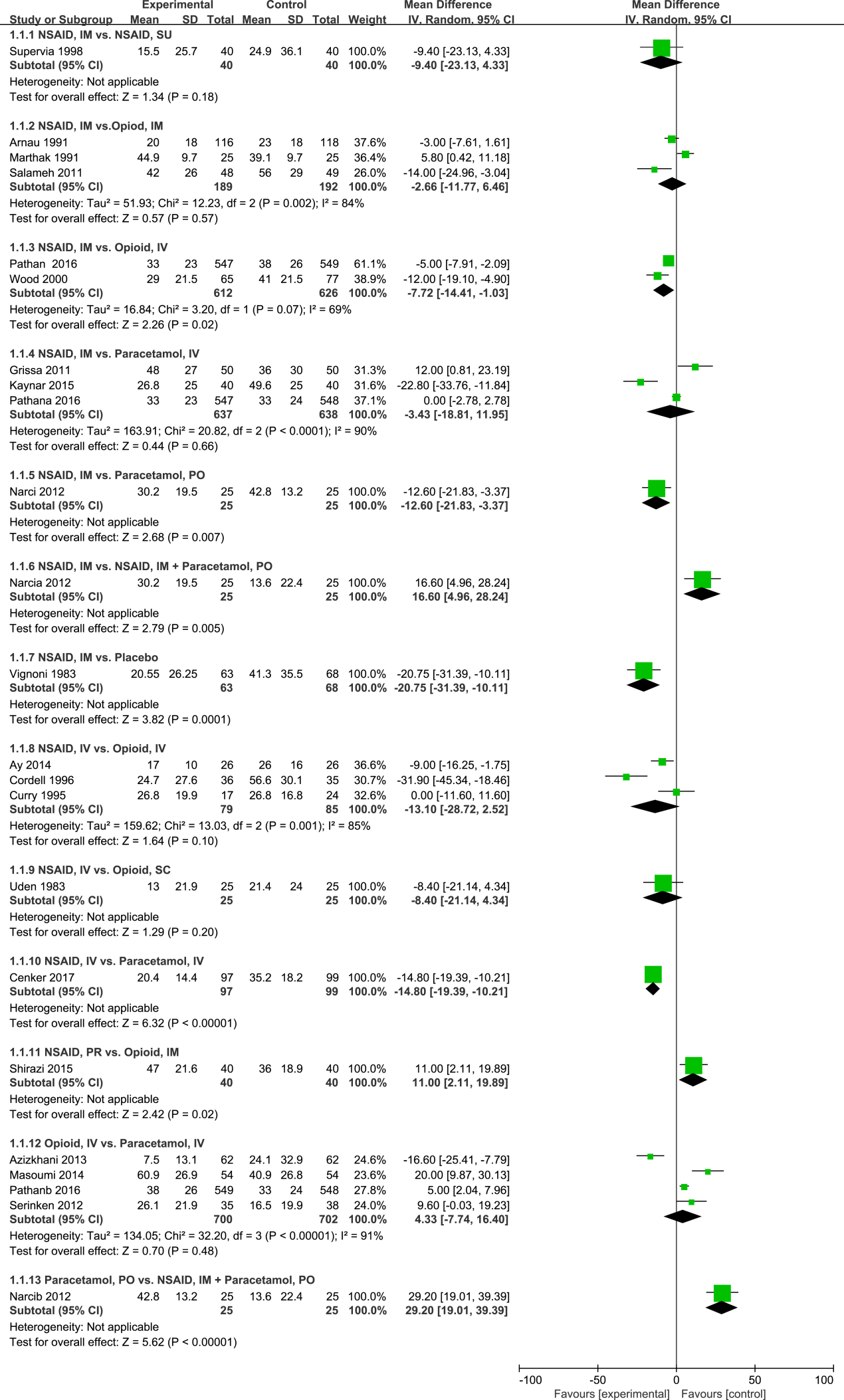
**

**Note:** NSAIDs: Nonsteroidal anti-inflammatory drugs, IM: Intramuscular route, IV: Intravenous route, PO: Per oral route, PR: Per rectal route, SC: Subcutaneous route, SU: Sublingual route.

**Supplement Figure 4B. The results of direct comparison with NSAIDs, opioids, paracetamol, combination therapy and placebo with different routes for failure of complete relief at 30 min from second stage.**

**
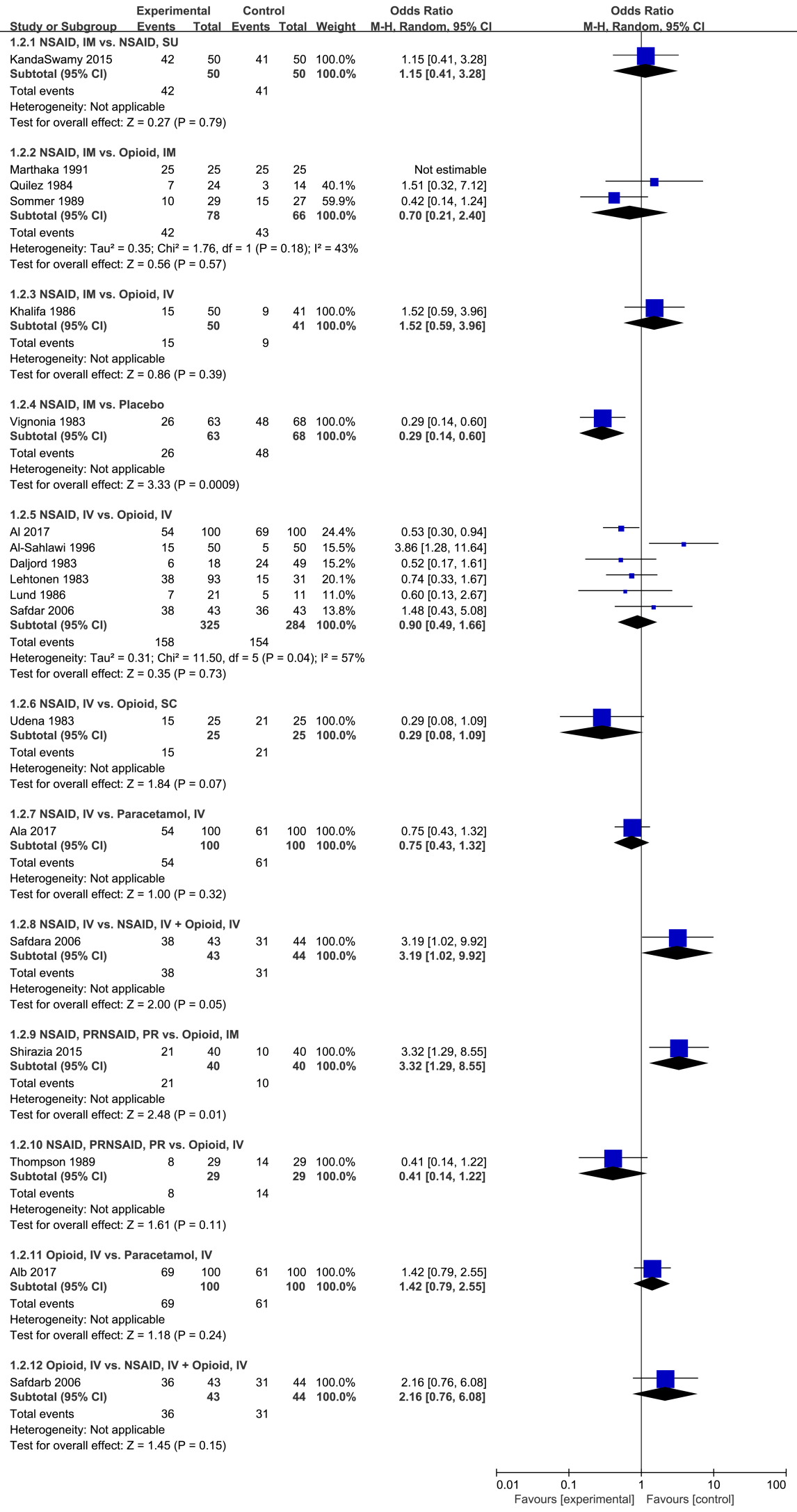
**

**Note:** NSAIDs: Nonsteroidal anti-inflammatory drugs, IM: Intramuscular route, IV: Intravenous route, PO: Per oral route, PR: Per rectal route, SC: Subcutaneous route, SU: Sublingual route.

**Supplement Figure 4C. The results of direct comparison with NSAIDs, opioids, paracetamol, combination therapy and placebo with different routes for failure of ≥50% pain relief at 30 min from second stage.**

**
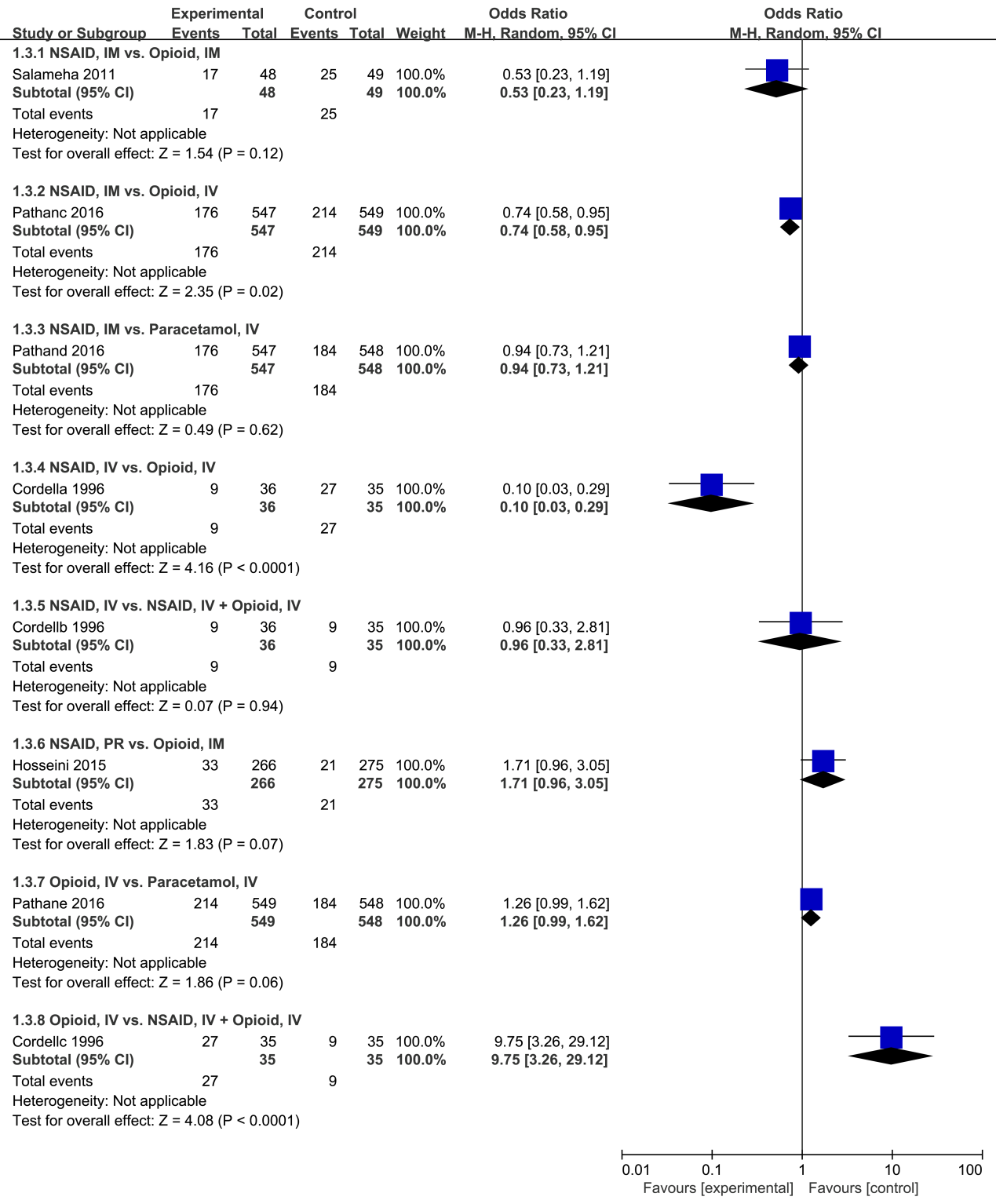
**

**Note:** NSAIDs: Nonsteroidal anti-inflammatory drugs, IM: Intramuscular route, IV: Intravenous route, PO: Per oral route, PR: Per rectal route, SC: Subcutaneous route, SU: Sublingual route.

**Supplement Figure 4D. The results of direct comparison with NSAIDs, opioids, paracetamol, combination therapy and placebo with different routes for need for rescue analgesia from second stage.**

**
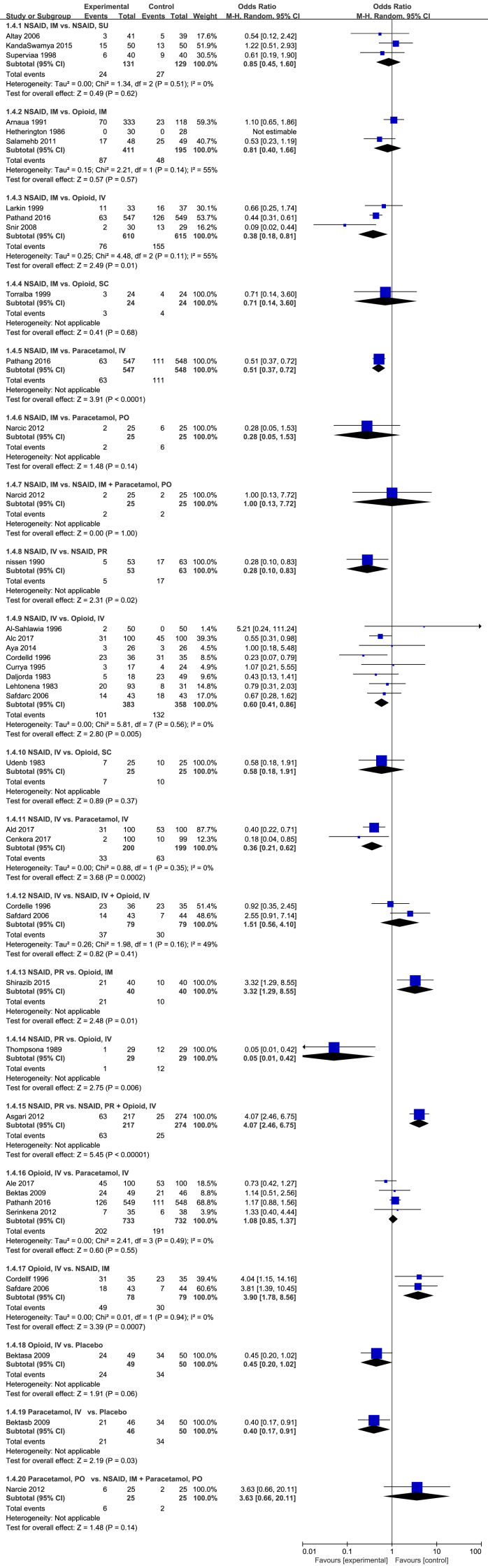
**

**Note:** NSAIDs: Nonsteroidal anti-inflammatory drugs, IM: Intramuscular route, IV: Intravenous route, PO: Per oral route, PR: Per rectal route, SC: Subcutaneous route, SU: Sublingual route.

**Supplement Figure 4E. The results of direct comparison with NSAIDs, opioids, paracetamol, combination therapy and placebo with different routes for nonspecific acute adverse events from second stage.**

**
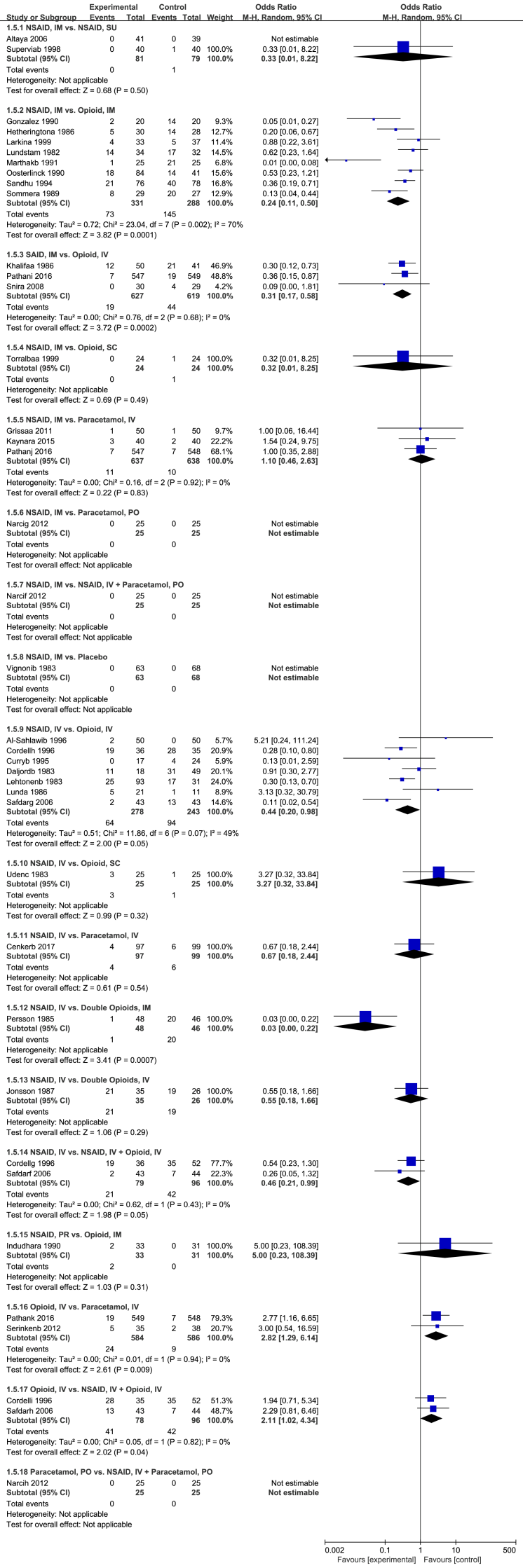
**

**Note:** NSAIDs: Nonsteroidal anti-inflammatory drugs, IM: Intramuscular route, IV: Intravenous route, PO: Per oral route, PR: Per rectal route, SC: Subcutaneous route, SU: Sublingual route.

**Supplement Figure 4F. The results of direct comparison with NSAIDs, opioids, paracetamol, combination therapy and placebo with different routes for vomiting as an adverse event from second stage.**

**
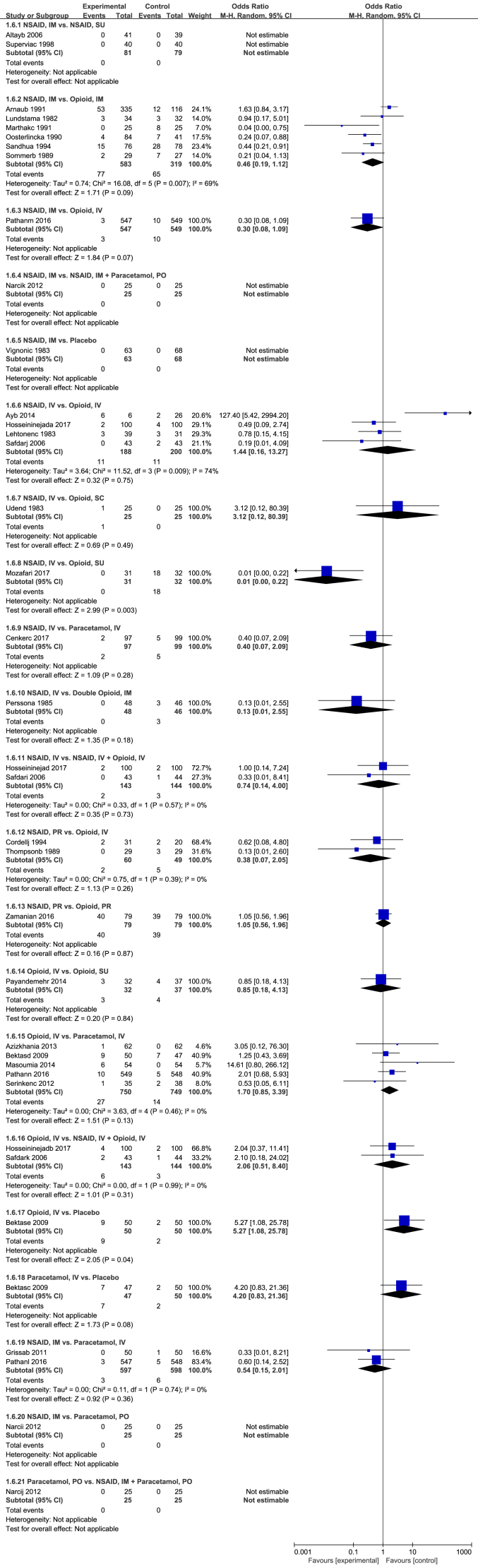
**

**Note:** NSAIDs: Nonsteroidal anti-inflammatory drugs, IM: Intramuscular route, IV: Intravenous route, PO: Per oral route, PR: Per rectal route, SC: Subcutaneous route, SU: Sublingual route.

**Supplement Figure 5.** **The network of eligible studies with NSAIDs, opioids, paracetamol, combination therapy and placebo with different routes for failure of complete relief at 30 min (A), failure of ≥50% pain relief at 30 min (B), need for rescue analgesia (C), nonspecific acute adverse events (D), and vomiting as an adverse event (E) from second stage.**

**
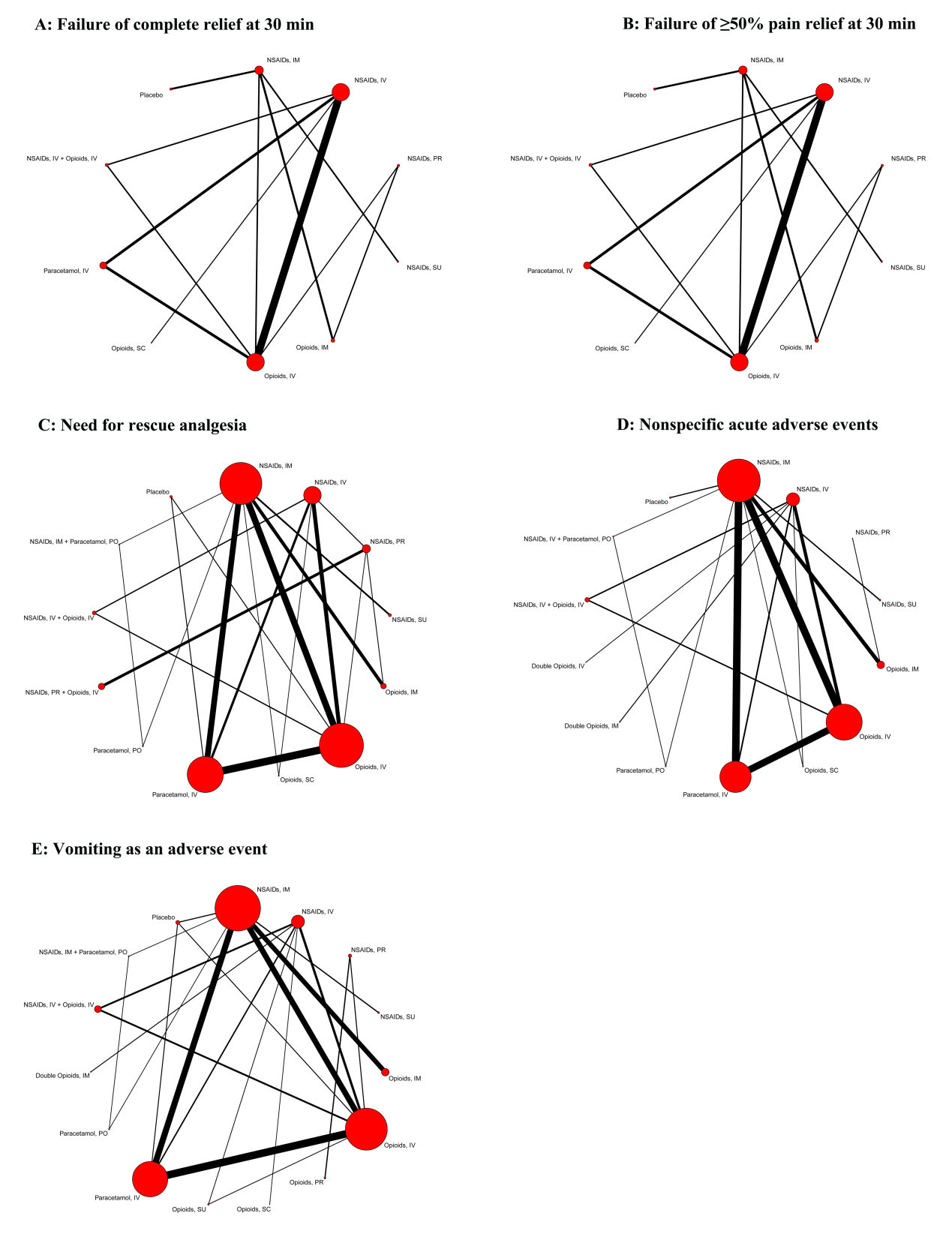
**

**Note:** The node sizes correspond to the number of trials that investigated the treatments. Directly comparable treatments are linked with a line, and the thickness of the line corresponds to the sum of the sample size in each pairwise treatment comparison. NSAIDs: Nonsteroidal anti-inflammatory drugs, IM: Intramuscular route, IV: Intravenous route, PO: Per oral route, PR: Per rectal route, SC: Subcutaneous route, SU: Sublingual route.

**Supplement Figure 6.** **The results of loop consistency for pain variance at 30 min (A), failure of complete relief at 30 min (B), failure of ≥50% pain relief at 30 min (C), need for rescue analgesia (D), nonspecific acute adverse events (E), and vomiting as an adverse event (F) from second stage.**

**
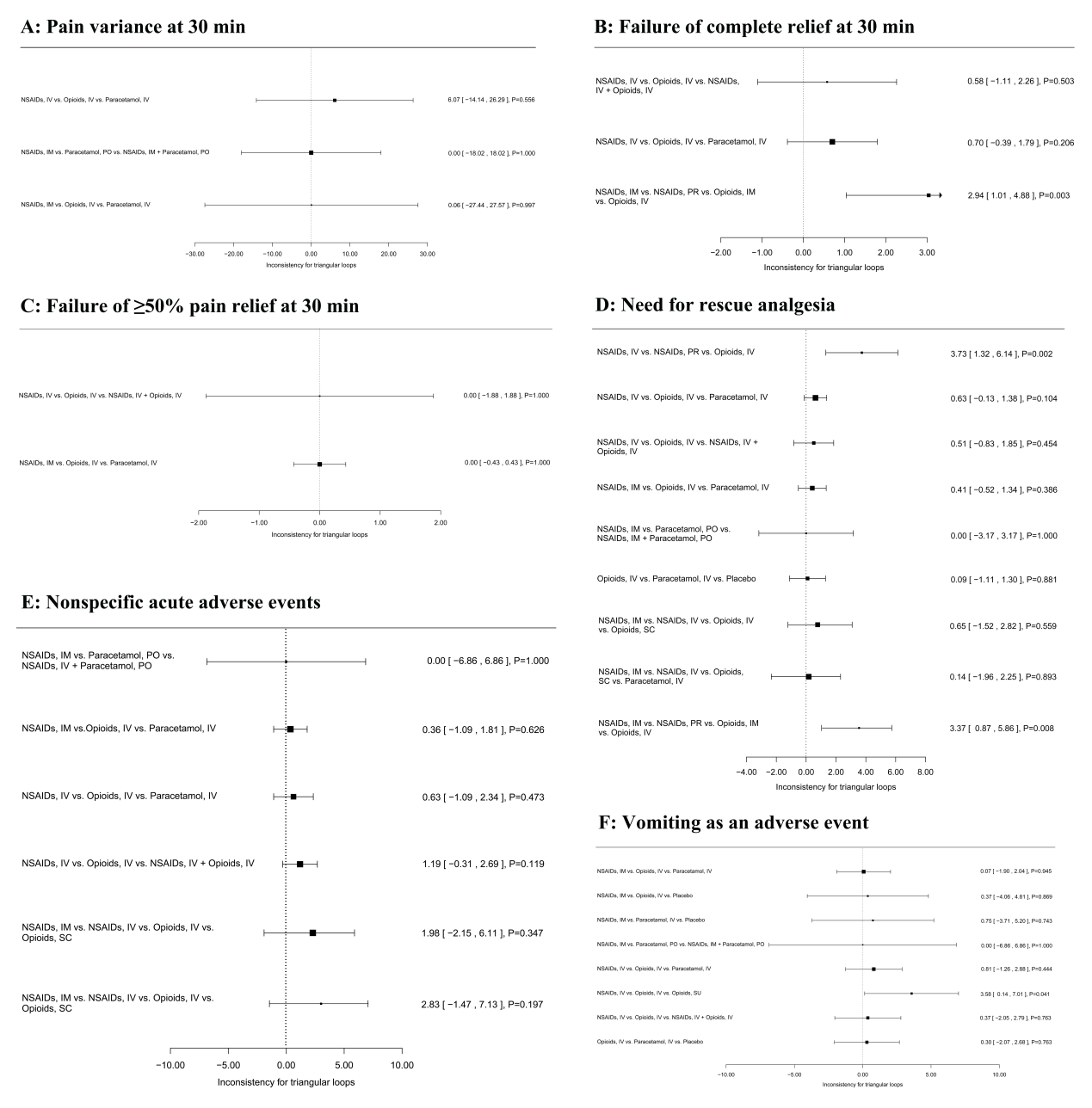
**

**Supplement Figure 7. Forest plots for effect sizes compared with NSAIDs with intramuscular route from second stage.**

**
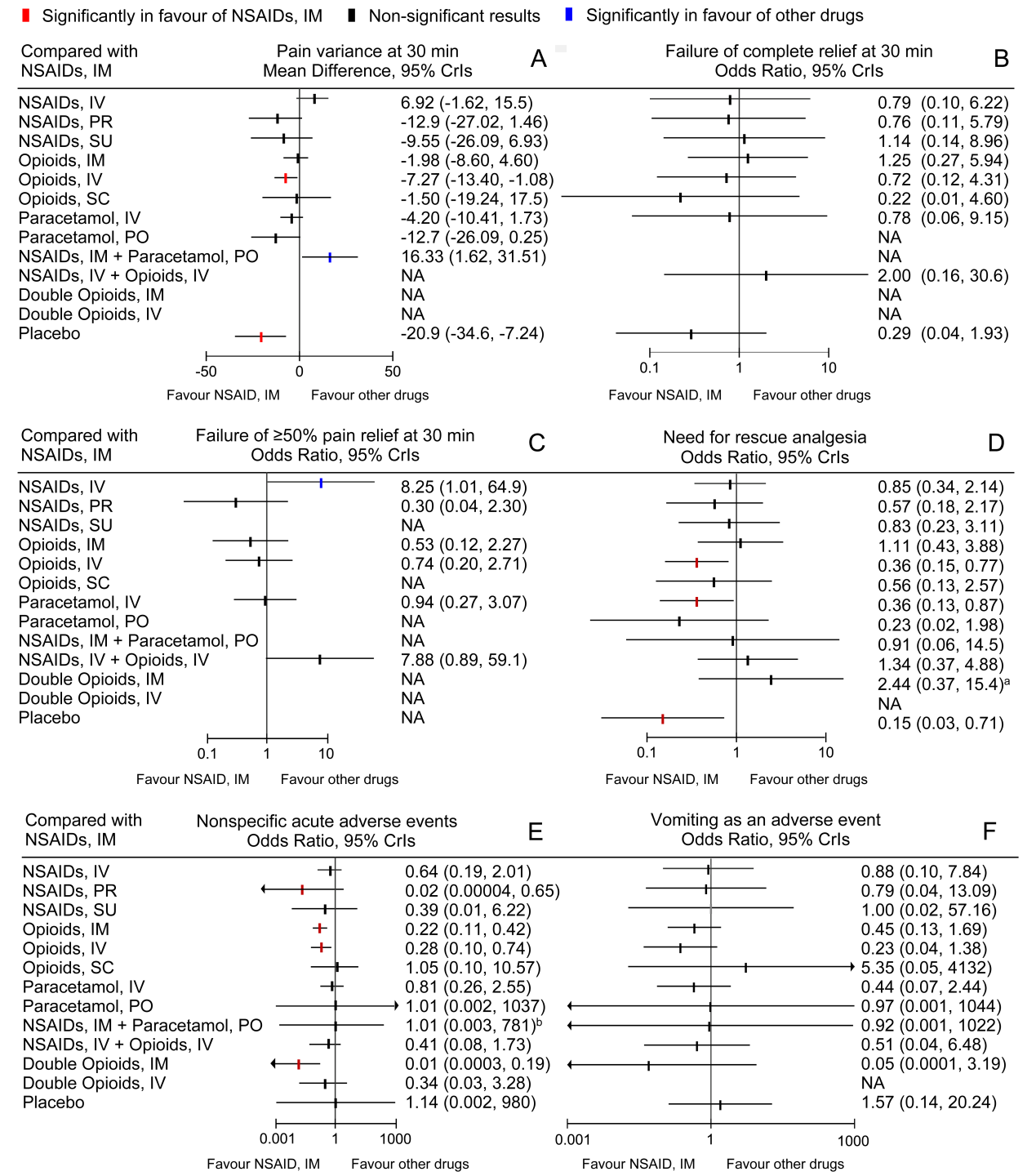
**

**Note:** The result of “a” indicates the comparison between nonsteroidal anti-inflammatory drugs with intramuscular route (NSAIDs, IM) and nonsteroidal anti-inflammatory drugs with per rectal route plus opioids with intravenous route (NSAIDs, PR + Opioids, IV), and “b” indicates the comparison between nonsteroidal anti-inflammatory drugs with intramuscular route (NSAIDs, IM) and nonsteroidal anti-inflammatory drugs with intravenous rout plus paracetamol with per oral route (NSAIDs, IV + Paracetamol, PO). NSAIDs: Nonsteroidal anti-inflammatory drugs, IM: Intramuscular route, IV: Intravenous route, PO: Per oral route, PR: Per rectal route, SC: Subcutaneous route, SU: Sublingual route.

**Supplement Figure 8. Ranking for NSAIDs, opioids, paracetamol, combination therapy and placebo with different routes for pain variance at 30 min and nonspecific acute adverse events (A)/vomiting as an adverse event (B) in network meta-analyses from second stage.**

**
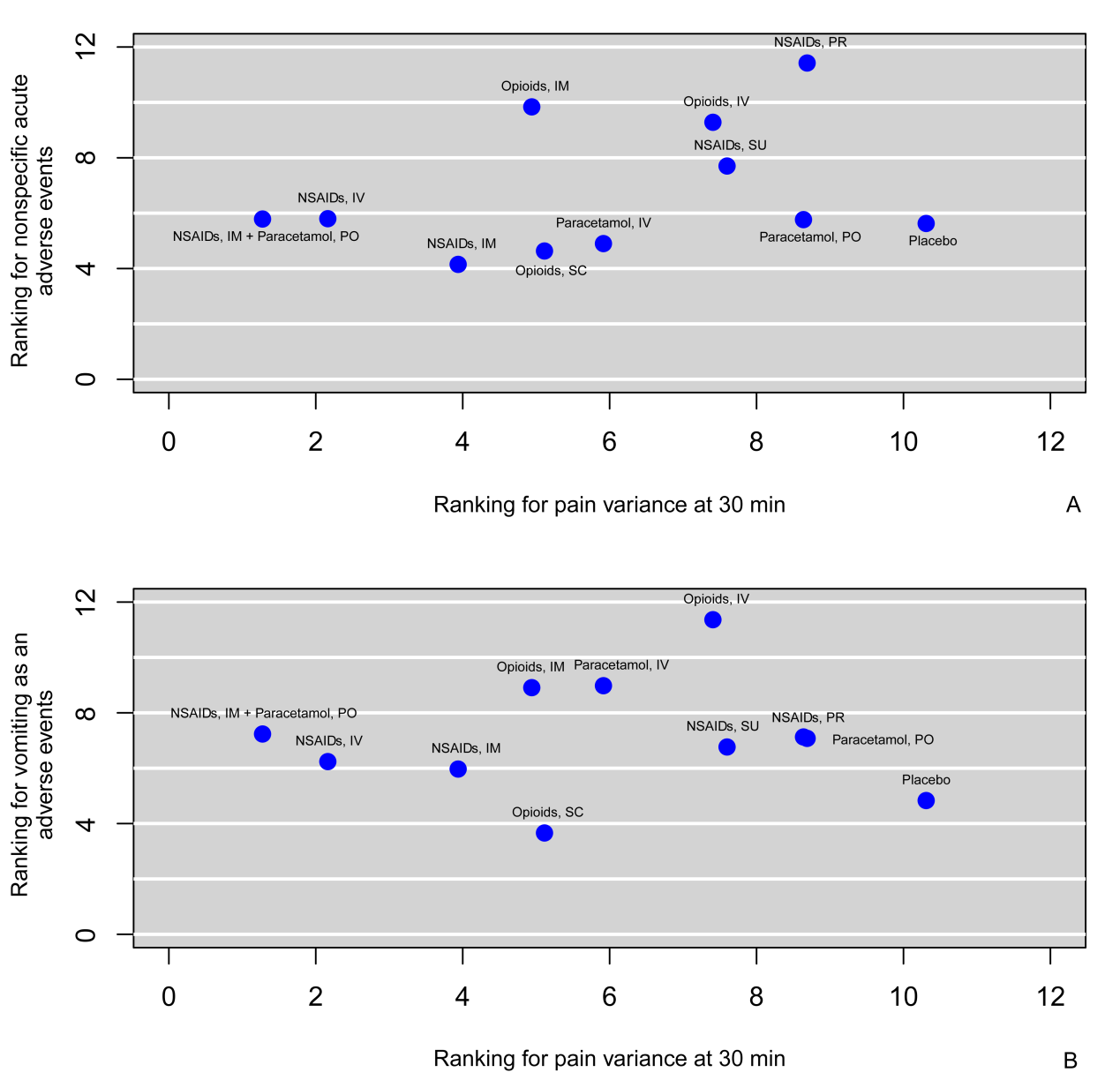
**

**Note:** All active drugs and placebo for all outcomes were ranked according to their probability of optimal efficacy or safety. In ranking order in the scatter plot, from best to worst, the lower rank demonstrate better effects or safer. The best intervention is in the lower left corner, while the worst is in the upper right corner. Due to a lack of “NSAIDs, IM + Paracetamol, PO” in nonspecific acute adverse events, the ranking result of nonspecific acute adverse events in the part A, it were replaced by “NSAIDs, IV + Paracetamol, PO”. NSAIDs: Nonsteroidal anti-inflammatory drugs, IM: Intramuscular route, IV: Intravenous route, PO: Per oral route, PR: Per rectal route, SC: Subcutaneous route, SU: Sublingual route.

**Supplement Figure 9.** **The comparison-adjusted funnel plots with NSAIDs, opioids, paracetamol, combination therapy and placebo with different routes for pain variance at 30 min (A), failure of complete relief at 30 min (B), failure of ≥50% pain relief at 30 min (C), need for rescue analgesia (D), nonspecific acute adverse events (E), and vomiting as an adverse event (F) from second stage.**

**
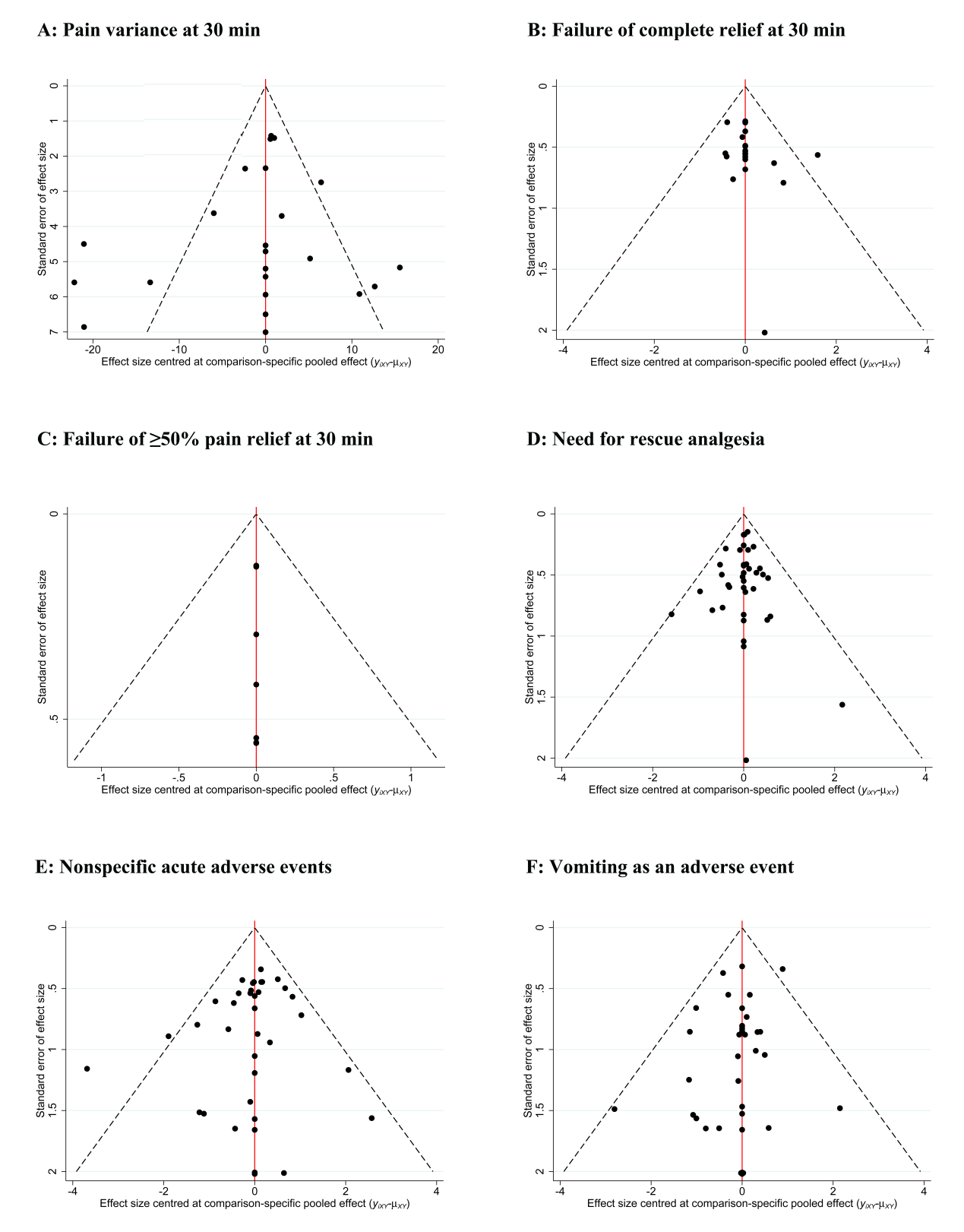
**

**Note:** In the comparison-adjusted funnel plot the horizontal axis shows the difference of each i-study's estimate y_iXY from the summary effect for the respective cpomparison (y_iXY-mu_XY), while the vertical axis presents a measure of dispersion of y_iXY. In the absence of small-study effects all studies are expected to lie symmetrically around the zero line of the comparison-adjusted funnel plot. NSAIDs: Nonsteroidal anti-inflammatory drugs, IM: Intramuscular route, IV: Intravenous route, PO: Per oral route, PR: Per rectal route, SC: Subcutaneous route, SU: Sublingual route.

**Supplement Figure 10.** **Forest plots for effect sizes with different drug branches and routes for pain variance at 30 min from third stage.**

**
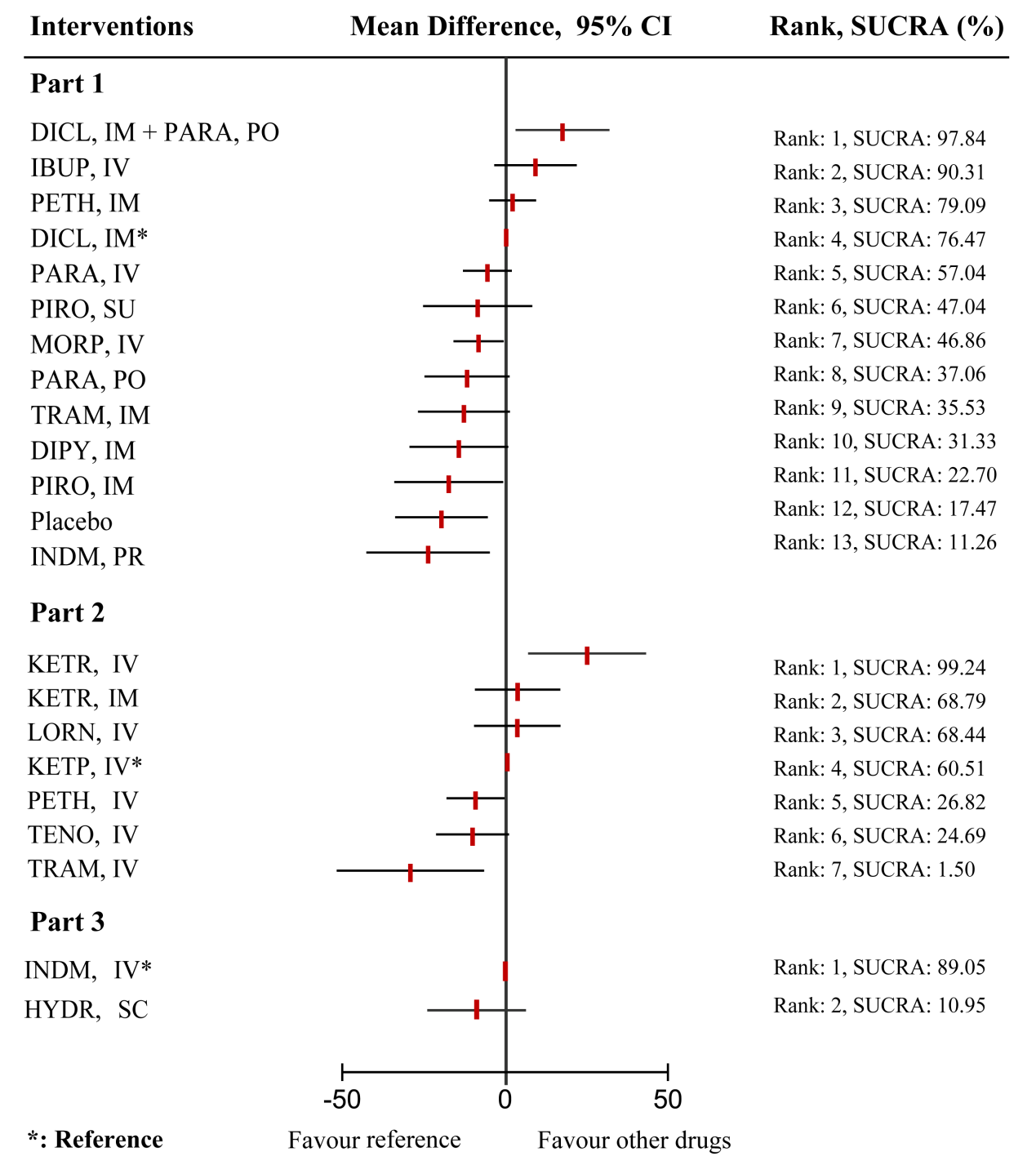
**

**Note:** IM: Intramuscular route, IV: Intravenous route, PO: Per oral route, PR: Per rectal route, SC: Subcutaneous route, SU: Sublingual route. DICL: Diclofenac, DIPY: Dipyrone, HYDR: Hydromorphine chloride-atropine, IBUP: Ibuprofen, INDM: Indomethacin, KETP: Ketoprofen, KETR: Ketorolac, LORN: Lornoxicam, MORP: Morphine, PARA: Paracetamol, PETH: Pethidine, PIRO: Piroxicam, TENO: Tenoxicam, TRAM: Tramadol.

**Supplement Figure 11.** **Integrated information plots with different drug branches and routes for failure of complete relief at 30 min from third stage.**

**
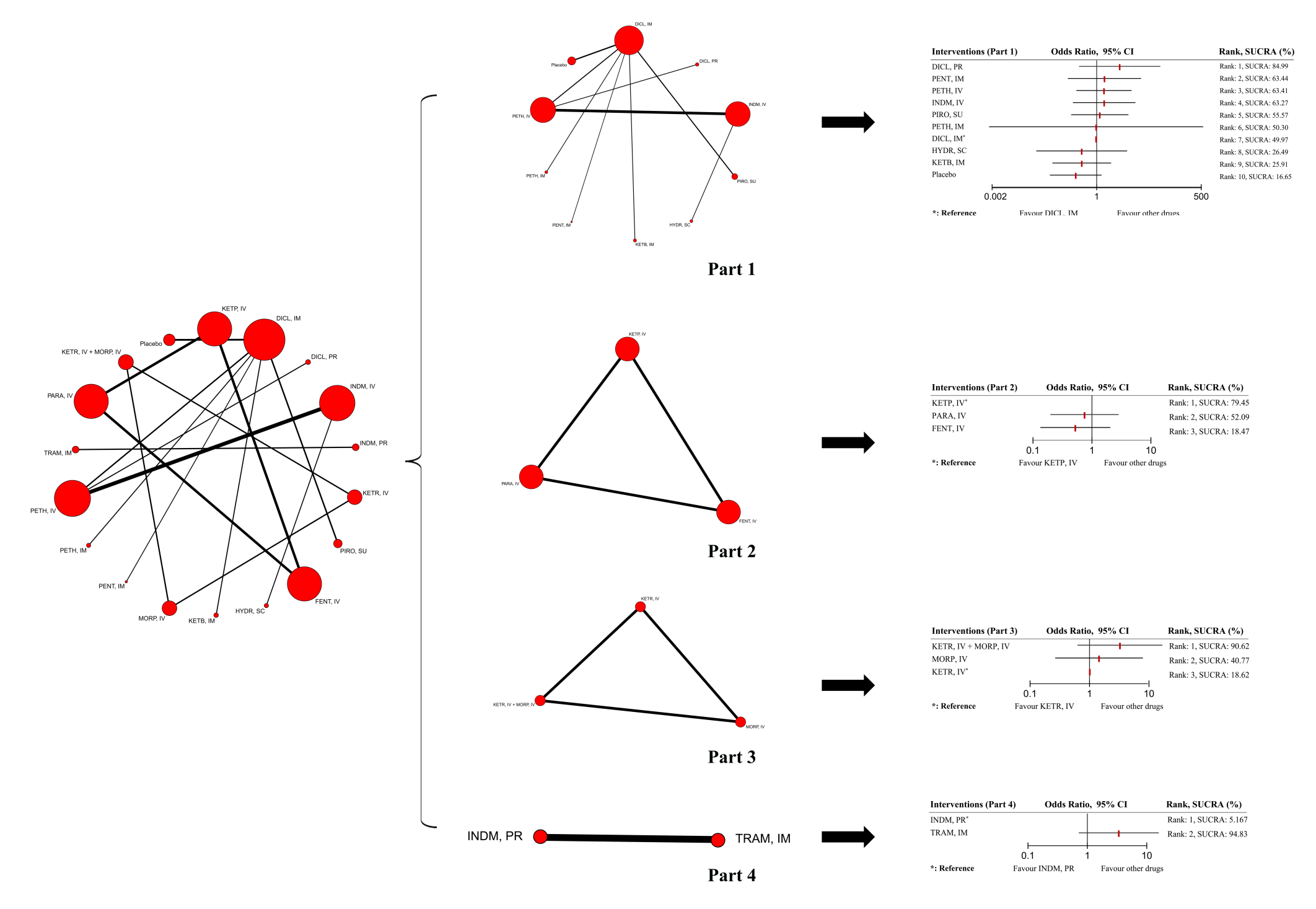
**

**Note:** The integrated information plot, including different drug branches and different routes, is composed of rude global network plot, natural punitive connecting network plots, forest plots, ranking, and their SUCRA values from network meta-analysis for failure of complete relief at 30 min in third stage. In rude global network plot, natural punitive connecting network plots, the node sizes correspond to the number of accumulated sample size that investigated the treatments. Directly comparable treatments are linked with a line, and the thickness of the line corresponds to the sum of the sample size in each pairwise treatment comparison. SUCRA: Surface under the cumulative ranking, IM: Intramuscular route, IV: Intravenous route, PR: Per rectal route, SC: Subcutaneous route, SU: Sublingual route. DICL: Diclofenac, FENT: Fentany, HYDR: Hydromorphine, INDM: Indomethacin, KETB: Ketogan, KETP: Ketoprofen, PARA: Paracetamol, PETH: Pethidine, PENT: pentazoxine, PIRO: Piroxicam.

**Supplement Figure 12.** **Integrated information plots with different drug branches and routes for failure of ≥50% pain relief at 30 min from third stage.**

**
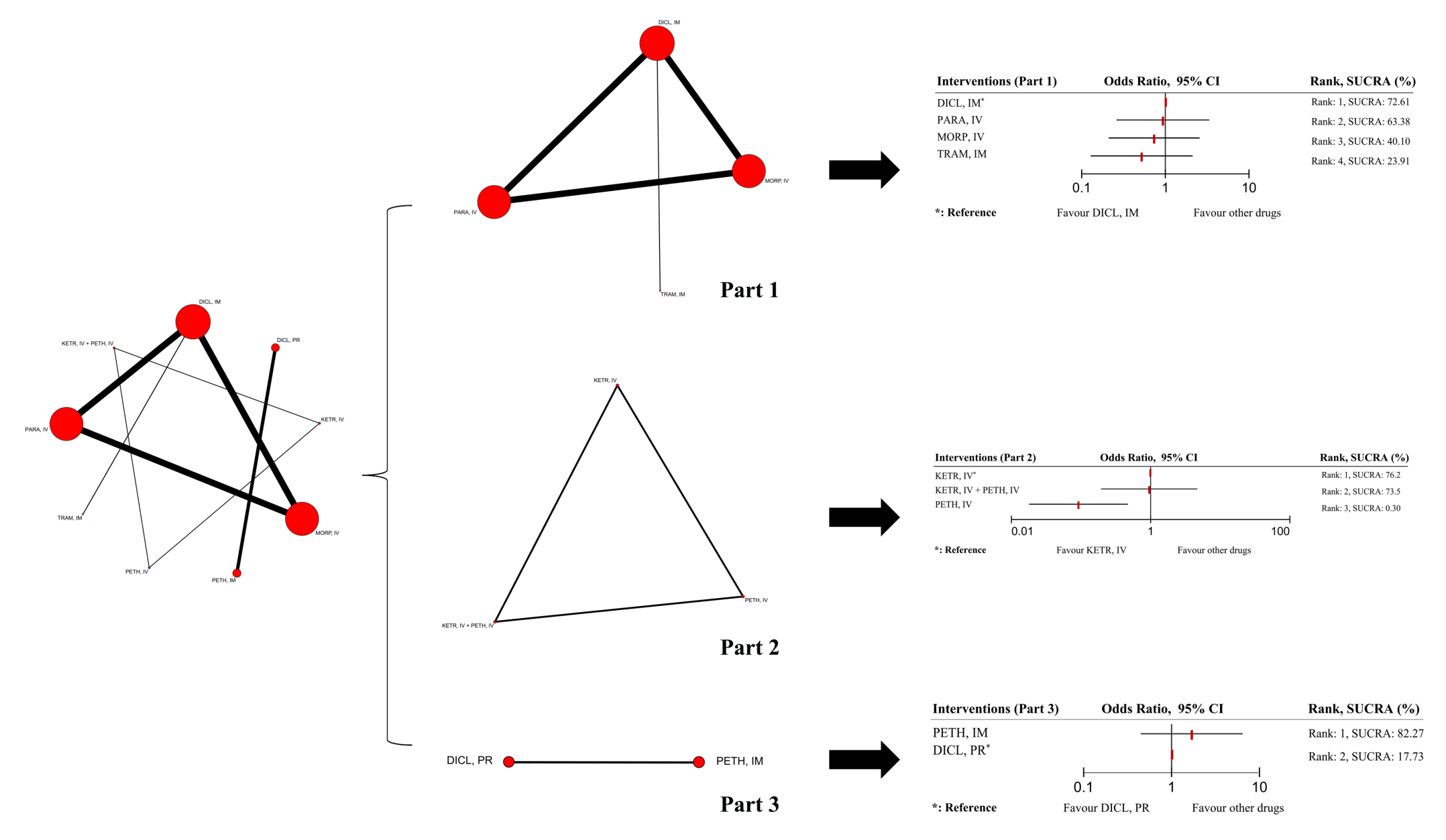
**

**Note:** The integrated information plot, including different drug branches and different routes, is composed of rude global network plot, natural punitive connecting network plots, forest plots, ranking, and their SUCRA values from network meta-analysis for failure of ≥50% pain relief at 30 min in third stage. In rude global network plot, natural punitive connecting network plots, the node sizes correspond to the number of accumulated sample size that investigated the treatments. Directly comparable treatments are linked with a line, and the thickness of the line corresponds to the sum of the sample size in each pairwise treatment comparison. SUCRA: Surface under the cumulative ranking, IM: Intramuscular route, IV: Intravenous route, PR: Per rectal route. DICL: Diclofenac, KETR: Ketorolac, MORP: Morphine, PARA: Paracetamol, PETH: Pethidine, TRAM: Tramadol.

**Supplement Figure 13.** **Integrated information plots with different drug branches and routes for need for rescue analgesia from third stage.**

**
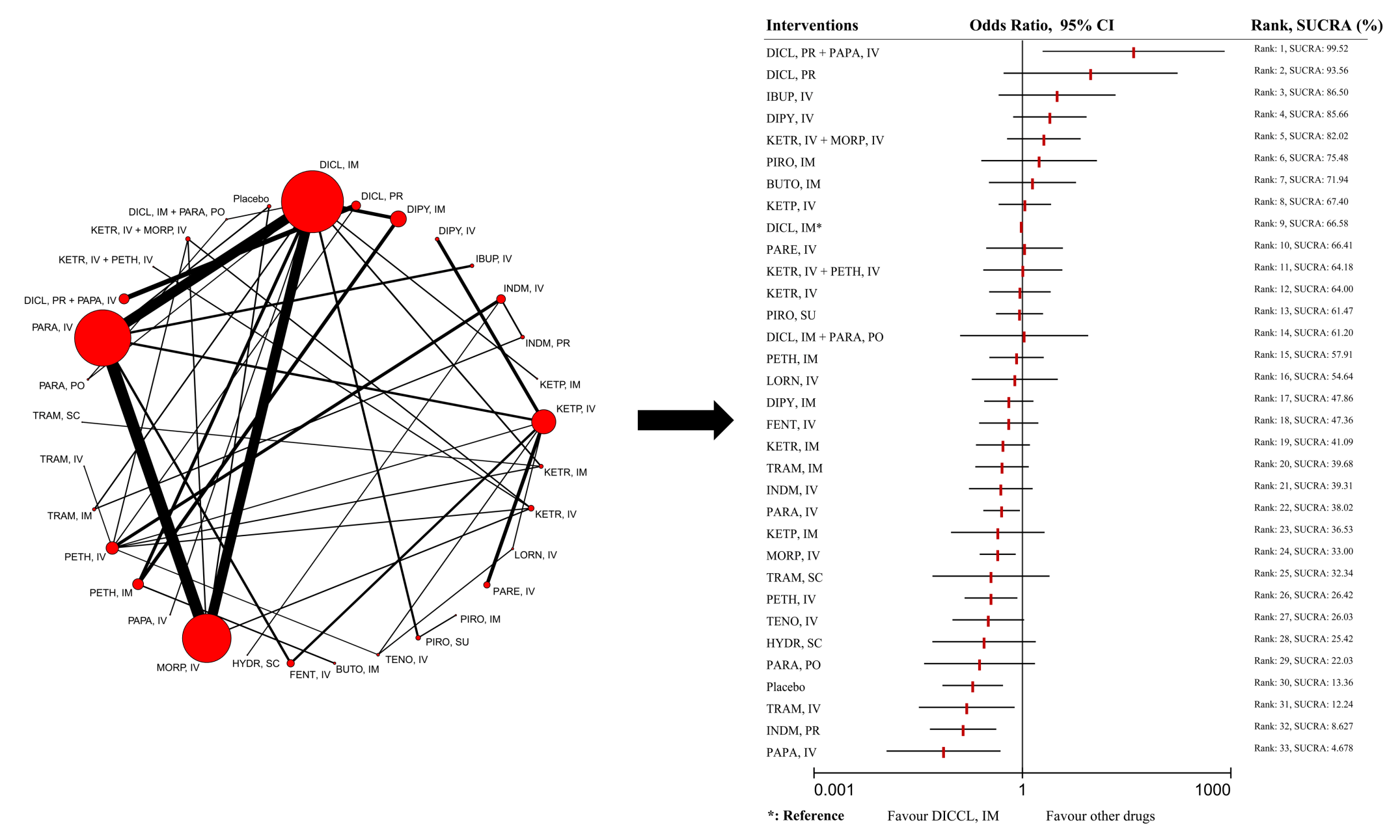
**

**Note:** The integrated information plot, including different drug branches and different routes, is composed of global network plot, forest plots, ranking, and their SUCRA values from network meta-analysis for need for rescue analgesia in third stage. In global network plot, the node sizes correspond to the number of accumulated sample size that investigated the treatments. Directly comparable treatments are linked with a line, and the thickness of the line corresponds to the sum of the sample size in each pairwise treatment comparison. SUCRA: Surface under the cumulative ranking, IM: Intramuscular route, IV: Intravenous route, PO: Per oral route, PR: Per rectal route, SC: Subcutaneous route, SU: Sublingual route. BUTO: Butorphanol, DICL: Diclofenac, DIPY: Dipyrone, HYDR: Hydromorphine, IBUP: Ibuprofen, INDM: Indomethacin, KETP: Ketoprofen, KETR: Ketorolac, LORN: Lornoxicam, MORP: Morphine, PAPA: Papaverine, PARA: Paracetamol, PARE: Parecoxib, PETH: Pethidine, PENT: Pentazoxine, PIRO: Piroxicam, TENO: Tenoxicam, TRAM: Tramadol.

**Supplement Figure 14.** **Integrated information plots with different drug branches and routes for nonspecific acute adverse events from third stage.**

**
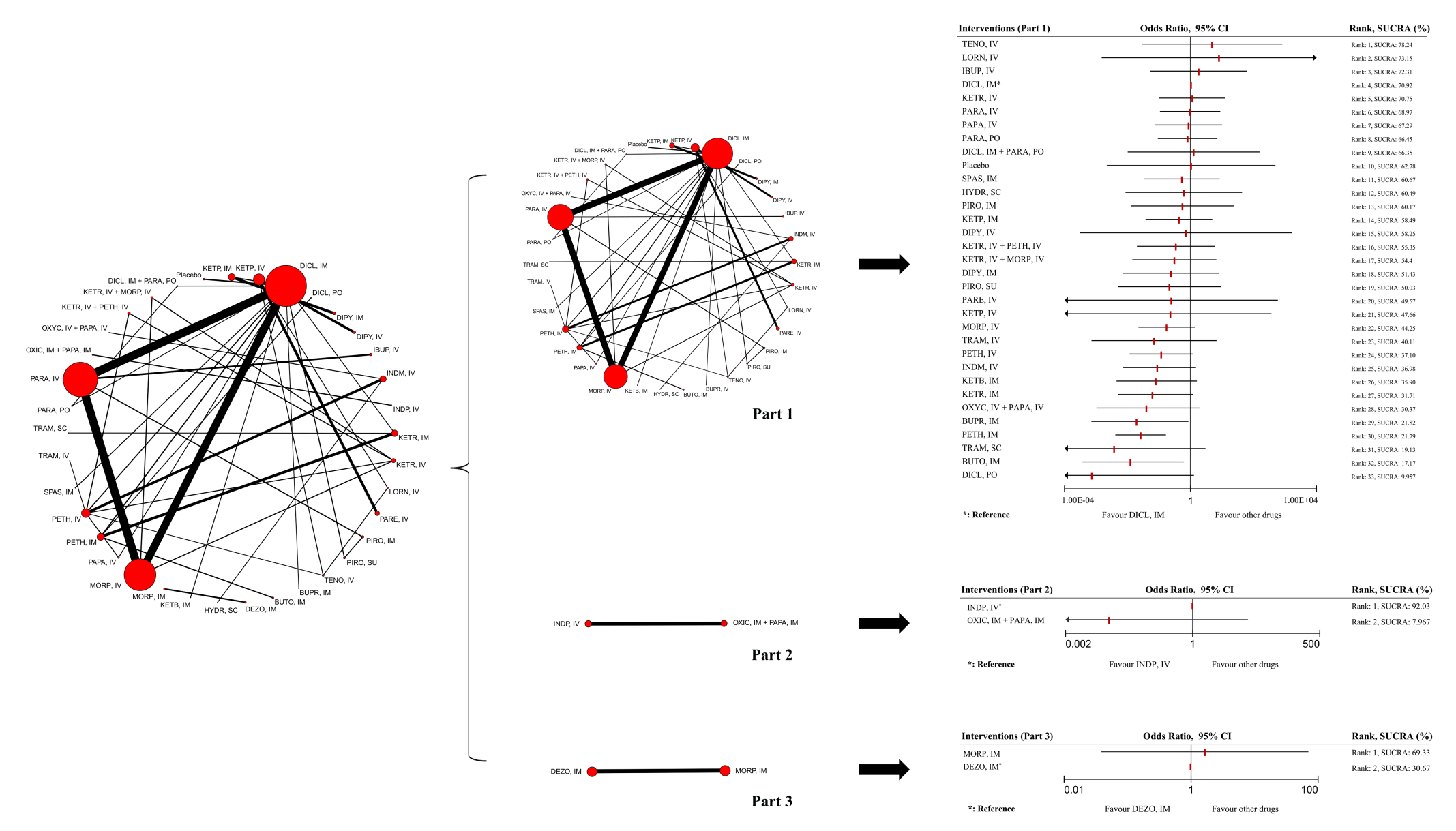
**

**Note:** The integrated information plot, including different drug branches and different routes, is composed of rude global network plot, natural punitive connecting network plots, forest plots, ranking, and their SUCRA values from network meta-analysis for nonspecific acute adverse events in third stage. In rude global network plot, natural punitive connecting network plots, the node sizes correspond to the number of accumulated sample size that investigated the treatments. Directly comparable treatments are linked with a line, and the thickness of the line corresponds to the sum of the sample size in each pairwise treatment comparison. SUCRA: Surface under the cumulative ranking, IM: Intramuscular route, IV: Intravenous route, PO: Per oral route, PR: Per rectal route, SC: Subcutaneous route, SU: Sublingual route. BUPR: Buprenorphine, BUTO: Butorphanol, DEZO: Dezocine, DICL: Diclofenac, DIPY: Dipyrone, HYDR: Hydromorphine IBUP: Ibuprofen, INDM: Indomethacin, INDP: Indoprofen, KETB: ketobemidone, KETP: Ketoprofen, KETR: Ketorolac, LORN: Lornoxicam, MORP: Morphine, OXIC: Oxicone, OXYC: Oxyconchloride, PAPA: Papaverine, PARA: Paracetamol, PARE: Parecoxib, PETH: Pethidine, PIRO: Piroxicam, SPAS: Spasmofen, TEMG: Temgesic, TENO: Tenoxicam, TRAM: Tramadol.

**Supplement Figure 15. Integrated information plots with different drug branches and routes for vomiting as an adverse event from third stage.**

**
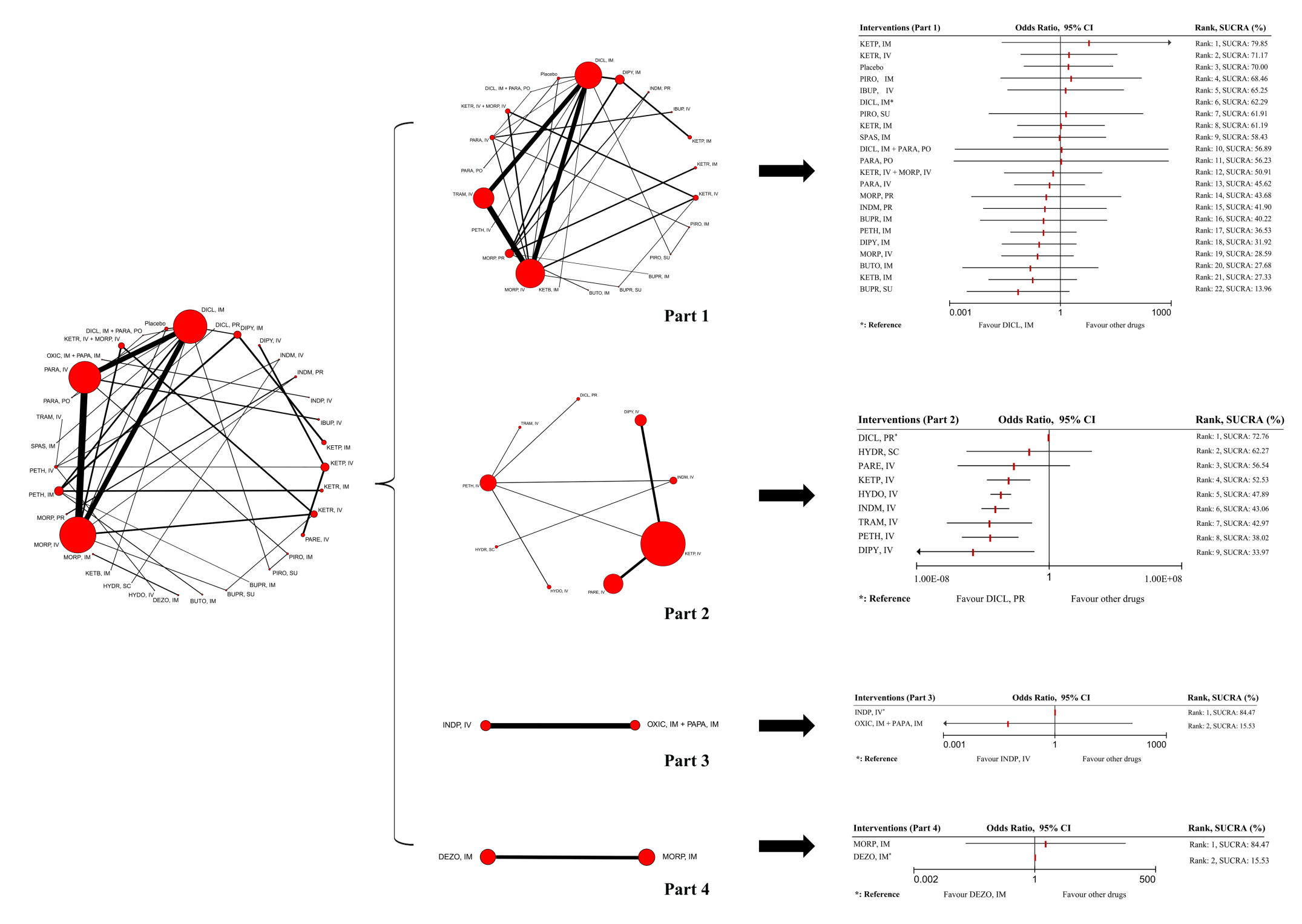
**

**Note:** The integrated information plot, including different drug branches and different routes, is composed of rude global network plot, natural punitive connecting network plots, forest plots, ranking, and their SUCRA values from network meta-analysis for vomiting as an adverse event in third stage. In rude global network plot, natural punitive connecting network plots, the node sizes correspond to the number of accumulated sample size that investigated the treatments. Directly comparable treatments are linked with a line, and the thickness of the line corresponds to the sum of the sample size in each pairwise treatment comparison. SUCRA: Surface under the cumulative ranking, IM: Intramuscular route, IV: Intravenous route, PO: Per oral route, PR: Per rectal route, SC: Subcutaneous route, SU: Sublingual route. BUPR: Buprenorphine, BUTO: Butorphanol, DEZO: Dezocine, DICL: Diclofenac, DIPY: Dipyrone, HYDO: Hydromorphone HYDR: Hydromorphine, IBUP: Ibuprofen, INDM: Indomethacin, INDP: Indoprofen, KETB: ketobemidone, KETP: Ketoprofen, KETR: Ketorolac, MORP: Morphine, OXIC: Oxicone, PAPA: Papaverine, PARA: Paracetamol, PARE: Parecoxib, PETH: Pethidine, PIRO: Piroxicam, SPAS: Spasmofen, TRAM: Tramadol.

**Supplement Figure 16. The SUCRA of co-linked active drugs for pain variance at 30 min and nonspecific acute adverse events in network meta-analyses from third stage.**

**
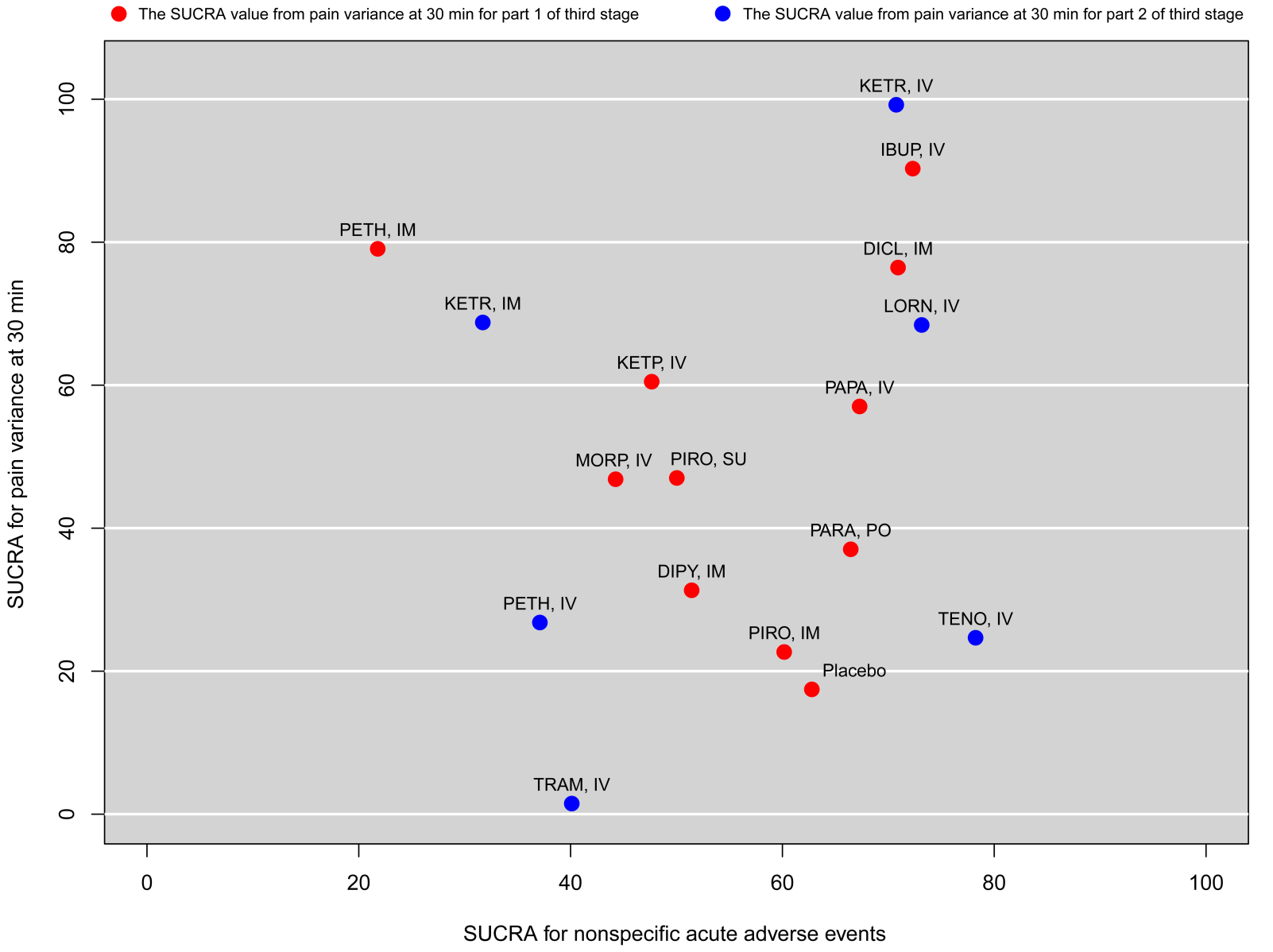
**

**Note:** All co-linked active drugs and placebo for all outcomes were ranked according to their SUCRA values. In the scatter plot, from best to worst, the higher SUCRA values demonstrate better effects or safer. The best intervention is in the upper right corner, while the worst is in the lower left corner. Red dot indicates the SUCRA value of Y-axis from pain variance at 30 min for part 1 of third stage, blue dot indicates the SUCRA value of Y-axis from pain variance at 30 min for part 2 of third stage. SUCRA: Surface under the cumulative ranking, IM: Intramuscular route, IV: Intravenous route, PO: Per oral route, PR: Per rectal route, SU: Sublingual route. DICL: Diclofenac, DIPY: Dipyrone, IBUP: Ibuprofen, KETP: Ketoprofen, KETR: Ketorolac, LORN: Lornoxicam, MORP: Morphine, PAPA: Papaverine, PARA: Paracetamol, PETH: Pethidine, PIRO: Piroxicam, TENO: Tenoxicam, TRAM: Tramadol.
